# Genomic Prediction of 16 Complex Disease Risks Including Heart Attack, Diabetes, Breast and Prostate Cancer

**DOI:** 10.1038/s41598-019-51258-x

**Published:** 2019-10-25

**Authors:** Louis Lello, Timothy G. Raben, Soke Yuen Yong, Laurent C. A. M. Tellier, Stephen D. H. Hsu

**Affiliations:** 1 Department of Physics and Astronomy, Michigan State University, East Lansing, Michigan USA; 2 Genomic Prediction, North Brunswick, NJ USA; 3 Cognitive Genomics Laboratory, Shenzhen Key Laboratory of Neurogenomics, China National GeneBank, BGI-Shenzhen, Shenzhen, China

**Keywords:** Machine learning, Disease genetics, Quantitative trait, Predictive markers, Risk factors

## Abstract

We construct risk predictors using polygenic scores (PGS) computed from common Single Nucleotide Polymorphisms (SNPs) for a number of complex disease conditions, using L1-penalized regression (also known as LASSO) on case-control data from UK Biobank. Among the disease conditions studied are Hypothyroidism, (Resistant) Hypertension, Type 1 and 2 Diabetes, Breast Cancer, Prostate Cancer, Testicular Cancer, Gallstones, Glaucoma, Gout, Atrial Fibrillation, High Cholesterol, Asthma, Basal Cell Carcinoma, Malignant Melanoma, and Heart Attack. We obtain values for the area under the receiver operating characteristic curves (AUC) in the range ~0.58–0.71 using SNP data alone. Substantially higher predictor AUCs are obtained when incorporating additional variables such as age and sex. Some SNP predictors alone are sufficient to identify outliers (e.g., in the 99th percentile of polygenic score, or PGS) with 3–8 times higher risk than typical individuals. We validate predictors out-of-sample using the eMERGE dataset, and also with different ancestry subgroups within the UK Biobank population. Our results indicate that substantial improvements in predictive power are attainable using training sets with larger case populations. We anticipate rapid improvement in genomic prediction as more case-control data become available for analysis.

## Introduction

Many important disease conditions are known to be significantly heritable^1^. This means that genomic predictors and risk estimates for a large number of diseases can be constructed if enough case-control data is available. In this paper we apply L1-penalized regression (LASSO) to case-control data from UK Biobank^2^ (UKBB) and construct disease risk predictors. Similar techniques have been used for phenotype prediction in plant and animal genomics, as described below, but are less familiar in the context of human complex traits and disease risks. (The promise of genetic prediction of human complex traits has been discussed for years^3–8^, but the use of genome wide predictors for common phenotypes has yet to become commonplace). In earlier work^9^, we applied these methods to quantitative traits such as height, bone density, and educational attainment. Our height predictor captures almost all of the expected heritability for height and has a prediction error of roughly a few centimeters. Similar methods have also been employed in previous work on case-control datasets^10,11^

The standard procedure for evaluating the performance of a genomic predictor is to construct the receiver operating characterstic (ROC) curve and compute the area under the ROC curve (AUC)^12^. Recently, Khera *et al*.^13^ constructed risk predictors for Atrial Fibrillation, Type 2 Diabetes, Breast Cancer, Inflammatory Bowel Disease, and Coronary Artery Disease (CAD). For these conditions, they obtained AUCs of 0.77, 0.72, 0.68, 0.63 and 0.81 respectively. Note, though, that additional variables such as age and sex are used to obtain these results. When common SNPs alone are used in the predictors, the corresponding AUCs are smaller. For example^14^, obtain an AUC of 0.64 for CAD using SNPs alone - compared with 0.81 with inclusion of age and sex found in^13^. (Note that references^13^ and^14^ contain non-overlapping results). See also^15^ for a CAD meta-analysis that also predicts risk stratification.

Among the disease conditions studied here are Hypothyroidism, Hypertension, Type 1 and 2 Diabetes, Breast Cancer, Prostate Cancer, Testicular Cancer, Gallstones, Glaucoma, Gout, Atrial Fibrillation, High Cholesterol, Asthma, Basal Cell Carcinoma, Malignant Melanoma and Heart Attack. We obtain AUCs in the range 0.580–0.707 (see Table 2), using SNP data alone. Substantially higher AUCs are obtained by incorporating additional variables such as age and sex. Some SNP predictors alone are sufficient to identify outliers (e.g., in the 99th percentile of polygenic score, or PGS) with, e.g., 3–8 times higher risk than typical individuals. We validate predictors out-of-sample using the eMERGE dataset^16^ (taken from the US population), and also with different ancestry subgroups within the UK Biobank population as done in^17^. Note that the disease conditions contain a mix of self reported and diagnosed conditions, described in Supplemental Section B, but we do not see any distinguishable difference in the results.

**Table 1:** Comparison of best known odds ratios in the literature (Literature) to the odds ratios calculated from UK BioBank data presented here (New).

Condition	Odds Ratio			
	PGS %	Literature	New	99% Predicted
Asthma	>96%	—	${2.71}_{-0.21}^{+0.21}$	${3.456}_{-0.002}^{+0.002}$
Atrial Fibrillation	>90%	${2.74}_{-0.22}^{+0.19}$* ^13^	${2.81}_{-0.24}^{+0.24}$	${10.8}_{-1.6}^{+2.1}$
Basal Cell Carcinoma	>96%	—	${2.64}_{-0.36}^{+0.36}$	${3.8}_{-0.54}^{+0.88}$
Breast Cancer	>96%	${2.36}_{-0.16}^{+0.18}$* ^13^	${1.799}_{-0.27}^{+0.27}$	${2.5}_{-0.10}^{+0.14}$
Gallstones	>96%	—	${2.41}_{-0.56}^{+0.56}$	${9.7}_{-2.1}^{+4.5}$
Glaucoma	>96%	—	${1.9}_{-0.53}^{+0.53}$	${2.5}_{-0.30}^{+0.16}$
Gout	>90%/<10%	${1.16}_{-0.03}^{+0.03}$^†^ ^59^	${8.2}_{-0.28}^{+0.32}$	${2.82}_{-0.24}^{+0.24}$
Heart Attack	>96%	—	${2.25}_{-0.37}^{+0.37}$	${2.7}_{-0.28}^{+0.52}$
High Cholesterol	>96%	—	${2.54}_{-0.27}^{+0.27}$	${2.29}_{-0.38}^{+0.58}$
Hypertension	>90%	${2.09}_{-0.23}^{+0.27}$^60^	${2.23}_{-0.02}^{+0.02}$	${3.35}_{-0.13}^{+0.13}$
Hypothyroidism	>96%	—	${4.13}_{-0.13}^{+0.13}$	${6.74}_{-0.36}^{+0.36}$
Malignant Melanoma	1*σ* shift	${1.36}_{-0.15}^{+0.16}$^61^	${1.35}_{-0.26}^{+0.26}$	${4.28}_{-0.98}^{+0.89}$
Prostate Cancer	>75%/<25%	${3.3}_{-0.6}^{+0.6}$* ^62^	${1.58}_{-0.34}^{+0.34}$	${4.6}_{-0.25}^{+0.33}$
Testicular Cancer	>96%	—	${1.73}_{-0.97}^{+0.97}$	${1.13}_{-0.42}^{+1.54}$
Type 1 Diabetes	>95%	22.8* ^63^	${4.22}_{-0.44}^{+0.44}$	${13.73}_{-0.79}^{+1.16}$
Type 2 Diabetes	>90%	${2.52}_{-0.17}^{+0.19}$* ^13^	${2.04}_{-0.05}^{+0.05}$	${2.81}_{-0.27}^{+0.27}$

Comparison was either made at the largest possible PGS common to the two sets, or using whatever definition of odds ratio was used in the literature (PGS %). Additionally we indicate what we predict the odds ratio will be for those with 99% scores or above (99% Predicted column). These predictions are found by assuming the data was drawn from Gaussian distributions. We confine our references to the literature to specifically genetic or polygenic risk score determination of odds ratios. Other biological risk factors could, in the future, be combined with genetic risk to generate even better prediction. Further details about the literature are found in Section E. We focus here on *purely genetic* predictors. For many traits we were unaware of previous odds ratio estimates based on a *purely* polygenic score. For those we were aware of we listed the largest odds ratio in the chart above. *These predictors include a regression on non-genetic biological information. ^†^This article appeared on the BioRxiv shortly before our manuscript and we were originally unaware of the results.

**Table 2:** Table of genetic AUCs using SNPs only - no age or sex.

Condition	Training Set	Test Set	AUC	Active SNPs	λ*
Hypothyroidism	impute	UKBB	0.705 (0.009)	3704 (41)	1.406e-06 (1.33e-7)
Hypothyroidism	impute	eMERGE	0.630 (0.006)		
Type 2 Diabetes	impute	UKBB	0.640 (0.015)	4168 (61)	6.93e-06 (1.73e-6)
Type 2 Diabetes	impute	eMERGE	0.633 (0.006)		
Hypertension	impute	UKBB	0.667 (0.012)	9674 (55)	4.46e-6 (4.86e-7)
Hypertension	impute	eMERGE	0.651 (0.007)		
Resistant Hypertension	impute	eMERGE	0.6861 (0.001)		
Asthma	calls	AA	0.632 (0.006)	3215 (16)	2.37e-6 (0.35e-6)
Type 1 Diabetes	calls	AA	0.647 (0.006)	50 (7)	7.9e-7 (0.1e-7)
Breast Cancer	calls	AA	0.582 (0.006)	480 (62)	3.38e-6 (0.05e-6)
Prostate Cancer	calls	AA	0.6399 (0.0077)	448 (347)	3.07e-6 (0.08e-8)
Testicular Cancer	calls	AA	0.65 (0.02)	19 (7)	1.42e-6 (0.04e-6)
Glaucoma	calls	AA	0.606 (0.006)	610 (114)	8.69e-7 (0.71e-7)
Gout	calls	AA	0.682 (0.007)	1010 (35)	9.41e-7 (0.03e-7)
Atrial Fibrillation	calls	AA	0.643 (0.006)	181 (39)	8.61e-7 (0.94e-7)
Gallstones	calls	AA	0.625 (0.006)	981 (163)	1.01e-7 (0.02e-7))
Heart Attack	calls	AA	0.591 (0.006)	1364 (49)	1.181e-6 (0.002e-7)
High Cholesterol	calls	AA	0.628 (0.006)	3543 (36)	2.4e-6 (0.2e-6)
Malignant Melanoma	calls	AA	0.580 (0.006)	26 (15)	9.5e-7 (0.8e-7)
Basal Cell Carcinoma	calls	AA	0.631 (0.006)	76 (22)	9.9e-7 (0.3e-7)

Training and validating is done using UKBB data from either direct calls or imputed data to match eMERGE. Testing is done with UKBB, eMERGE, or AA as described in Secs. 2 and Supplementary Information Sec. D. Numbers in parenthesis are the larger of either a standard deviation from central value or numerical precision as described in Sec. 2. λ* refers to the lasso *λ* value used to compute AUC as described in Sec. 2.

Our analysis indicates that substantial improvements in predictive power are attainable using training sets with larger case populations. We anticipate rapid improvement in genomic prediction as more case-control data become available for analysis.

It seems likely that genomic prediction of disease risk will, for a number of important disease conditions, soon be good enough to be applied broadly in a clinical setting^18–21^. Inexpensive genotyping (e.g., roughly $50 per sample for an array genotype which directly measures roughly a million SNPs, and allows imputation of millions more) can identify individuals who are outliers in risk score, and hence are candidates for additional diagnostic testing, close observation, or preventative intervention (e.g., behavior modification).

We note the successful application of similar methods in genomic prediction of plant and animal phenotypes. Earlier studies have shown some success on complex human disease risk using much smaller datasets and a variety of methods^22–24^. Early work in this direction can be found in, for example^25^, (which highlights the utility of what were then referred to as dense marker data sets)^3,26,27^, (genome-wide allele significance from association studies in additive models)^28–30^, (regression analysis), and^31^ (accounting for linkage disequilibrium). For more recent reviews, and the current status of these approaches for plant and animal breeding, see^32–34^.

## Methods and Data

The main dataset we use for training is the 2018 release of the UKBB^35^ (The 2018 version corrected some issues with imputation, included sex chromosomes, etc. See the Supplementary Information Sections A,B for further details). We use only genetically British individuals (as defined by UKBB using principal component analysis described in^36^) for training of our predictors. For out of sample testing, we use eMERGE data (restricted to self-reported white Americans) as well as self-reported white but non-genetically British individuals in UKBB. The specific eMERGE data set used here refers to data obtained from dbGaP, under accession phs000360.v3.p1. (https://www.ncbi.nlm.nih.gov/projects/gap/cgi-bin/study.cgi?study_id=phs000360.v3.p1). We refer to the latter testing method as Adjacent Ancestry (AA) testing: the individuals used are part of the UKBB dataset, but have not been used in training and differ in ancestry from the training population. (The AA testing is a procedure similar to that described in^17^, where it is argued that this kind of testing is valuable when true out of sample data is unavailable. Note this is *not* a detailed analysis of predictor power fall off as a function of ancestry genetic distance. We intend to report on such effects in a future study).

We construct linear models of genetic predisposition for a variety of disease conditions (There has been some attention to *non-linear* models for complex trait interaction in the literature^37–40^. However we limit ourselves here to additive effects, which have been shown to account for most of the common SNP heritability for human phenotypes such as height^9^, and in plant and animal phenotypes^41–43^). The phenotype data describes case-control status where cases are defined by whether the individual has been diagnosed for, or self-reports, the disease condition of interest. Our approach is built from previous work on compressed sensing^9,44,45^. In this earlier work we showed that matrices of human genomes are good “sensing matrices” in the terminology of compressed sensing. That is, the celebrated theorems resulting in performance guarantees and phase transition behavior of the *L*_1_ algorithms hold when human genome data are used^46–50^. Furthermore, *L*_1_ penalization efficiently captures essentially all the expected common SNP heritability for human height, one of the most complex but highly heritable human traits^9^. Additionally linear methods are capable of capturing most of the so-called “missing heritability”^51^. It is for these reasons that we focus specifically on *L*_1_ methods in this paper. Initial investigations into deep learning methods have shown that they do not universally outperform or even compete with linear methods^52^.

**Figure 1:**
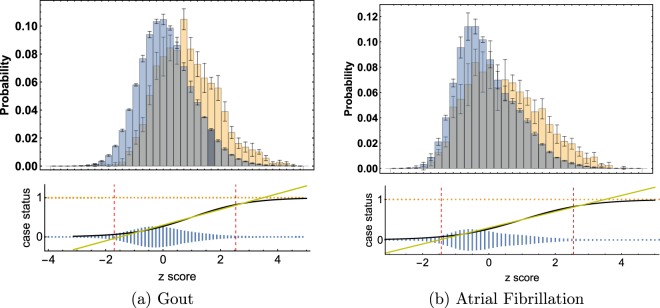
Top plots are histograms of controls (blue) and cases (gold). The bar heights are the averages over 5 AA testing runs. The error bars are standard deviations. On the bottom the same average case and control points are plotted on separate lines (1/0) for cases and controls. The height of the bars (gold and blue) represents the relative density of data points in that bin. Note that on the bottom, the gold and blue bars have been normalized using *the same* scale; the gold density looks small because most of the individuals in the data set are controls. The red dashed lines mark the 4% and 96% quartile of data, i.e. 92% of the data lies between those points. The x-axes are the same for top and bottom graphs: z scores, or number of standard deviations from the control mean. A linear (yellow) and logistic (black) curve are plotted over this range. It is clear that the difference between linear and logistic curves is negligible in the region where the data is concentrated.

Although we are focused on a classification problem of case/control conditions in this work, as can be seen in Fig. 1, the genetic scores of cases and controls have a large overlap. Because of this we found little difference in performance between linear vs logistic regression. We do not exclude the possibility that other methods (e.g.^53^) may work as well or better. *However*, *our primary motivation is the construction of potentially clinically useful predictors*, *not methodological comparison between different algorithms*.

We note that there are robust Bayesian Monte Carlo approaches that can account for a wide variety of model features like linkage disequilibrium and variable selection. However, it has been noted that (so far) for human complex traits, these methods have only produced a modest increase in predictive power at the cost of large computation times^54^. Our methods are not explicitly Bayesian; we estimate posterior uncertainties in our predictor via repeated cross-validation.

For each disease condition, we compute a set of additive effects ${\overrightarrow{\beta }}^{\ast }$ (each component is the effect size for a specific SNP) which minimizes the LASSO objective function:

2.1 $${{\mathscr{O}}}_{\lambda }(\overrightarrow{\beta })=\frac{1}{2}\parallel \overrightarrow{y}-X\overrightarrow{\beta }{\parallel }^{2}+n\lambda \parallel \overrightarrow{\beta }{\parallel }_{1};\,{\overrightarrow{\beta }}^{\ast }={{\rm{\min }}}_{\overrightarrow{\beta }\in {{\mathbb{R}}}^{p}}{O}_{\lambda }(\overrightarrow{y},X;\overrightarrow{\beta }),$$

where *p* is the number of regressands, *n* is the number of samples, $\parallel \ldots \parallel$ means *L*_2_ norm (square root of sum of squares), $\parallel \ldots {\parallel }_{1}$ is the *L*_1_ norm (sum of absolute values) and the term $\parallel \overrightarrow{\beta }{\parallel }_{1}$ is a penalization which enforces sparsity of $\overrightarrow{\beta }$. The optimization is performed over a space of 50,000 SNPs which are selected by rank ordering the p-values obtained from single-marker regression of the phenotype against the SNPs. The details of this are described in the Supplementary Information Section F.

Predictors are trained using a custom implementation of the LASSO algorithm which uses coordinate descent for a fixed value of *λ*. We typically use five non-overlapping sets of cases and controls held back from the training set for the purposes of in-sample cross-validation. For each value of *λ*, there is a particular predictor which is then applied to the cross-validation set, where the polygenic score is defined as (*i* labels the individual and *j* labels the SNP)

2.2 $${{\rm{PGS}}}_{i}=\sum _{j}\,{X}_{ij}{\beta }_{j}^{\ast }.$$

The term “polygenic score” typically refers to a simple measure built using results from single marker regression (e.g. GWAS), perhaps combined with p-value thresholding, and some method to account for linkage disequilibrium. Our use of penalized regression incorporates similar features – it favors sparse models (setting most effects to zero) in which the activated SNPs (those with non-zero effect sizes) are only weakly correlated to each other^9^. A thorough discussion of PGS construction is given in^55^. A brief overview of the use of single marker regression for phenotypes studied here is reviewed in Supplementary Information Section D.

To generate a specific value of the penalization λ* which defines our final predictor (for final evaluation on out-of-sample testing sets), we find the *λ* that maximizes AUC in each cross-validation set, average them, then move one standard deviation in the direction of higher penalization (the penalization *λ* is progressively reduced in a LASSO regression). Moving one standard deviation in the direction of higher penalization errs on the side of parsimony (In this context, a more parsimonious model refers to one with fewer active SNPs). These values of *λ* are reported in Section 4, but further analysis shows that tuning *λ* to a value that maximizes the testing set AUC tends to match λ* within error. This is explained in more detail in Supplementary Information F. The value of the phenotype variable *y* is simply 1 or 0 (for case or control status, respectively).

Scores can be turned into ROC curves by binning and counting cases and controls at various reference score values. The ROC curves are then numerically integrated to get AUC curves. We test the precision of this procedure by splitting ROC intervals into smaller and smaller bins. For several phenotypes this is compared to the rank-order (Mann-Whitney) exact AUC. The numerical integration, which was used to save computational time, gives AUC results accurate to ~1% (This is the given accuracy *at a specific number of cases and controls*. As described in Sec. 4 the absolute value of AUC depends on the number of reported cases). For various AUC results the error is reported as the larger of either this precision uncertainty or the statistical error of repeated trials.

Finally we note that for the analysis of case-control phenotypes it is common to use logistic regression. We studied this approach for those of our phenotypes that also appear in^13^, but found little to no difference in AUC or odds ratio results between linear and logistic regression. This might suggest that the data sets are highly constrained by the linear central region of the logistic function. Additionally, if we are simply interested in identifying genomes corresponding to *extreme* outliers, a linear regression can be more conservative.

## Utility of Genetic Predictors with Modest AUC

In this section we elaborate on the motivations for construction of predictors of complex disease risk. At the purely scientific level, the SNPs activated in the predictors give important clues to the genetic architecture and biochemical pathways involved in each condition. It is interesting that there is wide variation in genetic architecture: the number of SNPs activated can vary from a few dozen (e.g., for Type 1 Diabetes) to thousands (e.g., for Breast Cancer).

Beyond purely scientific interest, predictors of disease risk can have important practical applications. *It is important to note that the prediction AUC need not be especially high for the predictor to have utility*. *This is because a moderate AUC might still allow for the useful identification of individuals who are outliers in risk*.

Typically researchers quantify risk through an odds ratio of disease prevalence against a reference population. In Table 1, a summary of the odds ratios for various conditions examined in this work are computed and compared to the literature. Further details about how the odds ratios are calculated can be found below, in Section 4, and in the Supplementary Information Section G. A more in depth literature review can also be found in the Supplementary Information Section E.

**Figure 2:**
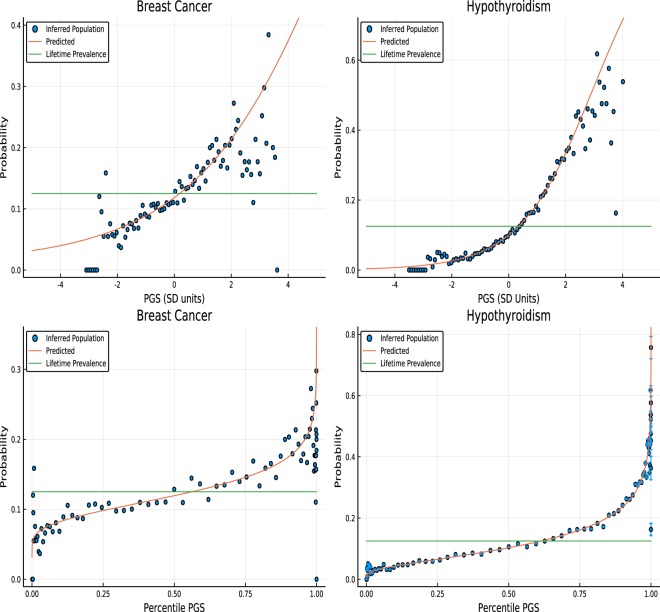
Probability of developing breast cancer or hypothyroidism given a specific polygenic score - shown in SD units and percentile. The lifetime population prevalence of both breast cancer and hypothyroidism are set to be 12%. Deviation from the red line, particularly at large and small PGS percentile, is likely an artifact of low statistics in these regions.

The utility of prediction can be illustrated using odds ratios. Here we examine odds ratios and show how they can be translated to different sub-populations or to a generic population as in Fig. 2. Consider the general population. Let *f*_1_(*z*) be the probability of polygenic score *z* in the case population, and ${f}_{0}(z)$ the corresponding probability for controls. Then the probability that a random individual has score *z* is

3.1 $$P(z)=\frac{{N}_{1}{f}_{1}(z)}{{N}_{1}+{N}_{0}}+\frac{{N}_{0}{f}_{0}(z)}{{N}_{1}+{N}_{0}}=\frac{{N}_{1}{f}_{1}(z)+{N}_{0}{f}_{0}(z)}{{N}_{1}+{N}_{0}}.$$

Again, this is the probability for the *general* population and *f*_1_ and *f*_0_ are generic distributions (i.e. we do not need to assume they are normal).

We can now consider representative sub-populations. Here, a representative sub-population means that for some sub-population, A, the number of cases and controls with score z is given by

3.2 $${n}_{1}^{A}(z)={N}_{1}^{A}{f}_{1}(z)\,\& \,{n}_{0}^{A}(z)={N}_{0}^{A}{f}_{0}(z),$$

where ${N}_{1}^{A}$ and ${N}_{0}^{A}$ are the total numbers of cases and controls in this sub-population.

From a sub-population we can construct a *binned* odds ratio, or BOR. The binned odds ratio is defined as the ratio number of cases to controls at a particular score value, normalized by the total number of cases and controls in the sub-population. If we examine two sub-populations, A and B, we see

3.3 $$BOR=\frac{{n}_{1}^{A}(z)/{n}_{0}^{A}(z)}{{N}_{1}^{A}/{N}_{0}^{A}}=\frac{1}{{r}_{A}}\frac{{n}_{1}^{A}(z)}{{n}_{0}^{A}(z)}=\frac{{f}_{1}(z)}{{f}_{0}(z)}=\frac{{n}_{1}^{B}(z)/{n}_{0}^{B}(z)}{{N}_{1}^{B}/{N}_{0}^{B}};\,{r}_{i}={N}_{1}^{i}/{N}_{0}^{i}$$

where we have used Eq. (3.2) to show that this BOR is *independent* of the number of cases and controls in the particular sub-population.

With these assumptions, the probability of developing a condition in one sub-population is given by

34 $${P}^{A}({\rm{case}}|{\rm{z}})=\frac{{{\rm{n}}}_{1}^{{\rm{A}}}({\rm{z}})}{{{\rm{n}}}_{0}^{{\rm{A}}}({\rm{z}})+{{\rm{n}}}_{1}^{{\rm{A}}}({\rm{z}})}=\frac{1}{1+\mathrm{1/(}{r}_{{\rm{A}}}\,\ast \,{\rm{BOR}})}$$

where the odds ratio can be calculated in the testing population. Then the probability of developing the condition in another population is given by

35 $${P}^{B}({\rm{case}}|{\rm{z}})=\frac{1}{1+1/({{\rm{r}}}_{{\rm{B}}}\,\ast \,{\rm{BOR}})}.$$

Using the odds ratio evaluated in the testing population and the (empirically known) lifetime prevalence of a specific condition, one can *estimate* the individual probability of developing a disease in the general population. We assume cases and controls are normally distributed in PGS score; we observed this to be empirically true (as described in the Supplementary Information Section E).

In Fig. 2, using the results of section 4, we display the probability that an individual will be diagnosed with Breast Cancer at some point in their life, conditional on PGS percentile. This is an absolute (genetic) risk – i.e., conditional on *only genetic* factors. Various risk models have been generated in the literature that involve genetic information, see for example the review^4^. While most models so far have focused on combinations of biological information with monogenic (GWAS) or genome-wide complex trait analysis, this work presents novel polygenic predictors which depend on genotype only. For individuals who are, e.g., in the top percentile in PGS, their risk is roughly 1 in 3, making them high risk by American Cancer Society guidelines. According to these guidelines, women with such PGS scores might be offered mammograms starting a decade earlier than women with average risk. Thus, *the Breast Cancer predictor may have practical utility already despite an AUC of only* 0.6 *or so*. *A similar conclusion may apply to some of the other predictors described in our paper*, *such as hypothyroidism*.

Future work should investigate the cost-benefit characteristics of population-level inexpensive genotyping. Below, we give a very simplified version of this kind of analysis, which suggests that the benefits from Breast Cancer screening alone might pay for the cost of genotyping the entire female population. Of course, such a significant conclusion requires much more detailed analysis than we provide here.

We can define a simple financial cost-benefit equation (per individual in the population) as follows:

3.6 $$X=\sum _{i}\,{T}_{i}({F}_{i}{B}_{i}-{C}_{i})-G.$$

Here the sum runs over different disease conditions *i* for which predictors have been developed, using genotyping data that costs *G* per individual. If the *i*-th item in the sum is not positive, we can simply opt not to use that specific disease condition. Under this assumption each term in the sum is either positive or zero.

*T*_*i*_ is defined to be a fraction of the population above a chosen PRS cutoff. *C*_*i*_ is the cost of an intervention (e.g., early mammograms) applied to all of these high risk individuals. *F*_*i*_ is the fraction of these high risk individuals who actually develop the condition (e.g., Breast Cancer), and *B*_*i*_ is the financial benefit to the health care system from early detection in those individuals.

In the case of breast cancer, we make the following estimates for these parameters. $G=\$100$ (inexpensive common SNP array), $T=0.01$ (top percentile in risk), $F=0.33$ (one in three develop Breast Cancer), $C=\$1000$ (cost of an extra decade of mammograms), and $B=\$30k$ (cost savings from early detection, estimated in^56^) [The potential for this kind of cost savings is already being discussed in non-technical sources, e.g. https://theconversation.com/population-dna-testing-for-disease-risk-is-coming-here-are-five-things-to-know-112522]. When these values are used in (3.6), the single term in the sum from breast cancer alone is similar in size to the $G=\$100$ cost of inexpensive genotyping. This suggests that population-level genotyping might already be cost-benefit positive given already available predictors.

Previous researchers have pushed for a similar approach^57^. In our view the above discussion provides strong motivation for our research, and future research, on the construction of PRS for a broad variety of disease conditions.

## Main Results

**Figure 3:**
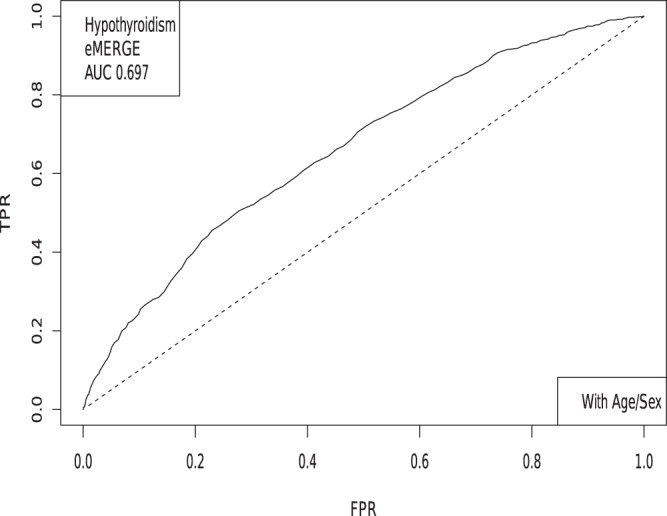
The receiver operator characteristic curve for case-control data on Hypothyroidism. This example includes sex and age as covariates.

**Figure 4:**
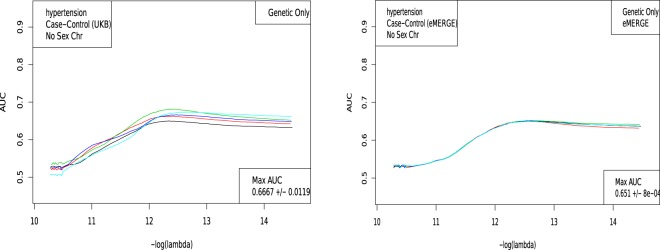
AUC computed on 5 holdback sets (1,000 each of cases and controls) for Hypertension, as a function of *λ*. A. UK Biobank and B. eMERGE.

The LASSO outputs can be used to build ROC curves, as shown in Fig. 3, and in turn produce AUCs and Odds Ratios. Figure 4 shows the evaluation of a predictor built using the LASSO algorithm. Five non-overlapping sets of cases and controls are held back from the training set for the purposes of in-sample cross-validation. For each value of *λ*, there is a particular predictor which is then applied to the cross-validation set. The value of *λ* one standard deviation higher than the one which maximizes AUC on a cross-validation set is selected as the definition of the model.Models are additionally judged by comparing a non-parametric measure, Mann-Whitney data AUC, to a parametric prediction, Gaussian AUC.

Each training set builds a slightly different predictor. After each of the 5 predictors is applied to the in-sample cross-validation sets, each model is evaluated (by AUC) to select the value of *λ* which will be used on the testing set. For some phenotypes we have access to true out-of-sample data (i.e. eMERGE), while for other phenotypes we implement adjacent ancestry (AA) testing using genetically dissimilar groups^17^. This is described in the Supplementary Information Sections C,D. An example of this type of calculation is shown in Fig. 4, where the AUC is plotted as a function of *λ* for Hypertension.

Table 2 below presents the results of similar analyses for a variety of disease conditions. We list the best AUC for a given trait and the data set which was used to obtain that AUC.

**Figure 5:**
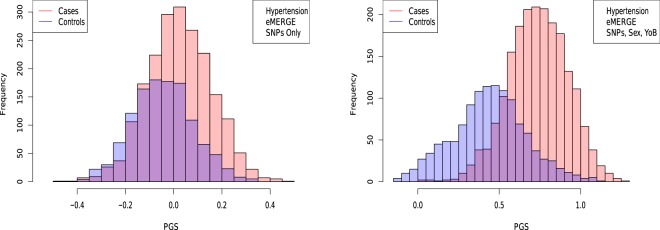
Distribution of PGS, cases and controls for Hypertension in the eMERGE dataset using SNPs alone and including sex and age as regressors.

**Figure 6:**
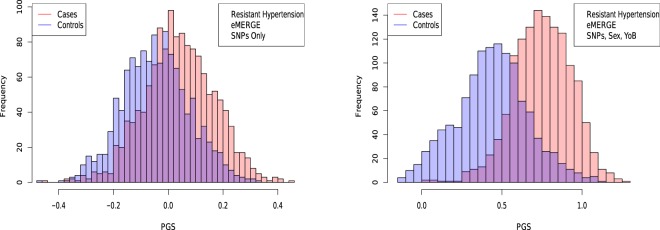
Distribution of PGS score, cases and controls for Resistant Hypertension in the eMERGE dataset using SNPs alone and including sex and age as regressors.

**Figure 7:**
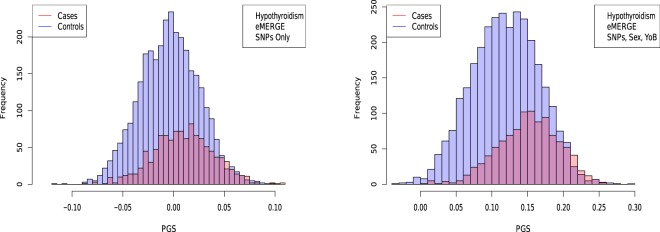
Distribution of PGS score, cases and controls for Hypothyroidism in the eMERGE dataset using SNPs alone and including sex and age as regressors.

**Figure 8:**
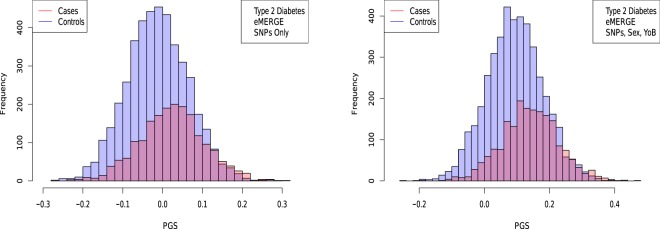
Distribution of PGS score, cases and controls for type 2 diabetes in the eMERGE dataset using SNPs alone and including sex and age as regressors.

In Figs 5, 6, 7 and 8, the distributions of the polygenic score are shown for cases and controls drawn from the eMERGE dataset. In each figure, we show on the left the distributions obtained from performing LASSO on case-control data only, and on the right an improved polygenic score which includes effects from separately regressing on sex and age. The improved polygenic score is obtained as follows: regress the phenotype $y=(1,0)$ against sex and age, and then add the resulting model to the LASSO score. This procedure is reasonable since SNP state, sex, and age are independent degrees of freedom. In some cases, this procedure leads to vastly improved performance.

The distribution of PGS among cases can be significantly displaced (e.g., shifted by a standard deviation or more) from that of controls when the AUC is high. At modest AUC, there is substantial overlap between the distributions, although the high-PGS population has a much higher concentration of cases than the rest of the population. Outlier individuals who are at high risk for the disease condition can therefore be identified by PGS score alone even at modest AUCs, for which the case and control normal distributions are displaced by, e.g., less than a standard deviation.

In Table 3 we compare results from regressions on SNPs alone, sex and age alone, and all three combined. Performance for some traits is significantly enhanced by inclusion of sex and age information.

**Table 3:** AUCs obtained using sex and age alone, SNPs alone, and all three together.

Condition	Test set	Age + Sex	Genetic Only	Age + Sex + genetic
Hypertension	UKBB	0.638 (0.018)	0.667 (0.012)	0.717 (0.007)
Hypothyroidism	UKBB	0.695 (0.007)	0.705 (0.009)	0.783 (0.008)
Type 2 Diabetes	UKBB	0.672 (0.009)	0.640 (0.015)	0.651 (0.013)
Hypertension	eMERGE	0.818 (0.008)	0.651 (0.007)	0.851 (0.009)
Resistant Hypertension	eMERGE	0.817 (0.008)	0.686 (0.007)	0.864 (0.009)
Hypothyroidism	eMERGE	0.643 (0.006)	0.630 (0.006)	0.697 (0.007)
Type 2 Diabetes	eMERGE	0.565 (0.006)	0.633 (0.006)	0.651 (0.007)

For example, Hypertension is predicted very well by age + sex alone compared to SNPs alone whereas Type 2 Diabetes is predicted very well by SNPs alone compared to age + sex alone. In all cases, the combined model outperforms either individual model.

The results thus far have focused on predictions built on the autosomes alone (i.e. SNPs from the sex chromosomes are not included in the regression). However, given that some conditions are predominant in one sex over the other, it seems possible that there is a nontrivial effect coming from the sex chromosomes. For instance, 85% of Hypothyroidism cases in the UK Biobank are women. In Table 4 we compare the results from including the sex chromosomes in the regression to using only the autosomes. The differences found in terms of AUC is negligible, suggesting that variation among common SNPs on the sex chromosomes does not have a large effect on Hypothyroidism risk. We found a similarly negligible change when including sex chromosomes for AA testing.

**Table 4:** AUCs with and without SNPs from the sex chromosomes.

Condition	With Sex Chr	No Sex Chr
Hypothyroidism	0.6302 (0.0012)	0.6300 (0.0012)
Type 2 Diabetes	0.6377 (0.0018)	0.6327 (0.0018)
Hypertension	0.6499 (0.0008)	0.6510 (0.0008)
Resistant Hypertension	0.6845 (0.001)	0.6861 (0.001)

All tested on eMERGE using SNPs as the only covariate.

Figures 5, 6, 7 and 8 suggest that case and control populations can be approximated by two overlapping normal distributions. Under this assumption, one can relate AUC directly to the means and standard deviations of the case and control populations. If two normal distributions with means *μ*_1_, *μ*_0_ and standard deviations *σ*_1_, *σ*_0_ are assumed for cases and controls ($i=1,0$ respectively below), the AUC can be explicitly calculated via (The details of the following calculations are in the Supplementary Information Section G. Some of the results can be found in^58^).

41 $$\begin{array}{rcl}f(x,{\mu }_{i},{\sigma }_{i}) & = & \frac{1}{\sqrt{2\pi {\sigma }_{i}^{2}}}\,\exp \,(-\frac{1}{2}{(\frac{x-{\mu }_{i}}{{\sigma }_{i}})}^{2})\\ \Phi (t) & = & {\int }_{-\infty }^{t}\,dx\,f(x,0,1)\\ {\rm{AUC}} & = & \Phi \,(\frac{{\mu }_{1}-{\mu }_{0}}{\sqrt{{\sigma }_{1}^{2}+{\sigma }_{0}^{2}}})\end{array}$$

Under the assumption of overlapping normal distributions, we can compute the following odds ratio OR(*z*) as a function of PGS. OR(*z*) is defined as the ratio of cases to controls for individuals with PGS ≥ *z* to the overall ratio of cases to controls in the entire population. In the formula below, 1 = cases, 0 = controls.

42 $${\rm{OR}}(z)=\frac{{\int }_{z}^{\infty }\,dx\,({n}_{1}{f}_{1}(x))/{\int }_{z}^{\infty }\,dx\,({n}_{0}{f}_{0}(x))}{{n}_{1}/{n}_{0}}=\frac{1-\Phi \,(\frac{z-{\mu }_{1}}{{\sigma }_{1}})}{1-\Phi \,(\frac{z-{\mu }_{0}}{{\sigma }_{0}})}$$

We compute means and standard deviations for cases and controls using the PGS distribution defined by the best predictor (by AUC) in the eMERGE dataset. We can then compare the AUC and OR predicted under the assumption of displaced normal distributions with the actual AUC and OR calculated directly from eMERGE data.

AUC results are shown in Table 5, where we assemble the statistics for predictors trained on SNPs alone. In Table 6 we do the same for predictors trained on SNPs, sex, and age.

**Table 5:** Mean and standard deviation for PGS distributions for cases and controls, using predictors built from SNPs only and trained on case-control status alone.

	Hypothyroidism	Type 2 Diabetes	Hypertension	Res HT
*μ*_*case*_	0.0093	0.0271	0.0240	0.0392
*μ*_*control*_	−0.0038	−0.0141	−0.0470	−0.0448
*σ*_*case*_	0.0284	0.0901	0.1343	0.1270
*σ*_*control*_	0.0276	0.0866	0.1281	0.1219
*N*_*cases*_/*N*_*controls*_	1,084/3,171	1,921/4,369	2,035/1,202	1,358/1,202
AUC_*pred*_	0.630 (0.006)	0.629 (0.006)	0.649 (0.006)	0.683 (0.007)
AUC_*actual*_	0.630 (0.006)	0.633 (0.006)	0.651 (0.007)	0.686 (0.006)

Predicted AUC from assumption of displaced normal distributions and actual AUC are also given.

**Table 6:** Mean and standard deviation for PGS distributions of cases and controls, using predictors built from SNPs, sex, and age, and trained on case-control status alone.

	Hypothyroidism	Type 2 Diabetes	Hypertension	Res HT
*μ*_*case*_	0.1516	0.1431	0.7377	0.7525
*μ*_*control*_	0.1185	0.0924	0.4375	0.4366
*σ*_*case*_	0.0437	0.0948	0.1829	0.1830
*σ*_*control*_	0.0474	0.0943	0.2250	0.2258
*N*_*cases*_/*N*_*controls*_	1,035/3,047	1,921/4,369	2,000/1,196	1,331/1,196
AUC_*pred*_	0.696 (0.007)	0.648 (0.006)	0.850 (0.009)	0.862 (0.009)
AUC_*actual*_	0.697 (0.007)	0.651 (0.007)	0.852 (0.009)	0.864 (0.009)

Predicted AUC from assumption of displaced normal distributions and actual AUC are also given.

**Figure 9:**
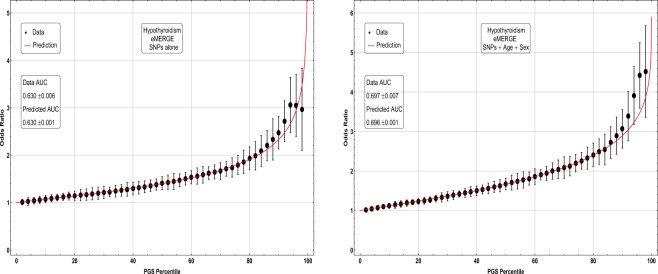
Odds ratio between upper percentile in PGS and total population prevalence in eMERGE for Hypothyroidism with and without using age and sex as covariates.

**Figure 10:**
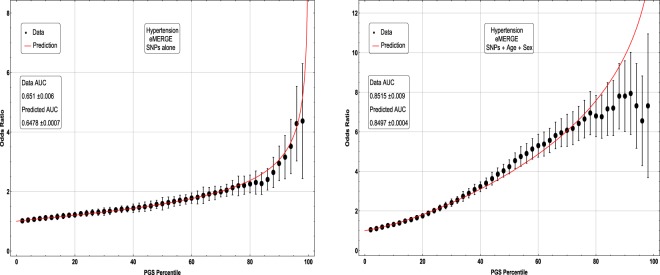
Odds ratio between upper percentile in PGS and total population prevalence in eMERGE for Hypertension with and without using age and sex as covariates.

**Figure 11:**
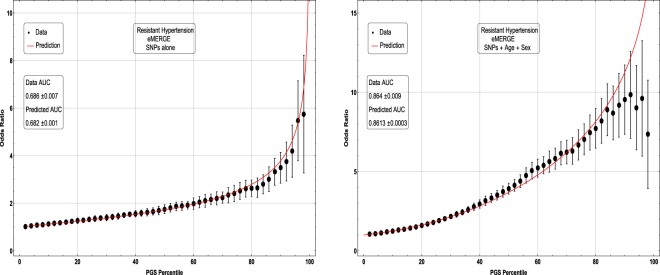
Odds ratio between upper percentile in PGS and total population prevalence in eMERGE for Resistant Hypertension with and without using age and sex as covariates.

**Figure 12:**
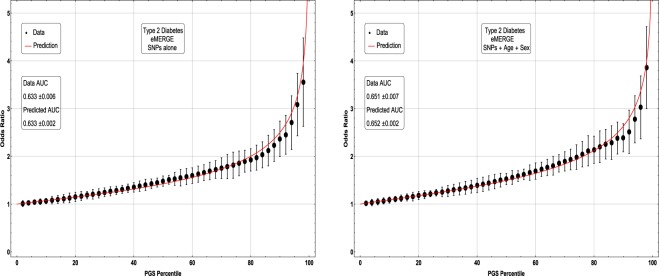
Odds ratio between upper percentile in PGS and total population prevalence in eMERGE for Type 2 Diabetes with and without using age and sex as covariates.

The results for odds ratios as a function of PGS percentile for several conditions are shown in Figs 9, 10, 11 and 12. Note that each figure shows the results when (1) performing LASSO on case-control data only and (2) adding a regression model on sex + age to the LASSO result. The red line is what one obtains using the assumption of displaced normal distributions, i.e. Equation 4.2, and for the rightmost graphs also contains information on age and sex. (Whether this approximation holds is of independent interest here. To the extent that it does, it allows simple extrapolation into the tail of the risk distribution). Overall there is good agreement between directly calculated odds ratios and the red line. Odds ratio error bars come from (1) repeated calculations using different training sets and (2) by assuming that counts of cases and controls are Poisson distributed. (This increases the error bar or estimated uncertainty significantly when the number of cases in a specific PGS bin is small).

The inclusion of the theoretically predicted red line in Figs 9, 10, 11 and 12 serves several purposes. Note, that in the higher PGS range, the fluctuations in the measured odds ratio become quite large - this is due to the small sample size in the higher PGS range - i.e., there are few data points available for individuals in the extreme range. The predicted values given by the red line provide a reasonable expectation for the odds ratios of individuals who fall in the high PGS tail of the distribution. This can be used to give estimated odds ratio targets for proposed future studies with higher counts of cases or for use in the interpretation of genetic testing. As mentioned above, much of the proposed clinical utility for PGS comes from risk stratification^57^, i.e. the hope to identify individuals at *high* or *low* risk. However, the cutoff for *high* risk is not a priori known and will vary from condition to condition. Another purpose of the red curves is to provide a rough test of the normality assumption - if the predicted curve and observed data deviate from each other substantially, this would provide some evidence that the normality assumption is invalid. Below we offer a *χ*^2^ test of the Gaussian nature of these distributions. While all conditions were well modeled with this distribution, this does not preclude the possibility that there are interesting non-Gaussian features.

In our analysis we tested whether altering the regressand (phenotype *y*) to some kind of residual based on age and sex could improve the genetic predictor. In all cases we start with $y=1,0$ for case or control respectively. Then we use the three different regressands:

4.3 $$y^{\prime} =y\,(y=1,0);\,{\rm{CC}}\,{\rm{status}}\,{\rm{alone}}$$

4.4 $$y^{\prime} =y-({\beta }_{0}+{\beta }_{S}S+{\beta }_{{\rm{Age}}}{\rm{Age}});\,{\rm{Modification}}\,{\rm{1}}$$

4.5 $$y^{\prime} =\frac{y-{\mu }_{M/F}}{{\sigma }_{M/F}}-({\beta }_{0}+{\beta }_{{\rm{Age}}}{\rm{Age}});\,{\rm{Modification}}\,{\rm{2}}$$

For each case, we tested this including and excluding the sex chromosomes during the regression. As with the previous results, the best prediction accuracy is not appreciably altered if training is done on the autosomes alone. The results are given in Table 7.

**Table 7:** Table of prediction results using three types of regressands.

Condition	CC Status	Mod 1	Mod 2
Hypothyroidism			
SNPs alone	0.6300 (0.0012)	0.6046 (0.0025)	0.6177 (0.0042)
Age/Sex Alone	0.6430		
With Age/Sex	0.6966 (0.0009)	0.6489 (0.0173)	0.6884 (0.0021)
Type 2 Diabetes			
SNPs alone	0.6327 (0.0018)	0.6378 (0.0018)	0.6327 (0.0018)
Age/Sex Alone	0.5654		
With Age/Sex	0.651 (0.0014)	0.6283 (0.0039)	0.651 (0.0014)
Hypertension			
SNPs alone	0.651 (0.0008)	0.6495 (0.0004)	0.6497 (0.0005)
Age/Sex Alone	0.8180		
With Age/Sex	0.8518 (0.0003)	0.8519 (0.0003)	0.8516 (0.0001)

All results are on eMERGE and show results for using SNPs, Age, Sex and combinations of such.

The distributions in Figs 5–7 appear Gaussian under casual inspection, and were further tested against a normal distribution. We illustrate this with Atrial Fibrillation and Testicular cancer - these two conditions represent respectively the best and worst fits to Gaussians. For control groups, results were similar for all phenotypes. For example assuming “Sturge’s Rule” for the number of bins, Atrial Fibrillation controls lead to ${\chi }_{dof}^{2}=5,359.29/56,772$ with a p-value $7\times {10}^{-1013}$ when tested against a Gaussian distribution. For cases, we also found extremely good fits. Again, Atrial Fibrillation cases lead to ${\chi }_{dof}^{2}=35.181/418$ and p-value 0.0192. Even for phenotypes with very few cases we find very good fits. For Testicular Cancer cases we find a ${\chi }_{dof}^{2}=35.1429/89$ and p-value 1.18 × 10^−4^. For predicted AUCs and Odds Ratios using Eqs (4.1) and (4.2) we find very little difference between using means and standard deviations from empirical data sets or using fits to Gaussians.

As more data become available for training we expect prediction strength (e.g., AUC) to increase. Based on estimated heritability, predictors in this study are still far from maximum possible AUCs, such as: type 2 diabetes (0.94), coronary artery disease (0.95), breast cancer (0.89), prostate cancer (0.90), and asthma (0.88)^12^. We investigate improvement with sample size by varying the number of cases used in training. For Type 2 Diabetes and Hypothyroidism, we train predictors with 5 random sets of 1k, 2k, 3k, 4k, 6k, 8k, 10k, 12k, 14k, and 16k cases (each of these trials uses the same total set of controls as described in the supplementary materials). For Hypertension, we train predictors using 5 randoms sets of 1k, 10k, 20k, …, and 90k cases. For each, we also include the previously generated best predictors which used all cases except the 1000 held back for cross-validation. These predictors are then applied to the eMERGE dataset and the maximum AUC is calculated.

**Figure 13:**
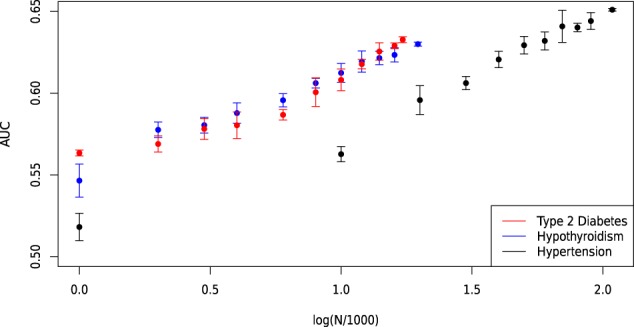
Maximum AUC on out-of-sample testing set (eMERGE) as a function of the number of cases (in thousands) included in training. Shown for type 2 diabetes, Hypothyroidism and Hypertension.

In order to gain a sense of how predictive capability improves with larger data sets, in Fig. 13 we plot the average maximum AUC among the 5 training sets against the log of the number of cases (in thousands) used in training. Note that in each situation, as the number of cases increases, so does the average AUC. For each disease condition, the AUC increases roughly linearly with log N as we approach the maximum number of cases available. Of course, this is just a rough observation but suggestive of a general trend. The main point is that there is no evidence of approach to an asymptotic (maximum) AUC with current levels of data. The rate of improvement for Type 2 Diabetes appears to greater than for Hypertension or Hypothyroidism, but in all cases there is no sign of diminishing returns. There is obviously a ceiling to the amount of improvement, determined by the heritability of the specific condition^12^, but we see no evidence that we are approaching that limit.

By extrapolating this linear trend, we can project the value of AUC obtainable using a future cohort with a larger number of cases. In this work, we trained Type 2 Diabetes, Hypothyroidism and Hypertension predictors using 17k, 20k and 108k cases, respectively. If, for example, three new cohorts were assembled with 100k, 100k and 500k cases of Type 2 Diabetes, Hypothyroidism and Hypertension respectively, then the linear extrapolation suggests AUC values of 0.70, 0.67 and 0.71 respectively. This corresponds to 95 percentile odds ratios of approximately 4.65, 3.5, and 5.2. In other words, it is reasonable to project that future predictors will be able to identify the 5 percent of the population with at least 3–5 times higher likelihood for these conditions than the general population. This will likely have important clinical applications, and we suggest that a high priority should be placed on assembling larger case data sets for important disease conditions.

We focused on the three traits above because we can test out of sample using eMERGE. However, using the adjacent ancestry (AA) method, we can make similar projections for diseases which may 1) be more clinically actionable or 2) show more promise for developing well separated cases and controls. We perform AA testing while varying the number of cases included in training for Type 1 Diabetes, Gout, and Prostate Cancer. We train predictors using all but 500, 1000, and 1500 cases and fit the maximum AUC to log(*N*/1000) to estimate AUC in hypothetical new datasets. For Type 1 Diabetes, we train with 2234, 1734 and 1234 cases - which achieve AUC of 0.646, 0.643, 0.642. For Gout we train with 5503, 5003 and 4503 cases achieving AUC of 0.0.681, 0.676, 0.0.673. For Prostate Cancer, we train with 2758, 2258, 1758 cases achieving AUC of 0.0.633, 0.628, 0.609. A linear extrapolation to 50k cases of Prostate Cancer, Gout, and Type 1 Diabetes suggests that new predictors could achieve AUCs of 0.79, 0.76 and 0.66 (respectively) based solely on genetics. Such AUCs correspond to odds ratios of and 11, 8, and 3.3 (respectively) for 95th percentile PGS score and above.

## Discussion

The significant heritability of most common disease conditions implies that at least some of the variance in risk is due to genetic effects. With enough training data, modern machine learning techniques enable us to construct polygenic predictors of risk. A learning algorithm with enough examples to train on can eventually identify individuals, based on genotype alone, who are at unusually high risk for the condition. This has obvious clinical applications: scarce resources for prevention and diagnosis can be more efficiently allocated if high risk individuals can be identified while still negative for the disease condition. This identification can occur early in life, or even before birth.

In this paper we used UK Biobank data to construct predictors for a number of conditions. We conducted out of sample testing using eMERGE data (collected from the US population) and adjacent ancestry (AA) testing using UK ethnic subgroups distinct from the training population. The results suggest that our polygenic scores indeed predict complex disease risk - there is very strong agreement in performance between the training and out of sample testing populations. Furthermore, in both the training and test populations the distribution of PGS is approximately Gaussian, with cases having on average higher scores. We verify that, for all disease conditions studied, a simple model of displaced Gaussian distributions predicts empirically observed odds ratios (i.e., individual risk in test population) as a function of PGS. This is strong evidence that the polygenic score itself, generated for each disease condition using machine learning, is indeed capturing a nontrivial component of genetic risk.

By varying the amount of case data used in training, we estimate the rate of improvement of polygenic predictors with sample size. Plausible extrapolations suggest that sample sizes readily within reach of population genetics studies will result in predictors of significant clinical utility. Additionally, extending this analysis to exome and whole genome data will also improve prediction. The use of genomics in Precision Medicine has a bright future, which is just beginning. We believe there is a strong case for making inexpensive genotyping Standard of Care in health systems across the world.

## Supplementary information

LaTeX Supplementary File

LaTeX Supplementary File

LaTeX Supplementary File

LaTeX Supplementary File

LaTeX Supplementary File

LaTeX Supplementary File

LaTeX Supplementary File

LaTeX Supplementary File

LaTeX Supplementary File

LaTeX Supplementary File

LaTeX Supplementary File

LaTeX Supplementary File

LaTeX Supplementary File

LaTeX Supplementary File

LaTeX Supplementary File

LaTeX Supplementary File

LaTeX Supplementary File

LaTeX Supplementary File

LaTeX Supplementary File

LaTeX Supplementary File

LaTeX Supplementary File

LaTeX Supplementary File

LaTeX Supplementary File

LaTeX Supplementary File

LaTeX Supplementary File

LaTeX Supplementary File

LaTeX Supplementary File

LaTeX Supplementary File

LaTeX Supplementary File

LaTeX Supplementary File

LaTeX Supplementary File

LaTeX Supplementary File

LaTeX Supplementary File

LaTeX Supplementary File

LaTeX Supplementary File

LaTeX Supplementary File

LaTeX Supplementary File

LaTeX Supplementary File

LaTeX Supplementary File

LaTeX Supplementary File

LaTeX Supplementary File

LaTeX Supplementary File

LaTeX Supplementary File

LaTeX Supplementary File

LaTeX Supplementary File

LaTeX Supplementary File

LaTeX Supplementary File

LaTeX Supplementary File

LaTeX Supplementary File

Supplementary Info
