# Author Correction: Genomic Prediction of 16 Complex Disease Risks Including Heart Attack, Diabetes, Breast and Prostate Cancer

**DOI:** 10.1038/s41598-019-54426-1

**Published:** 2019-11-20

**Authors:** Louis Lello, Timothy G. Raben, Soke Yuen Yong, Laurent C. A. M. Tellier, Stephen D. H. Hsu

**Affiliations:** 1 Department of Physics and Astronomy, Michigan State University, East Lansing, Michigan USA; 2 Genomic Prediction, North Brunswick, NJ USA; 3 Cognitive Genomics Laboratory, Shenzhen Key Laboratory of Neurogenomics, China National GeneBank, BGI-Shenzhen, Shenzhen, China

Correction to: *Scientific Reports* 10.1038/s41598-019-51258-x, published online 25 October 2019

In this Article, the authors neglected to include some potential conflicts of interest. The Competing Interests section should read:

“Laurent Tellier is an employee, shareholder, and serves on the board of directors of the company Genomic Prediction, Inc. Stephen Hsu is a shareholder and serves on the board of directors of Genomic Prediction, Inc. Louis Lello had no affiliation with Genomic Prediction, Inc. during the research, writing, and submission of this paper, but joined the company just before the paper was accepted. The other authors have no commercial interests relevant to the research.”

