# Supplementary figures and images for: Genomic Prediction of 16 Complex Disease Risks Including Heart Attack, Diabetes, Breast and Prostate Cancer
Source: Sci Rep. 2019 Oct 25;9:15286. doi: 10.1038/s41598-019-51258-x (PMC6814833; doi:10.1038/s41598-019-51258-x)

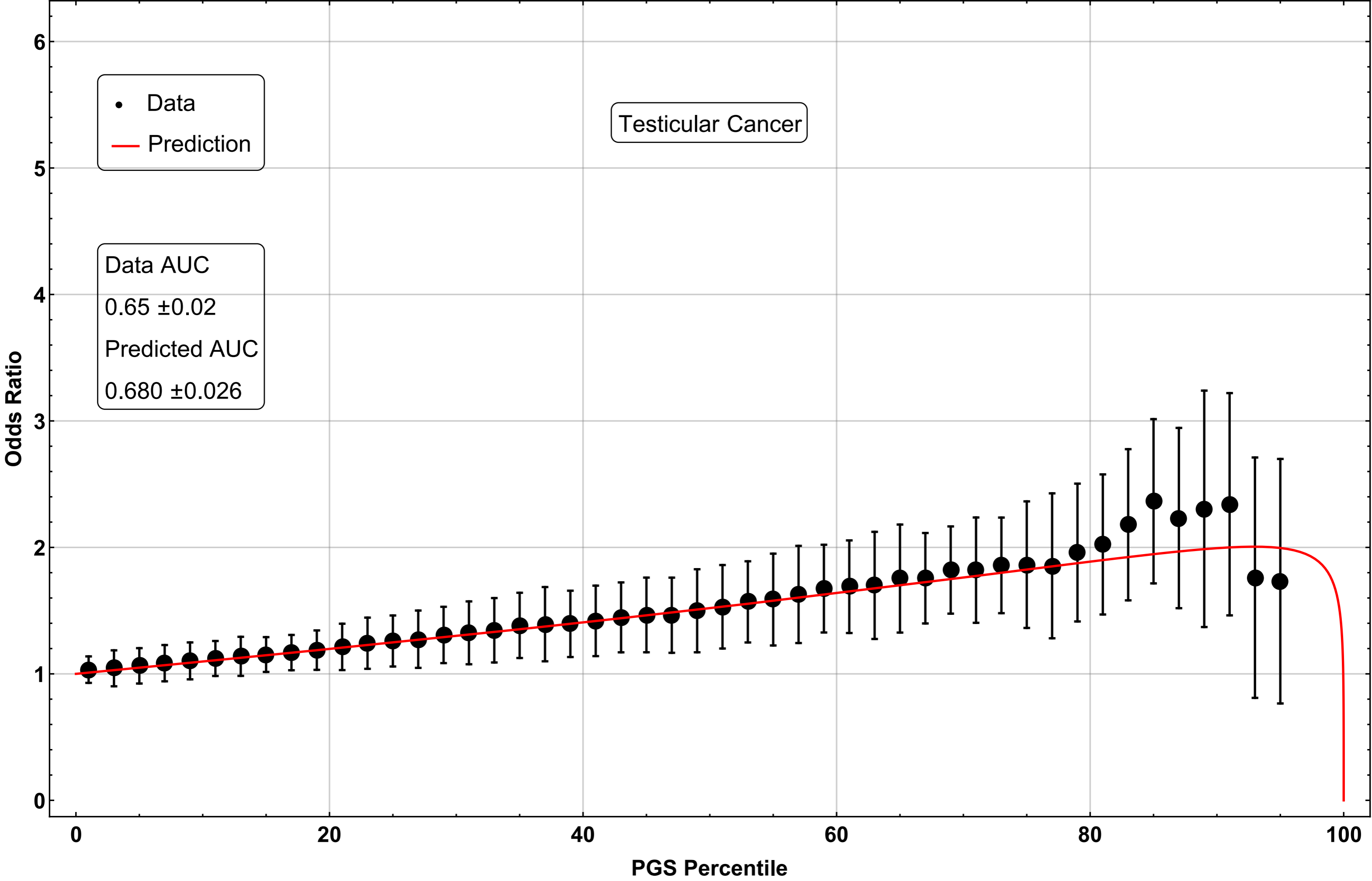

Supplement: Supplementary file 1 — LaTeX Supplementary File [file 41598_2019_51258_MOESM1_ESM.pdf]

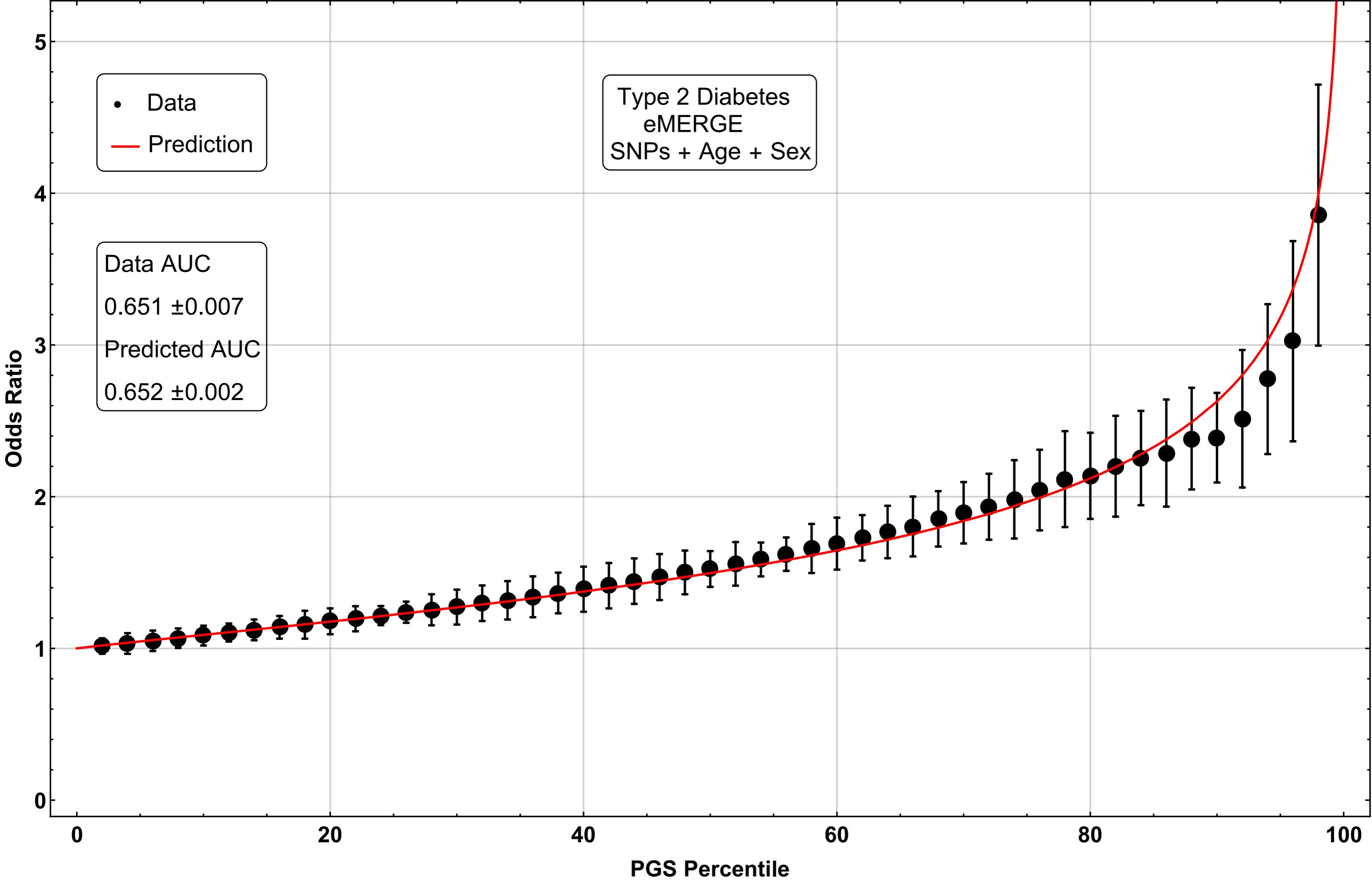

Supplement: Supplementary file 2 — LaTeX Supplementary File [file 41598_2019_51258_MOESM2_ESM.pdf]

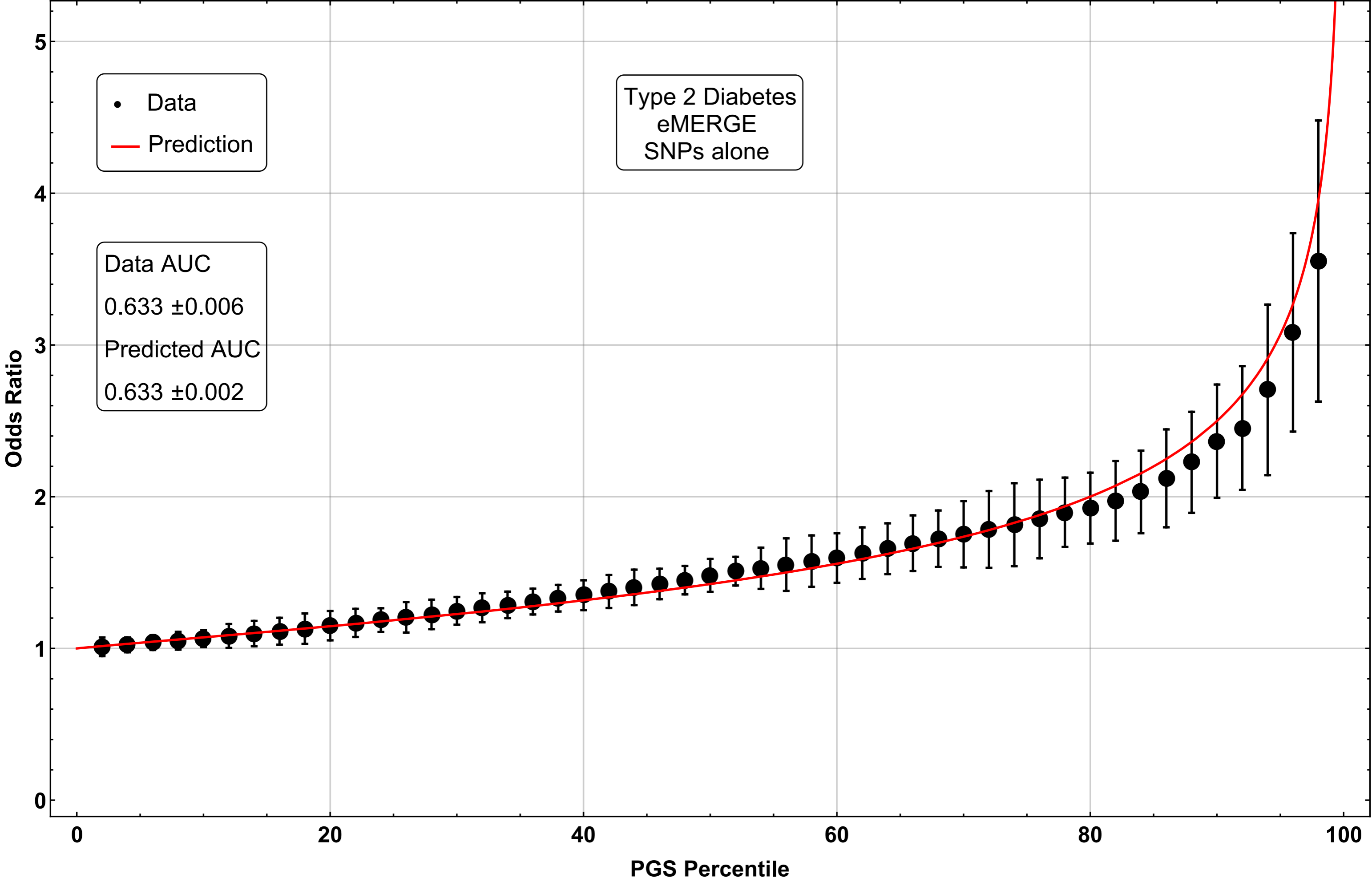

Supplement: Supplementary file 3 — LaTeX Supplementary File [file 41598_2019_51258_MOESM3_ESM.pdf]

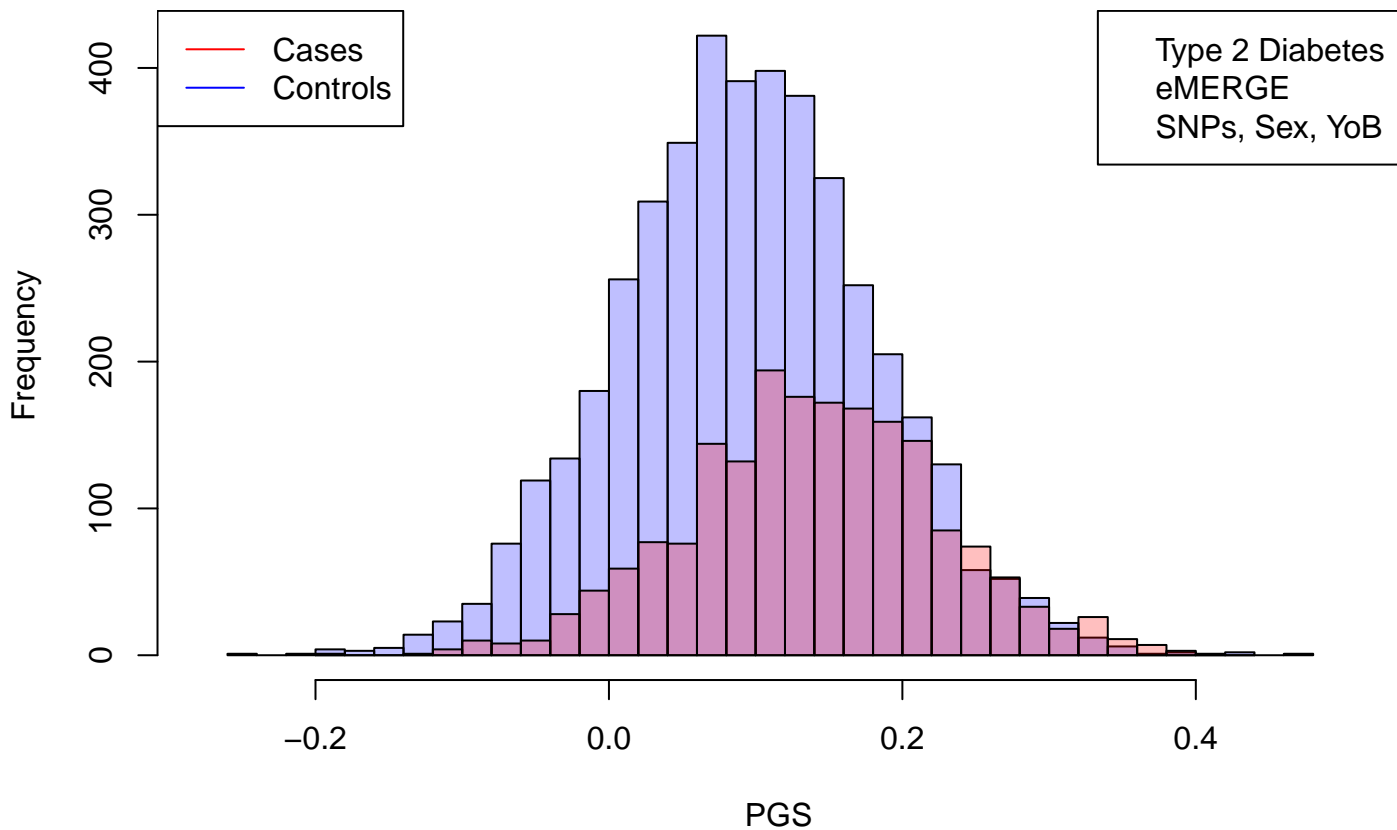

Supplement: Supplementary file 4 — LaTeX Supplementary File [file 41598_2019_51258_MOESM4_ESM.pdf]

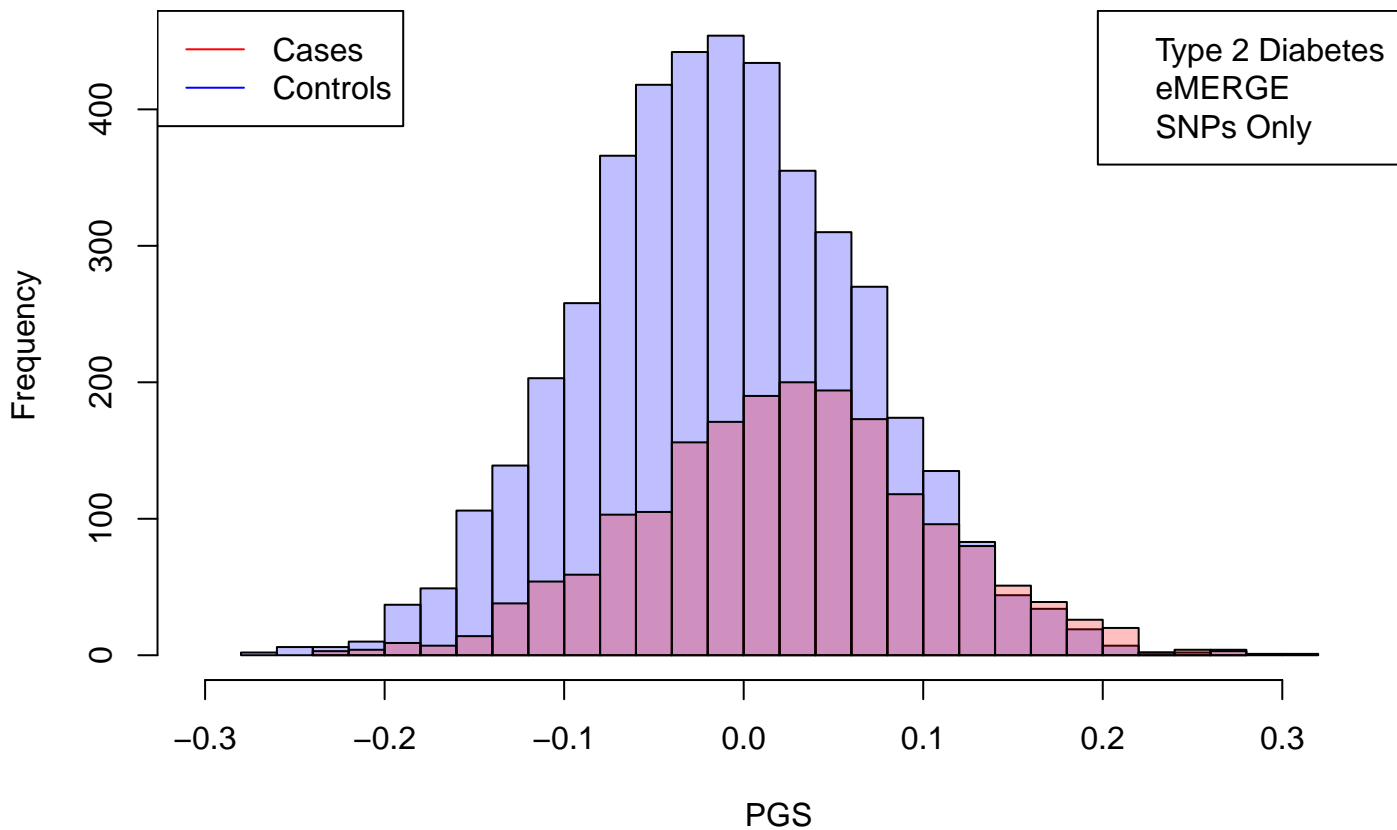

Supplement: Supplementary file 5 — LaTeX Supplementary File [file 41598_2019_51258_MOESM5_ESM.pdf]

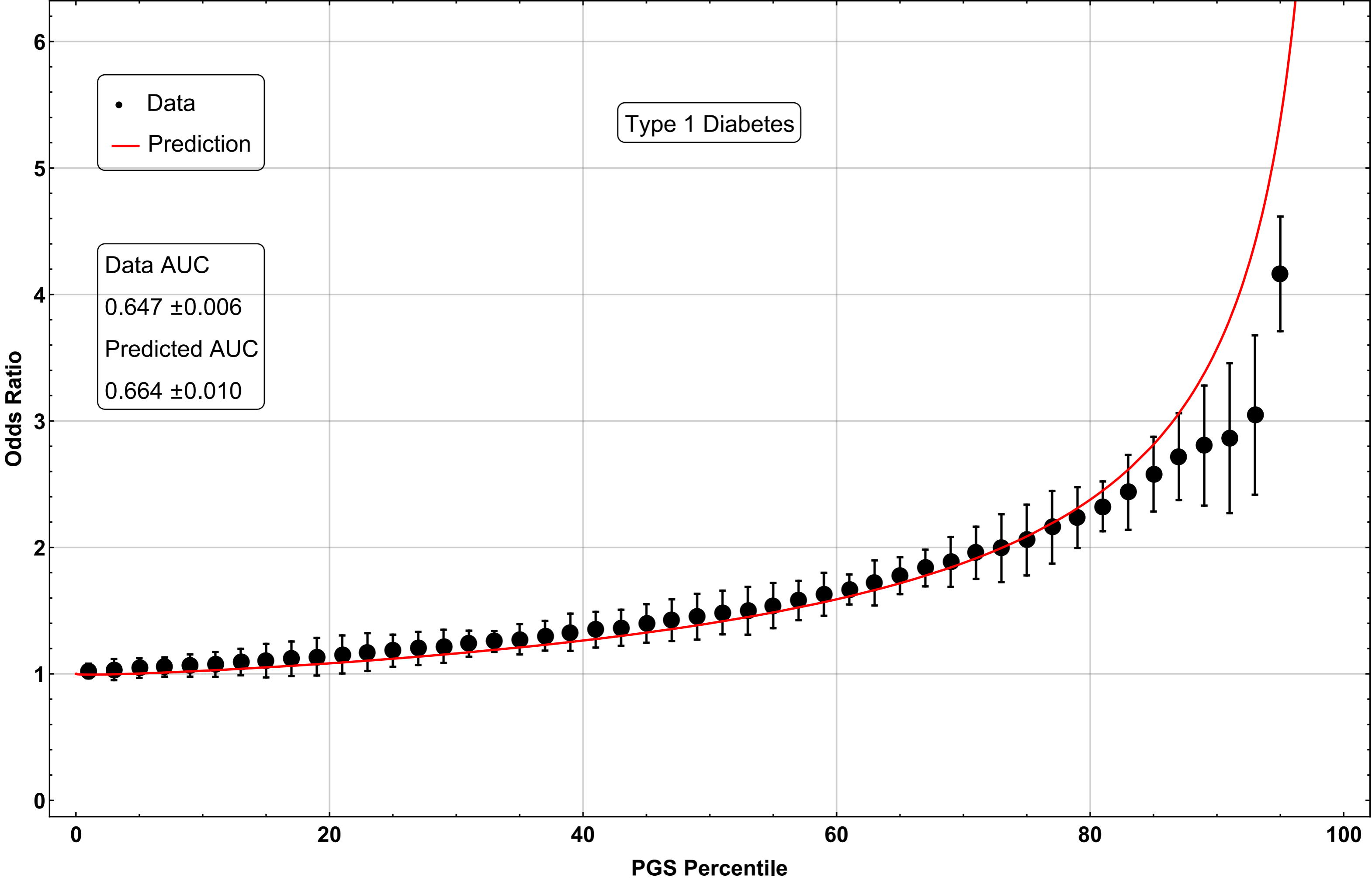

Supplement: Supplementary file 6 — LaTeX Supplementary File [file 41598_2019_51258_MOESM6_ESM.pdf]

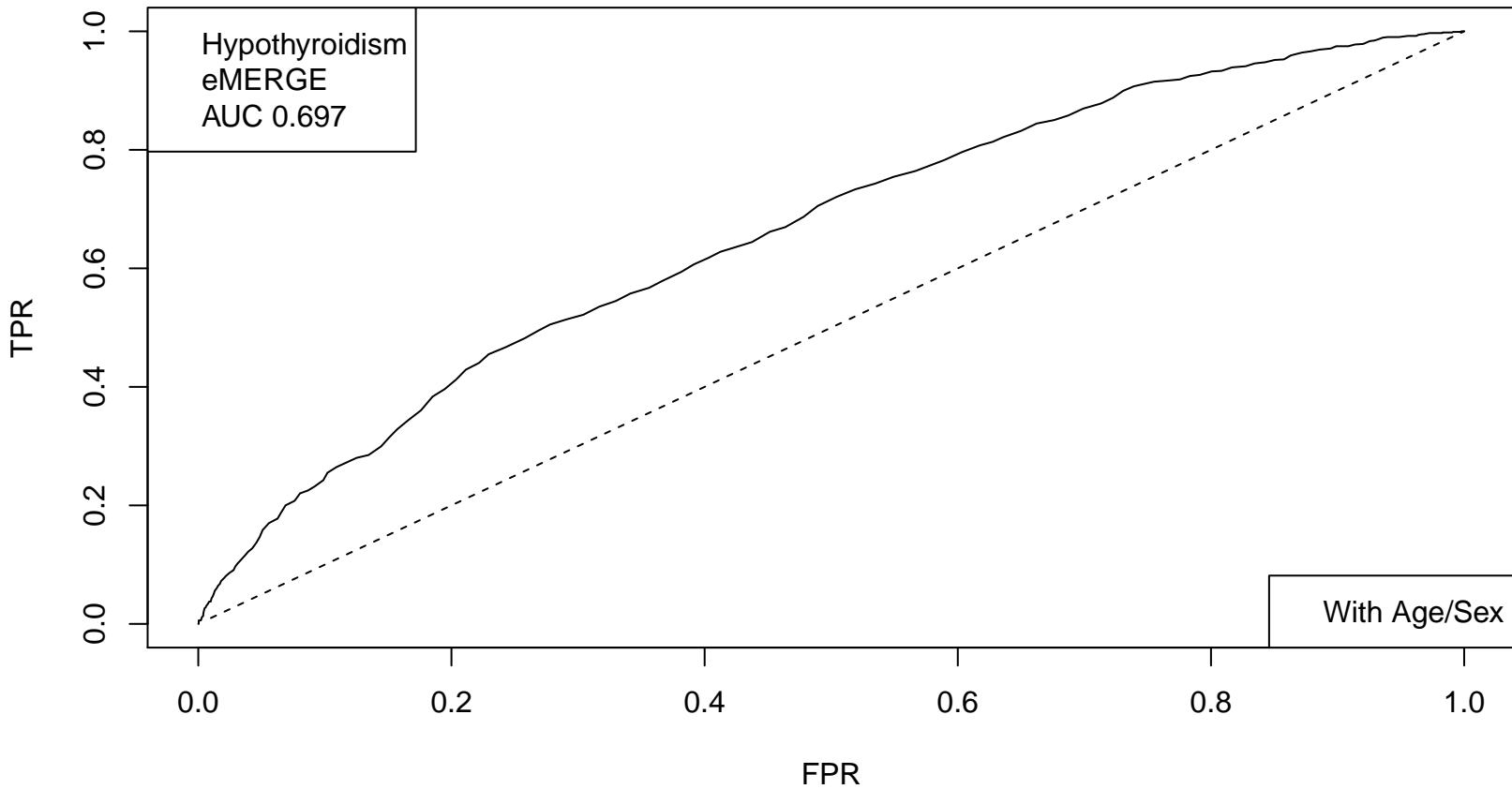

Supplement: Supplementary file 7 — LaTeX Supplementary File [file 41598_2019_51258_MOESM7_ESM.pdf]

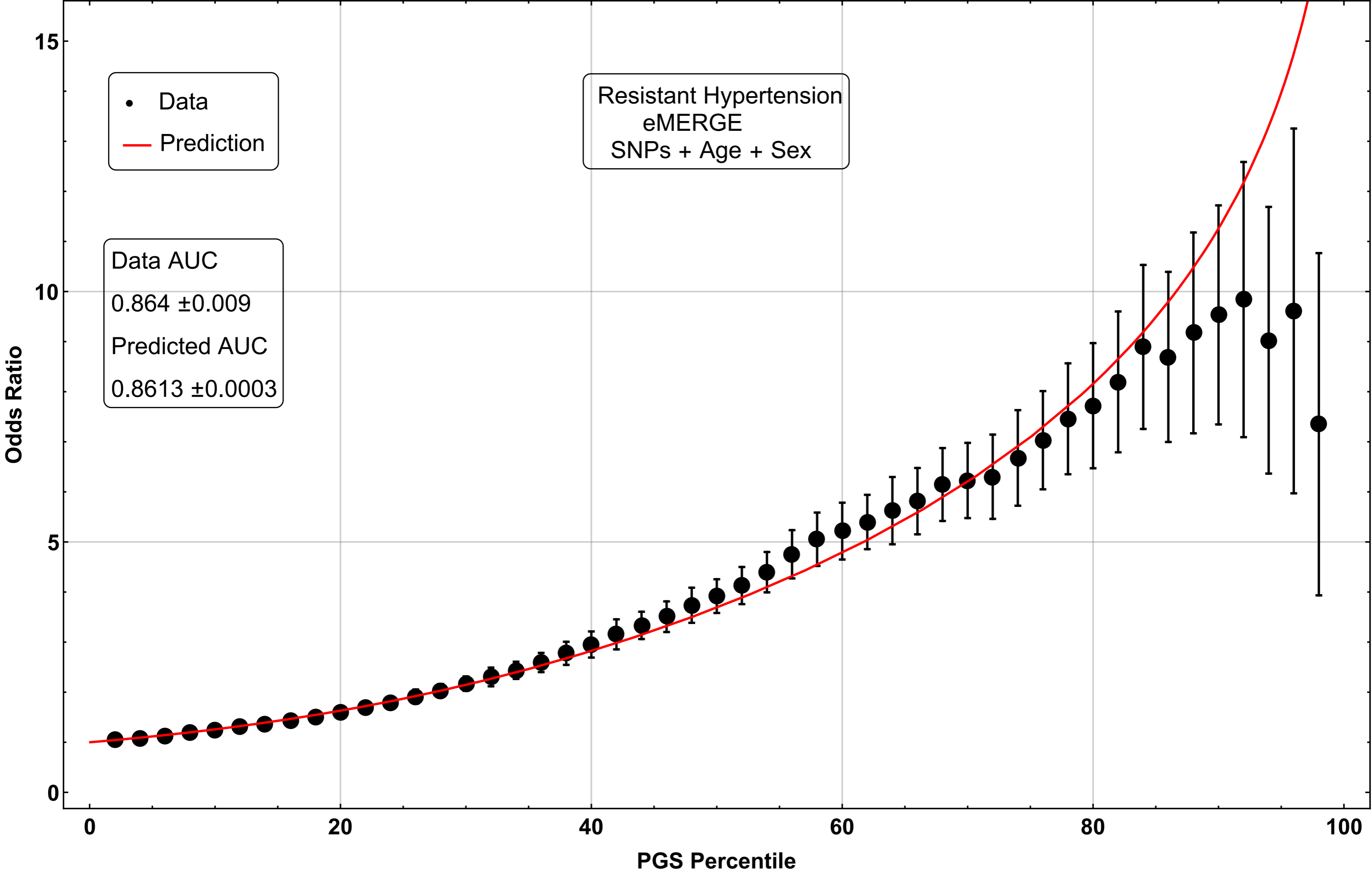

Supplement: Supplementary file 8 — LaTeX Supplementary File [file 41598_2019_51258_MOESM8_ESM.pdf]

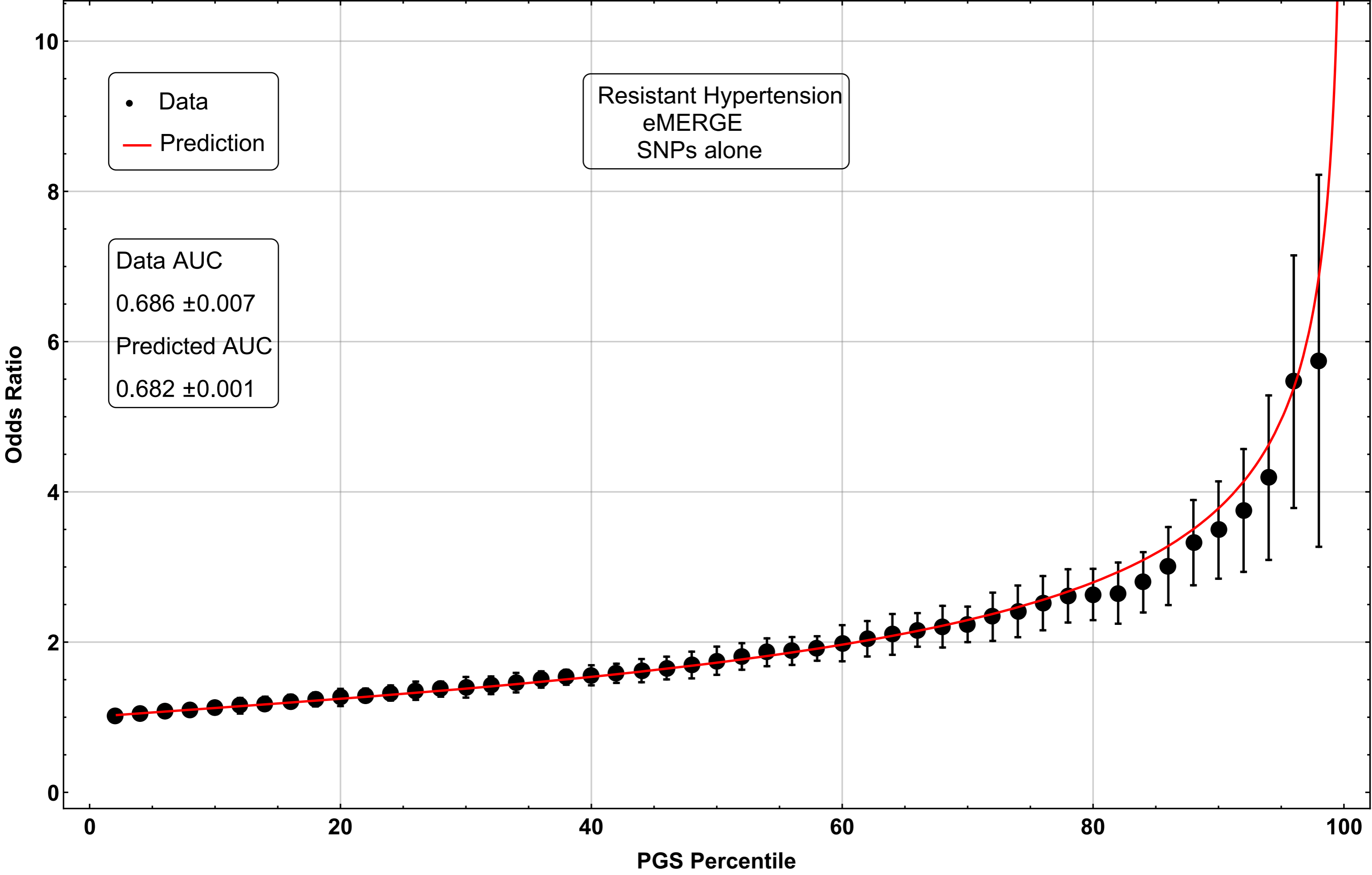

Supplement: Supplementary file 9 — LaTeX Supplementary File [file 41598_2019_51258_MOESM9_ESM.pdf]

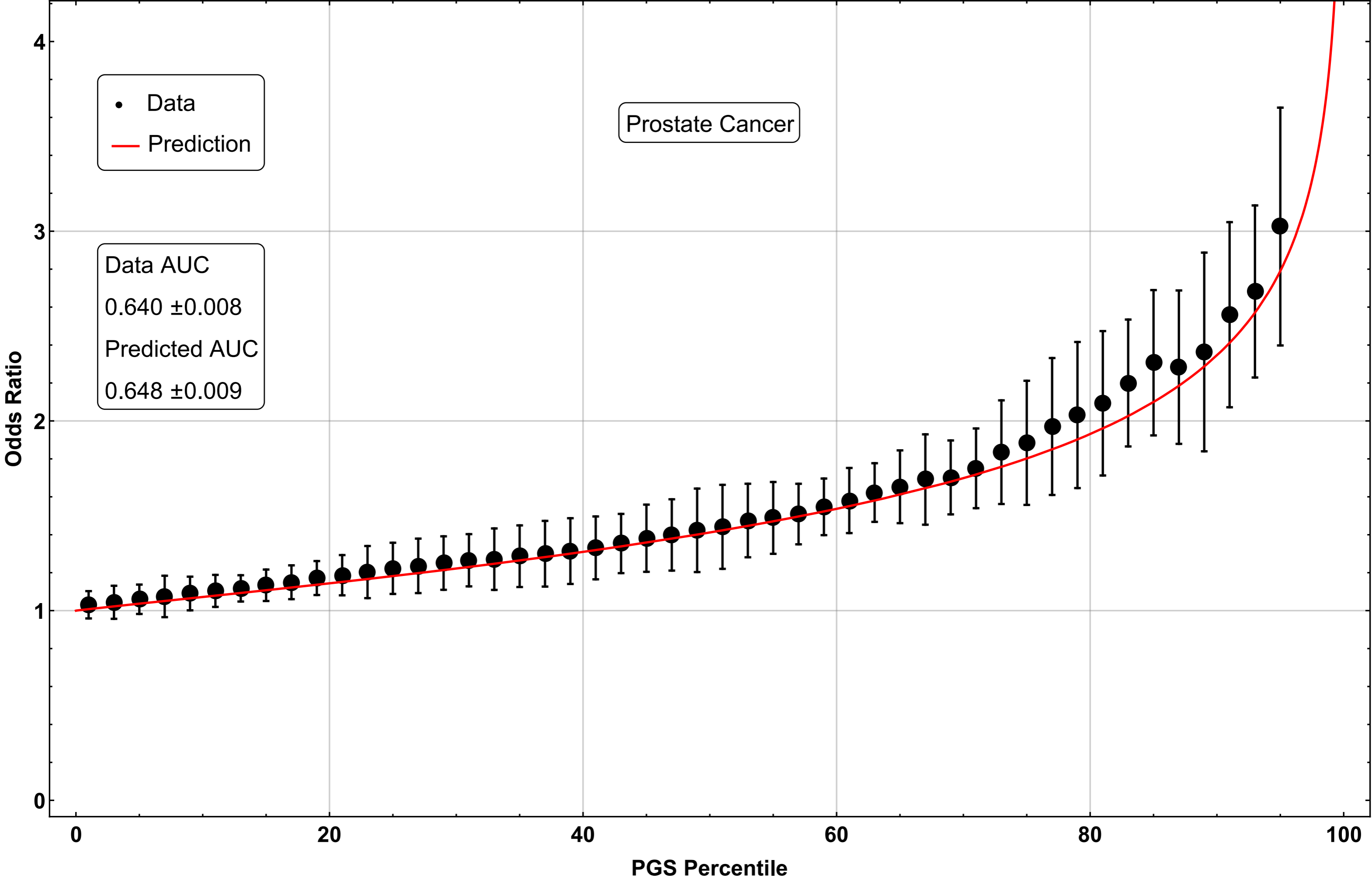

Supplement: Supplementary file 10 — LaTeX Supplementary File [file 41598_2019_51258_MOESM10_ESM.pdf]

# Hypothyroidism

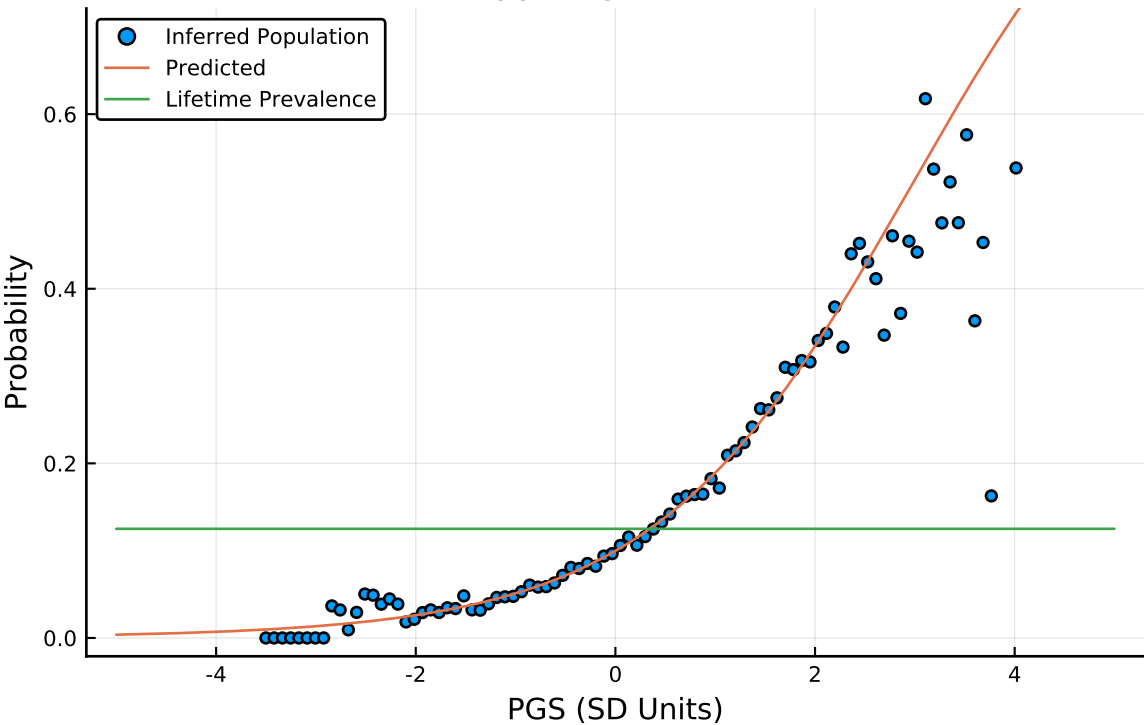

Supplement: Supplementary file 11 — LaTeX Supplementary File [file 41598_2019_51258_MOESM11_ESM.pdf]

# Breast Cancer

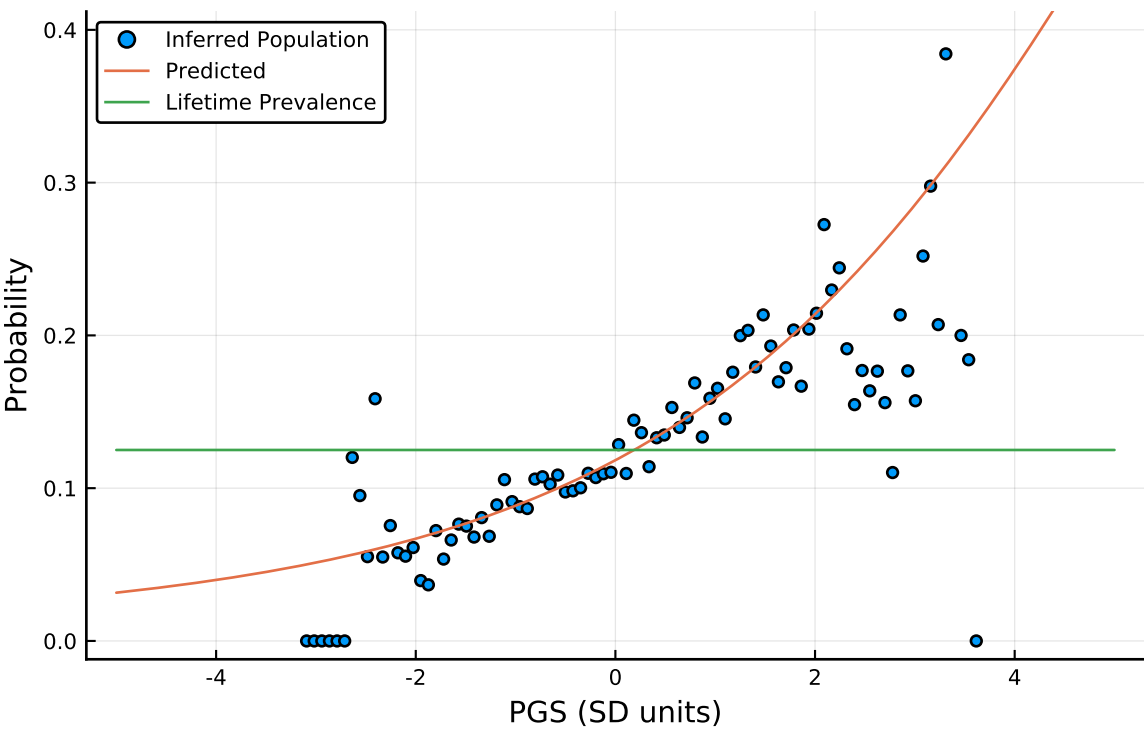

Supplement: Supplementary file 12 — LaTeX Supplementary File [file 41598_2019_51258_MOESM12_ESM.pdf]

# Hypothyroidism

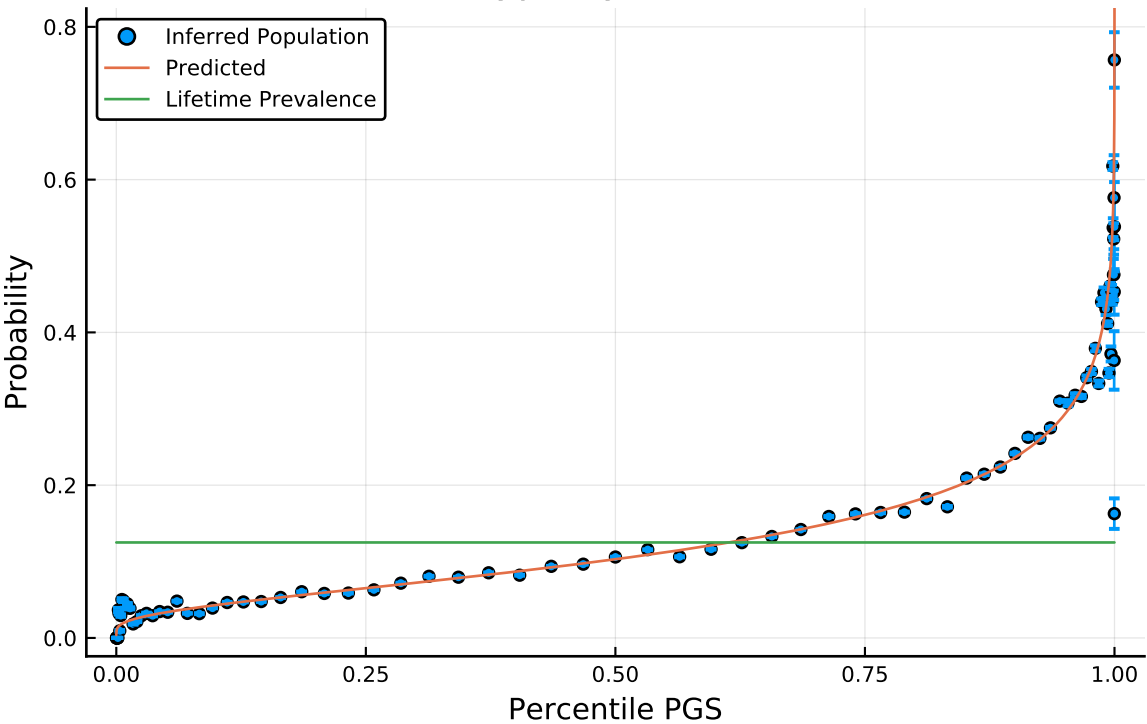

Supplement: Supplementary file 13 — LaTeX Supplementary File [file 41598_2019_51258_MOESM13_ESM.pdf]

# Breast Cancer

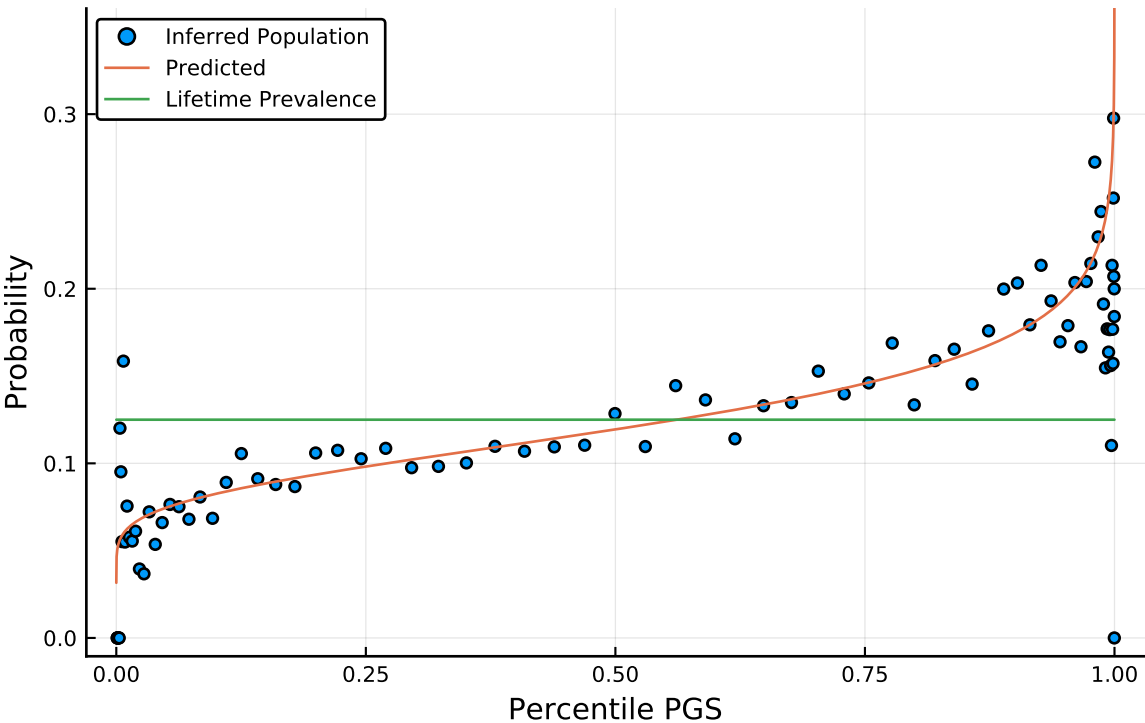

Supplement: Supplementary file 14 — LaTeX Supplementary File [file 41598_2019_51258_MOESM14_ESM.pdf]

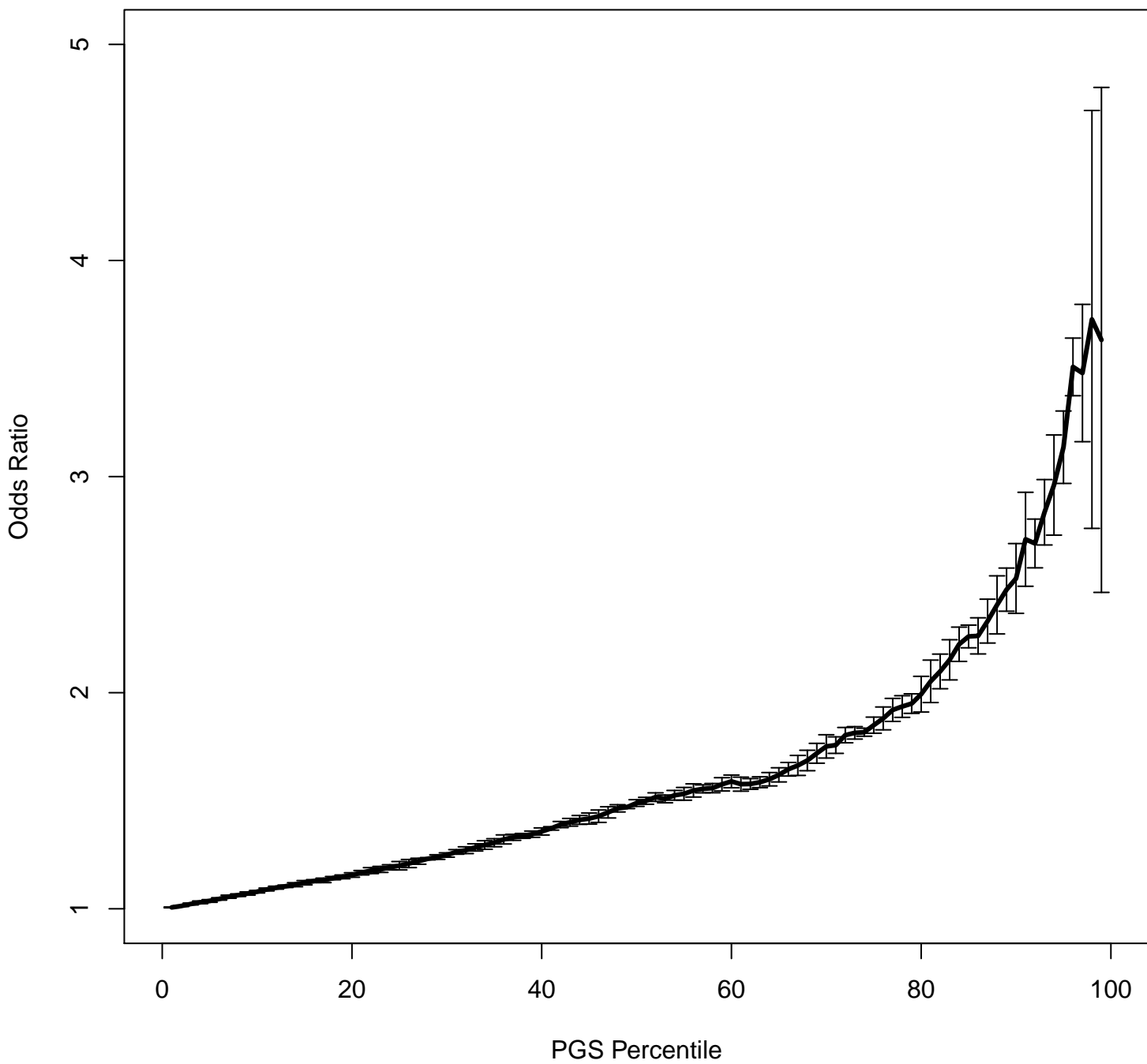

Supplement: Supplementary file 15 — LaTeX Supplementary File [file 41598_2019_51258_MOESM15_ESM.pdf]

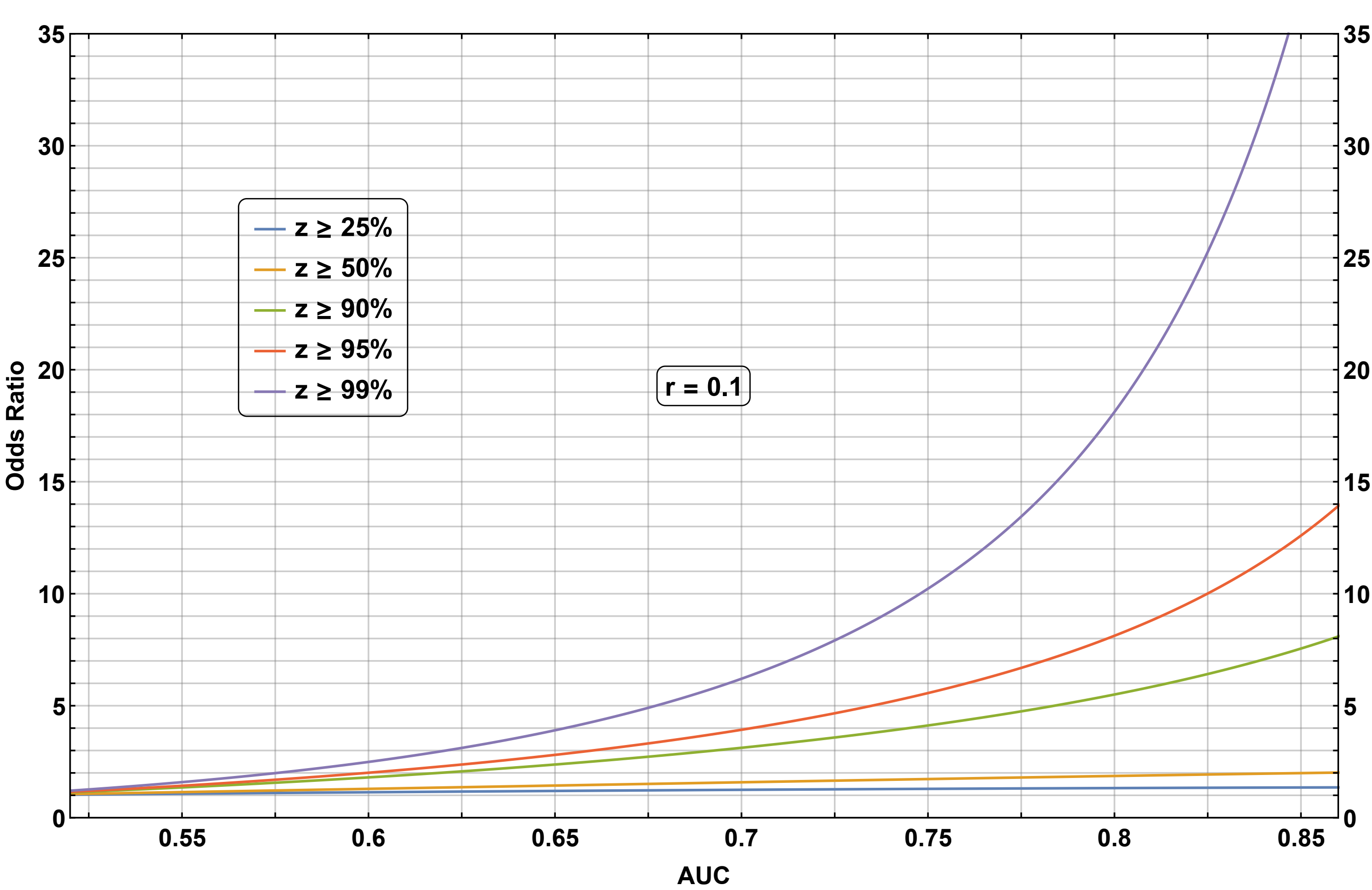

Supplement: Supplementary file 16 — LaTeX Supplementary File [file 41598_2019_51258_MOESM16_ESM.pdf]

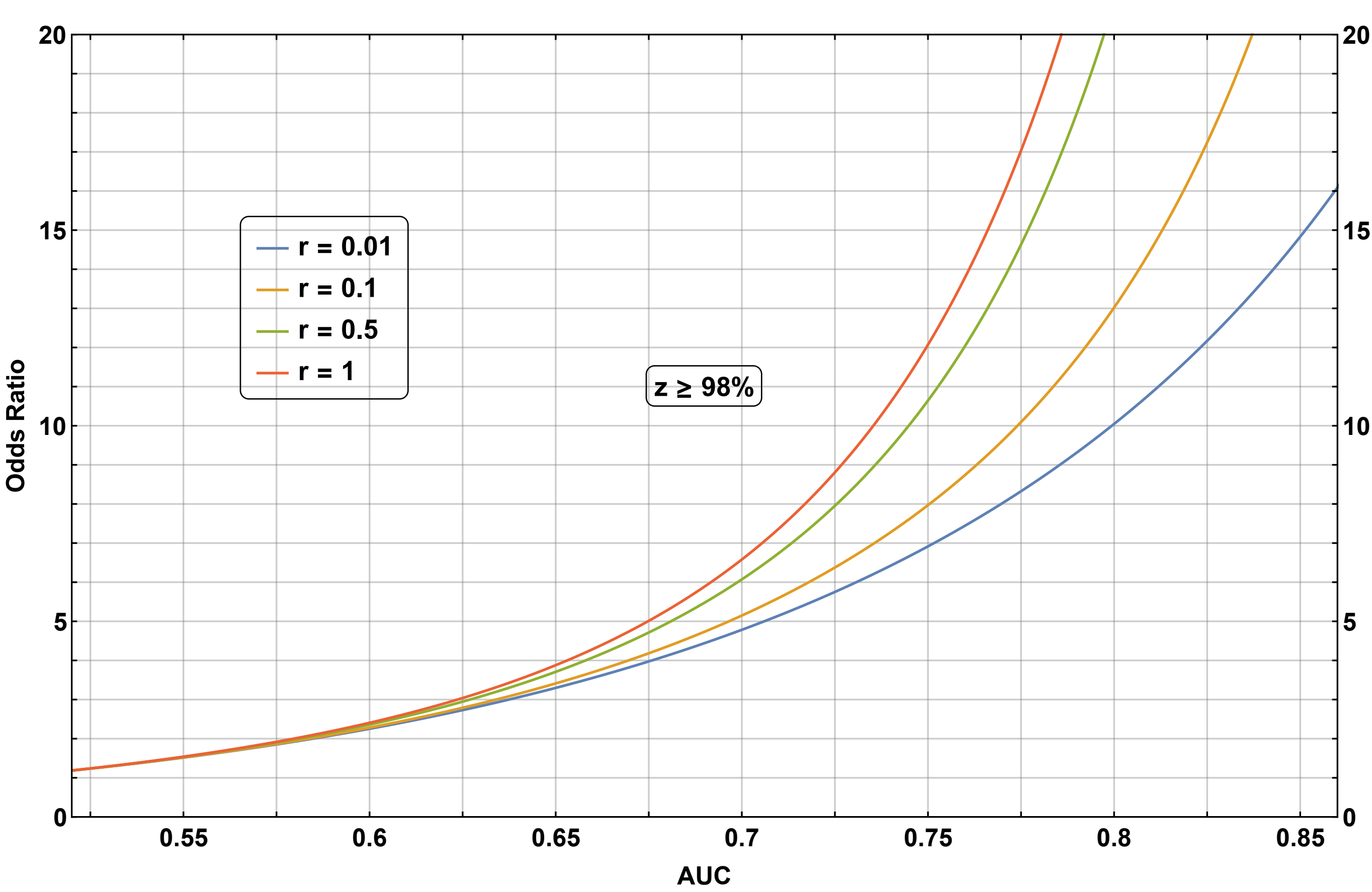

Supplement: Supplementary file 17 — LaTeX Supplementary File [file 41598_2019_51258_MOESM17_ESM.pdf]

Odds Ratio

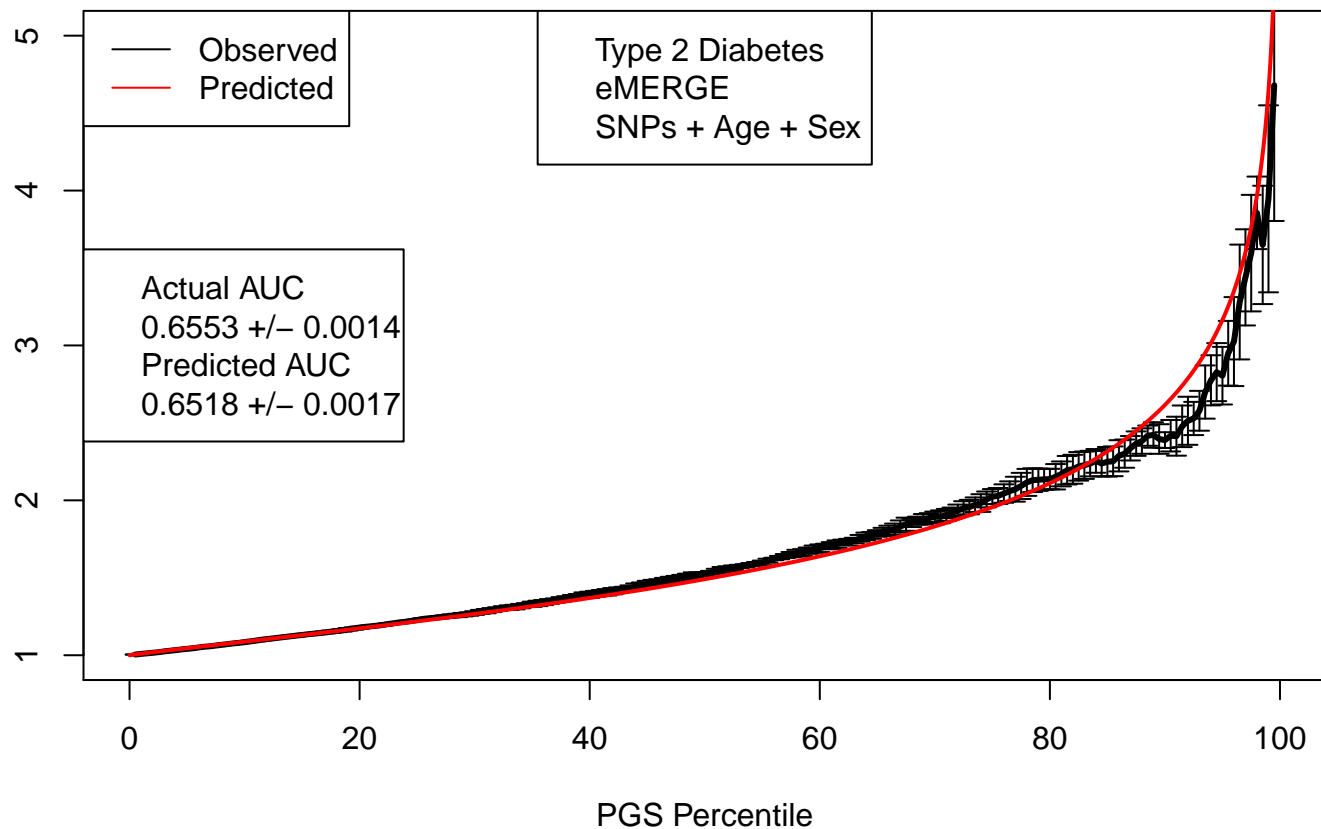

Supplement: Supplementary file 18 — LaTeX Supplementary File [file 41598_2019_51258_MOESM18_ESM.pdf]

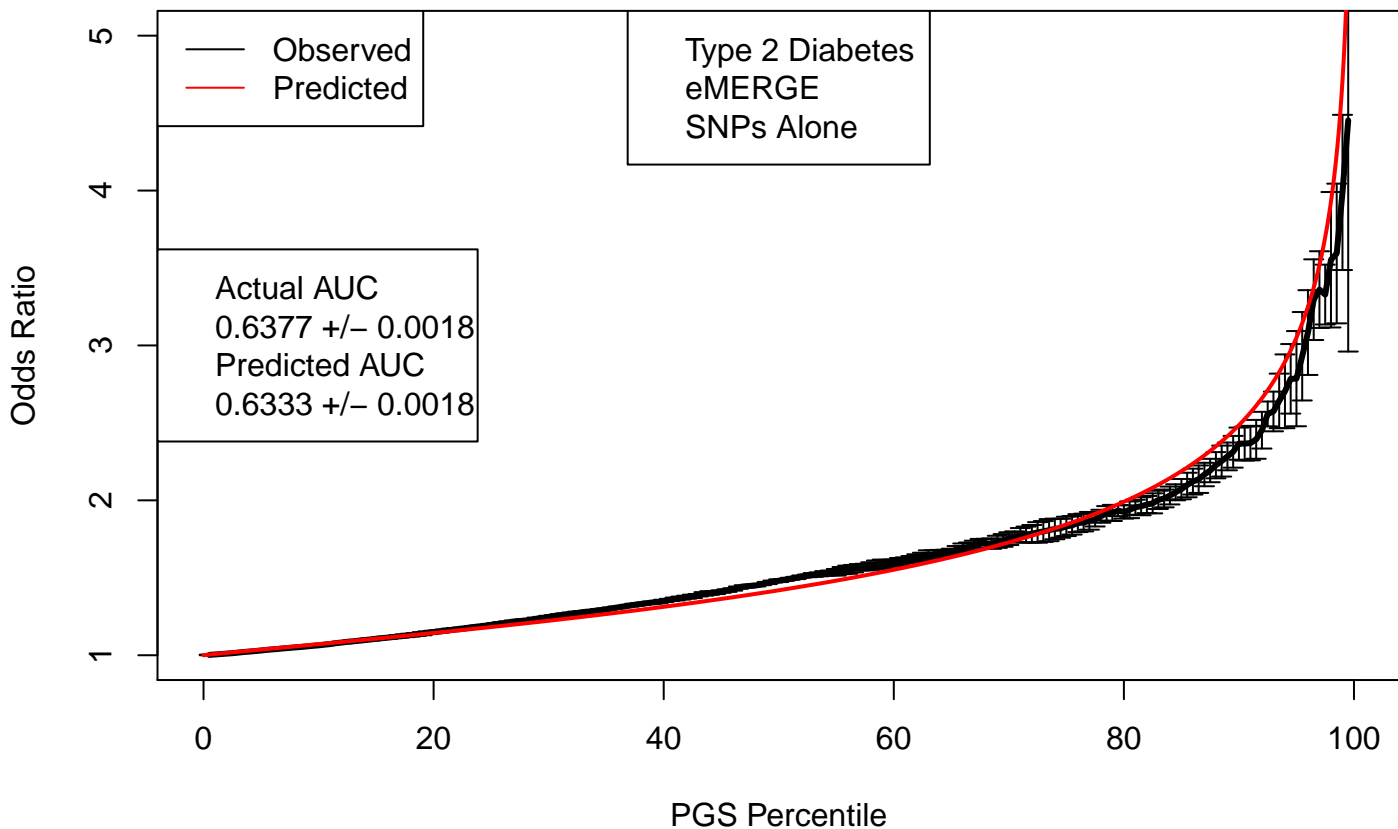

Supplement: Supplementary file 19 — LaTeX Supplementary File [file 41598_2019_51258_MOESM19_ESM.pdf]

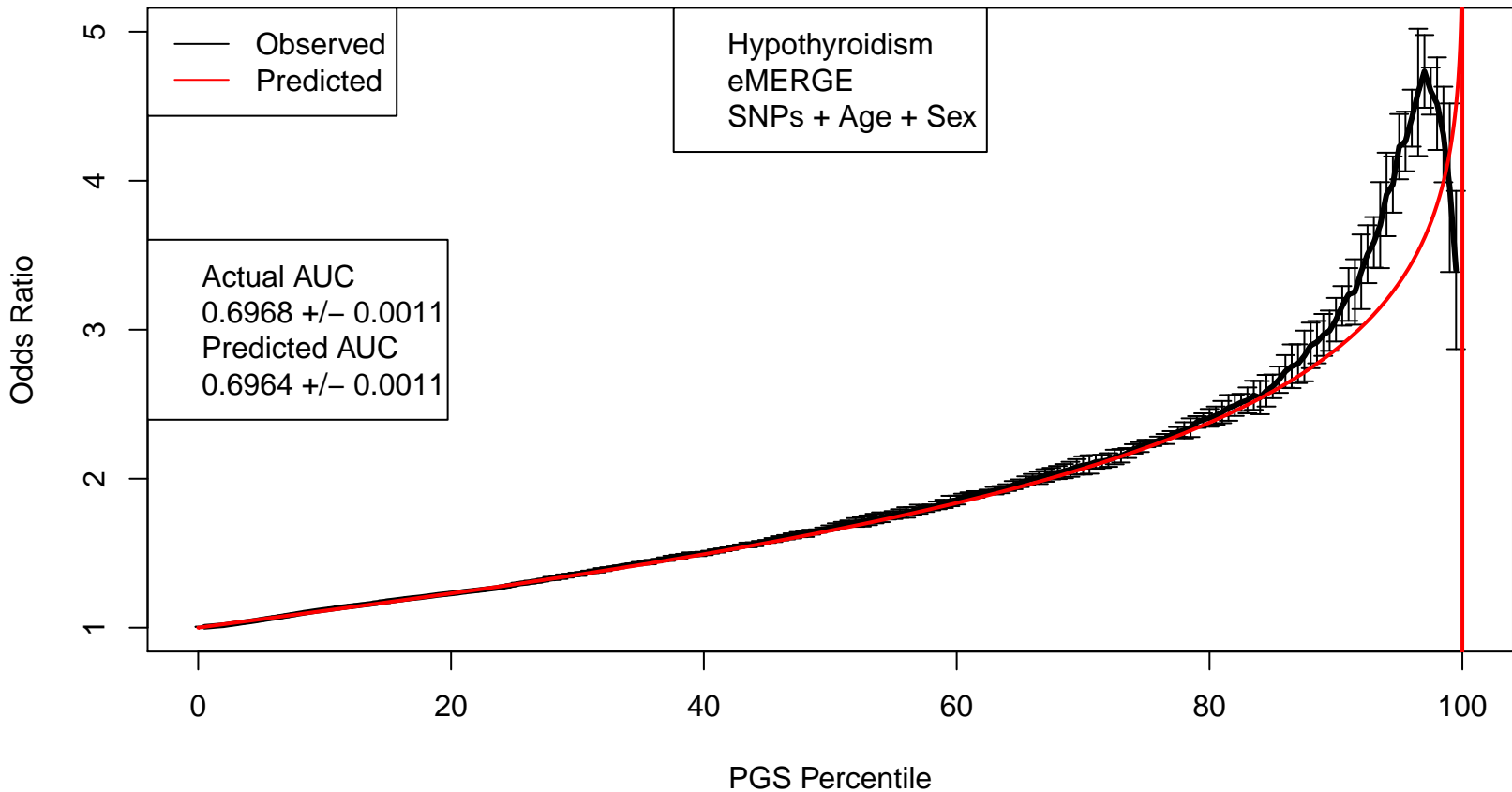

Supplement: Supplementary file 20 — LaTeX Supplementary File [file 41598_2019_51258_MOESM20_ESM.pdf]

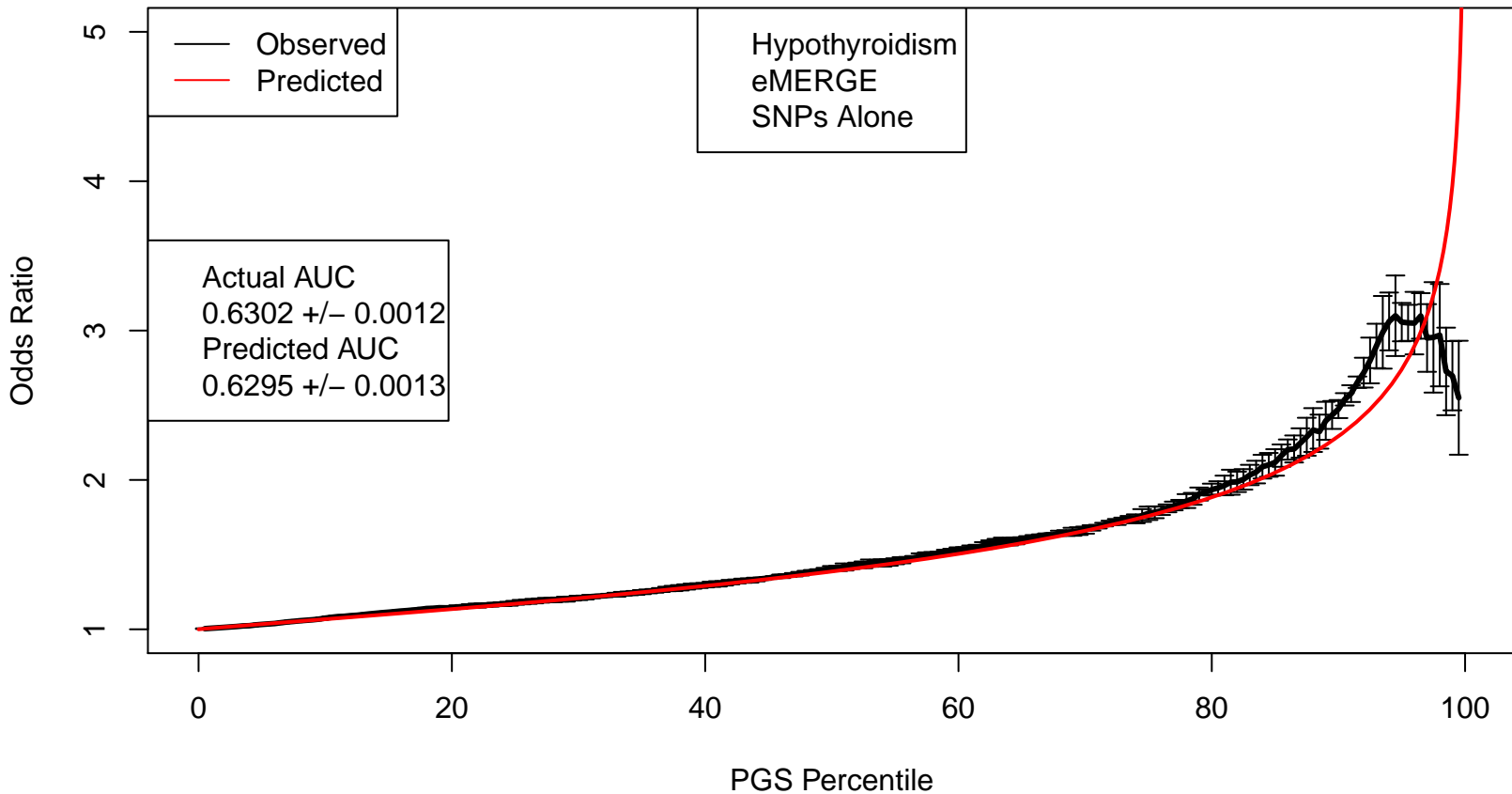

Supplement: Supplementary file 21 — LaTeX Supplementary File [file 41598_2019_51258_MOESM21_ESM.pdf]

Odds Ratio

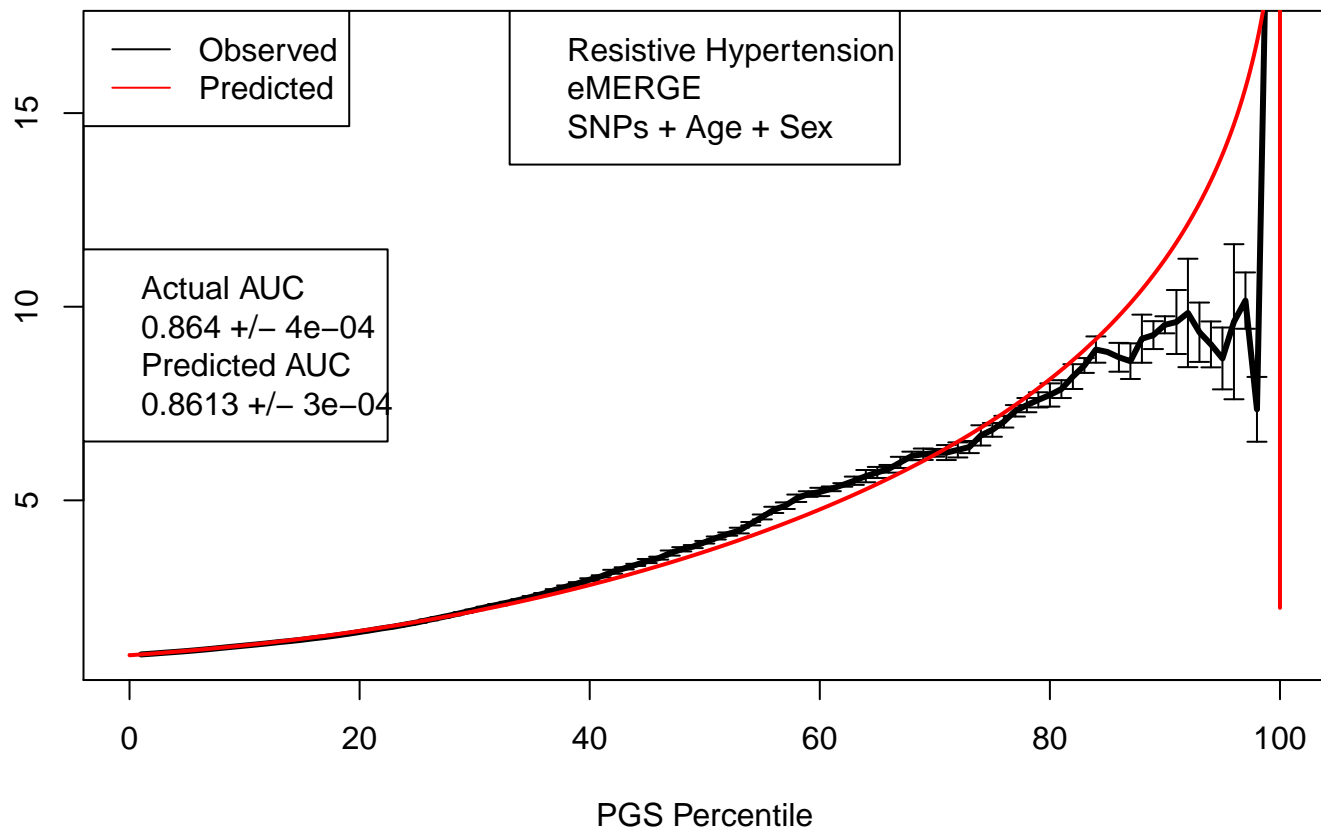

Supplement: Supplementary file 22 — LaTeX Supplementary File [file 41598_2019_51258_MOESM22_ESM.pdf]

Odds Ratio

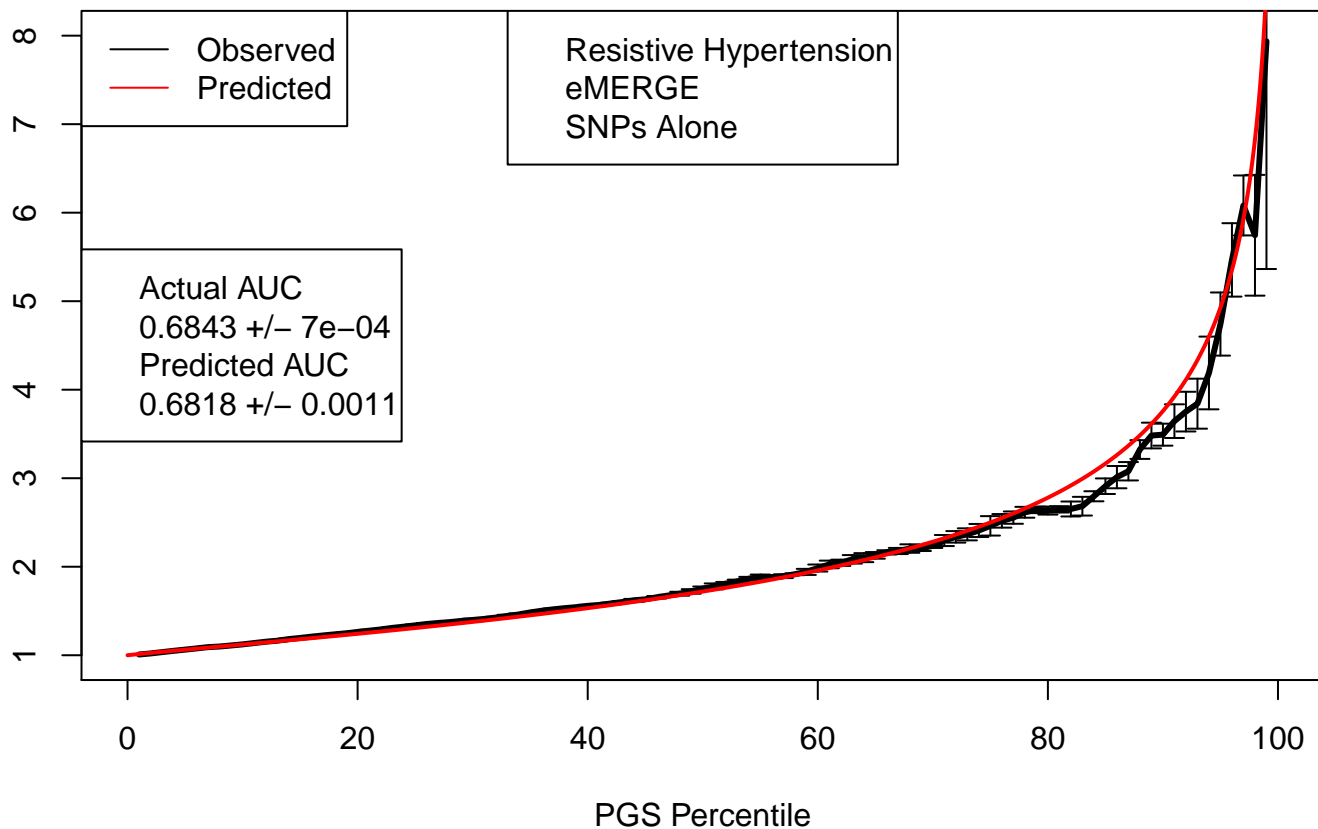

Supplement: Supplementary file 23 — LaTeX Supplementary File [file 41598_2019_51258_MOESM23_ESM.pdf]

Odds Ratio

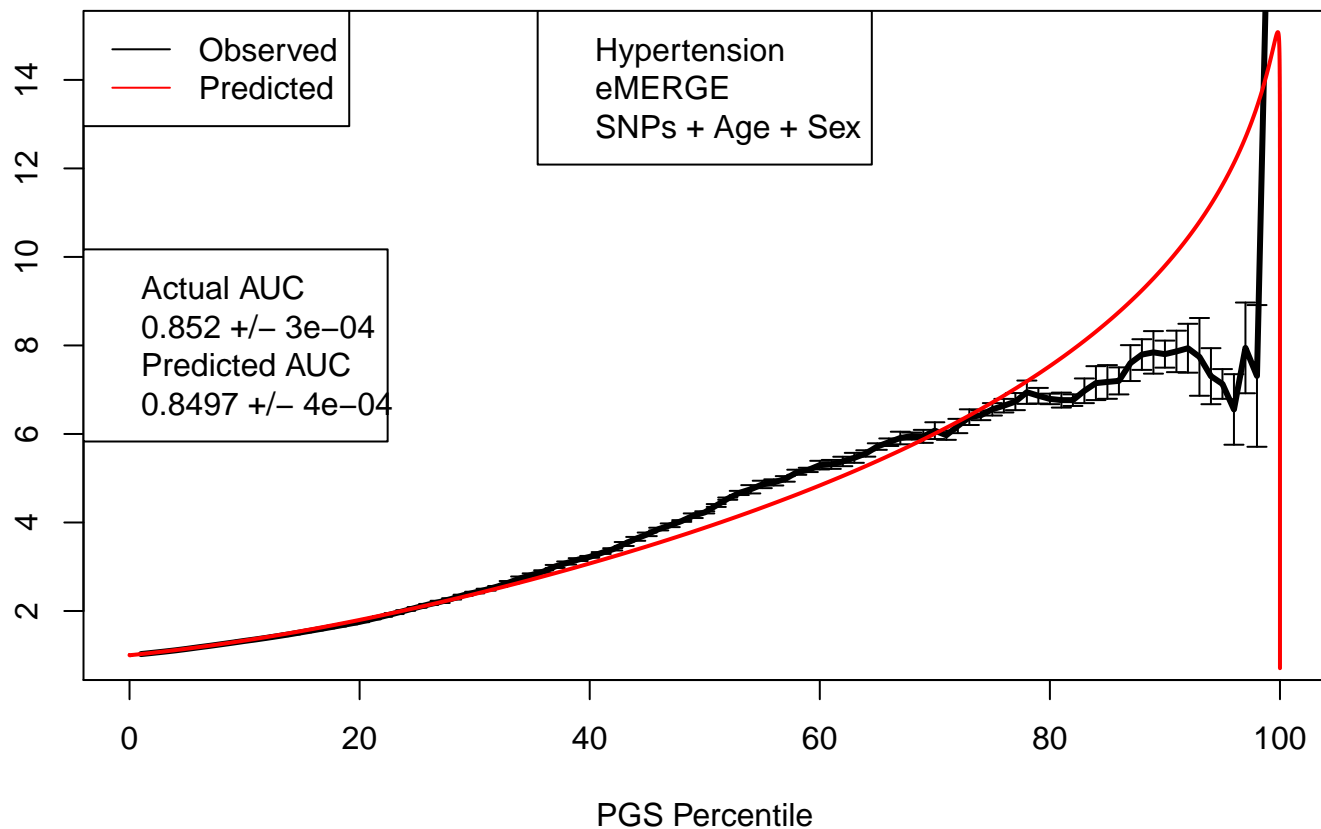

Supplement: Supplementary file 24 — LaTeX Supplementary File [file 41598_2019_51258_MOESM24_ESM.pdf]

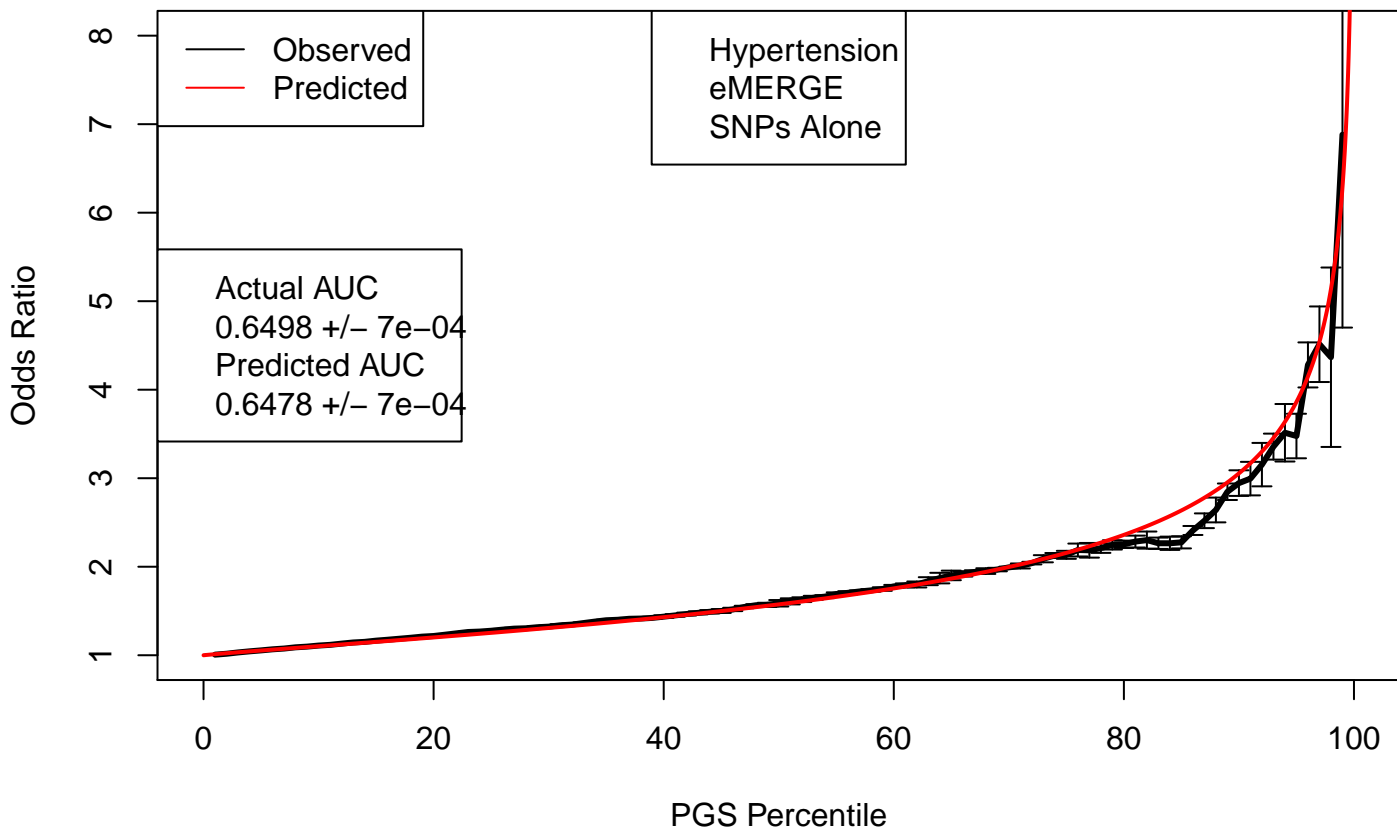

Supplement: Supplementary file 25 — LaTeX Supplementary File [file 41598_2019_51258_MOESM25_ESM.pdf]

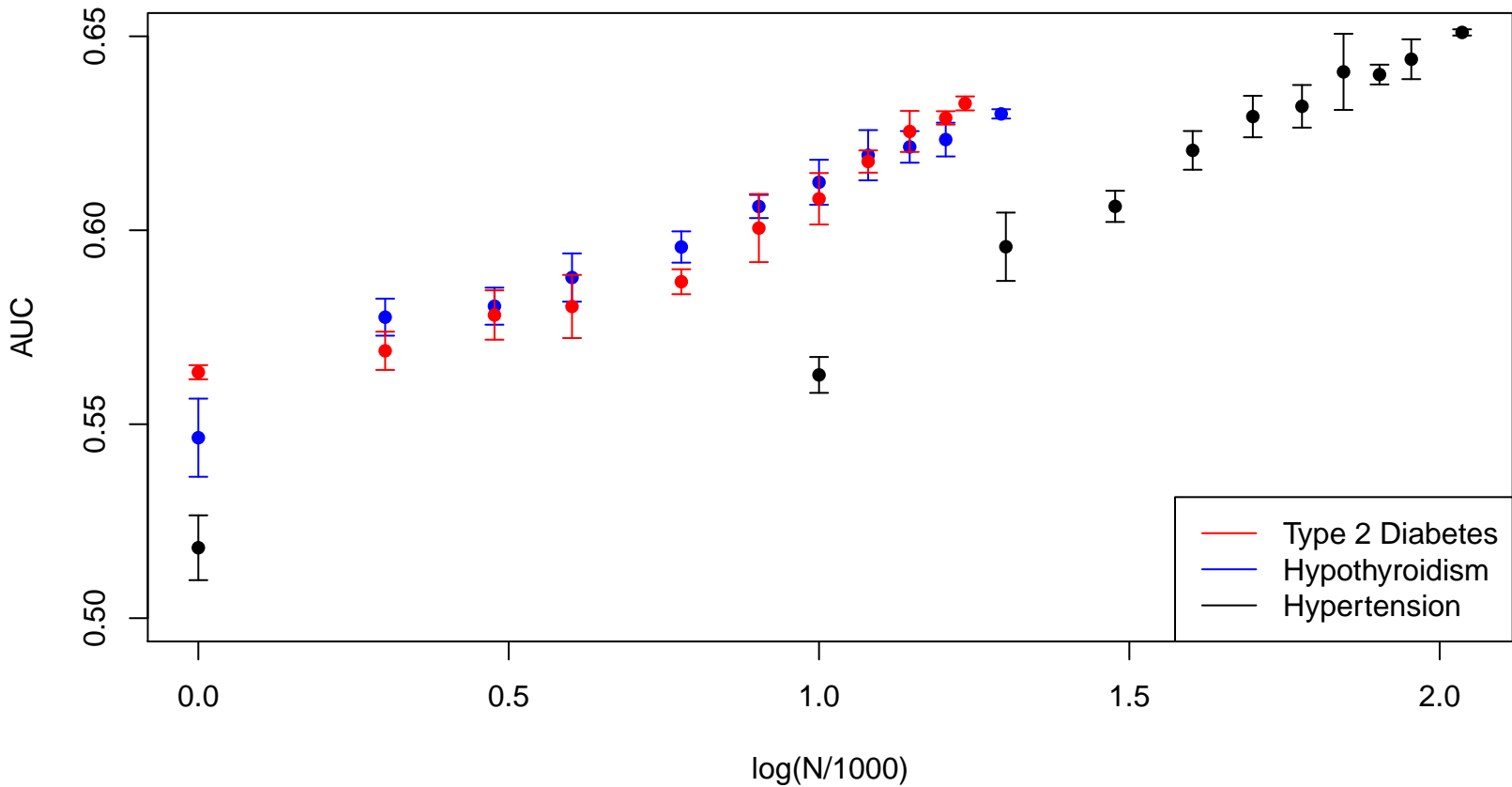

Supplement: Supplementary file 26 — LaTeX Supplementary File [file 41598_2019_51258_MOESM26_ESM.pdf]

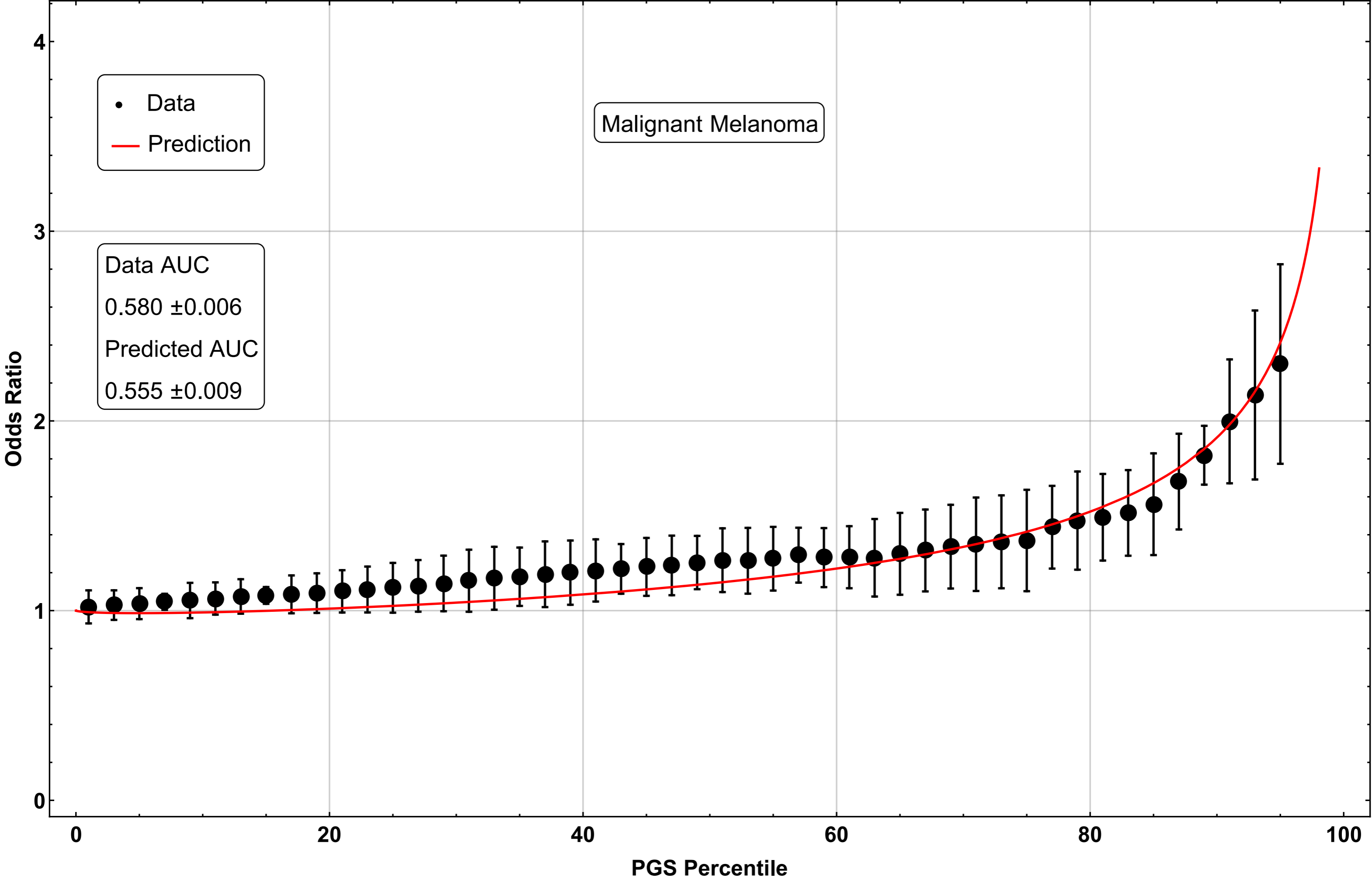

Supplement: Supplementary file 27 — LaTeX Supplementary File [file 41598_2019_51258_MOESM27_ESM.pdf]

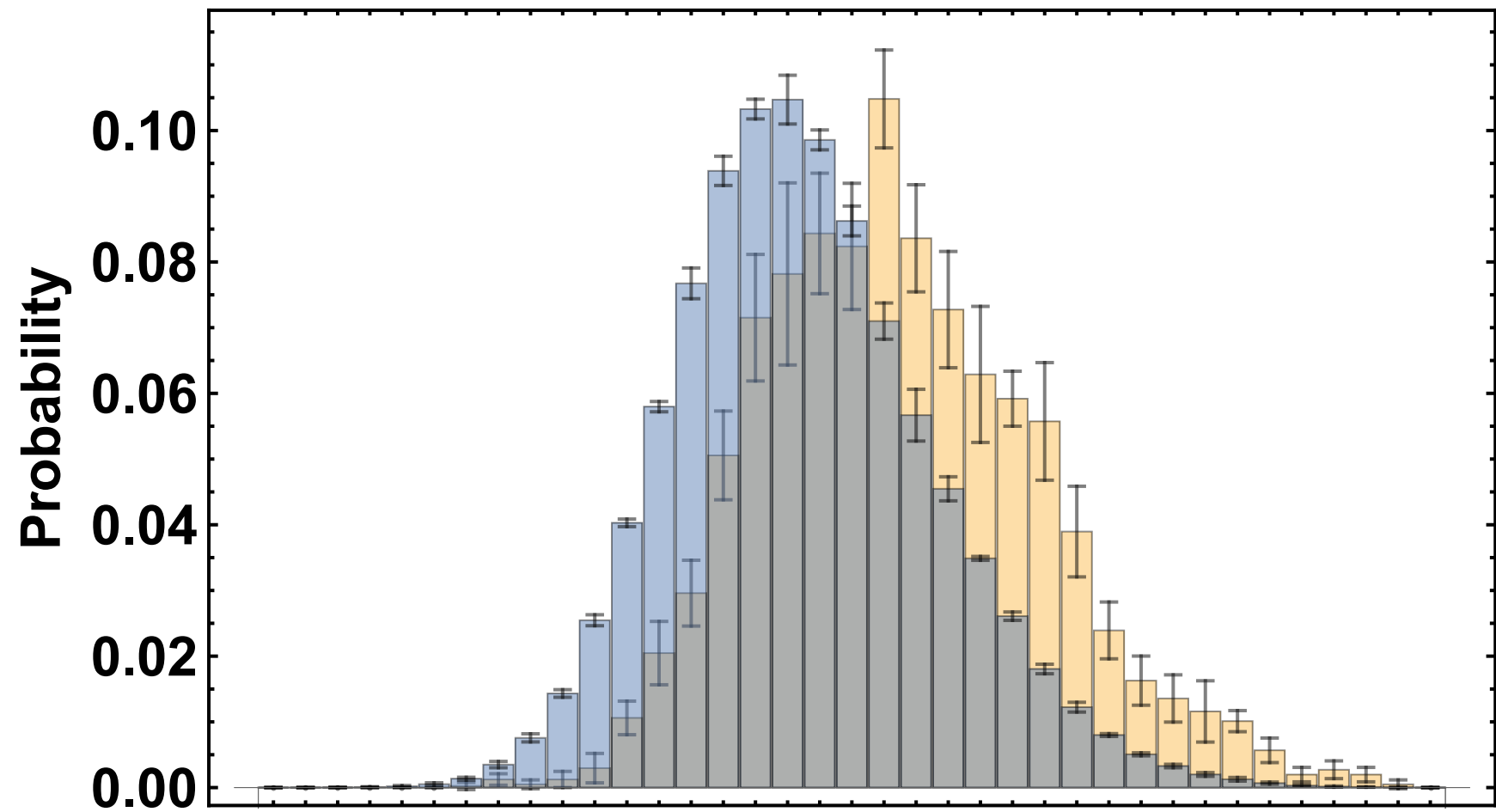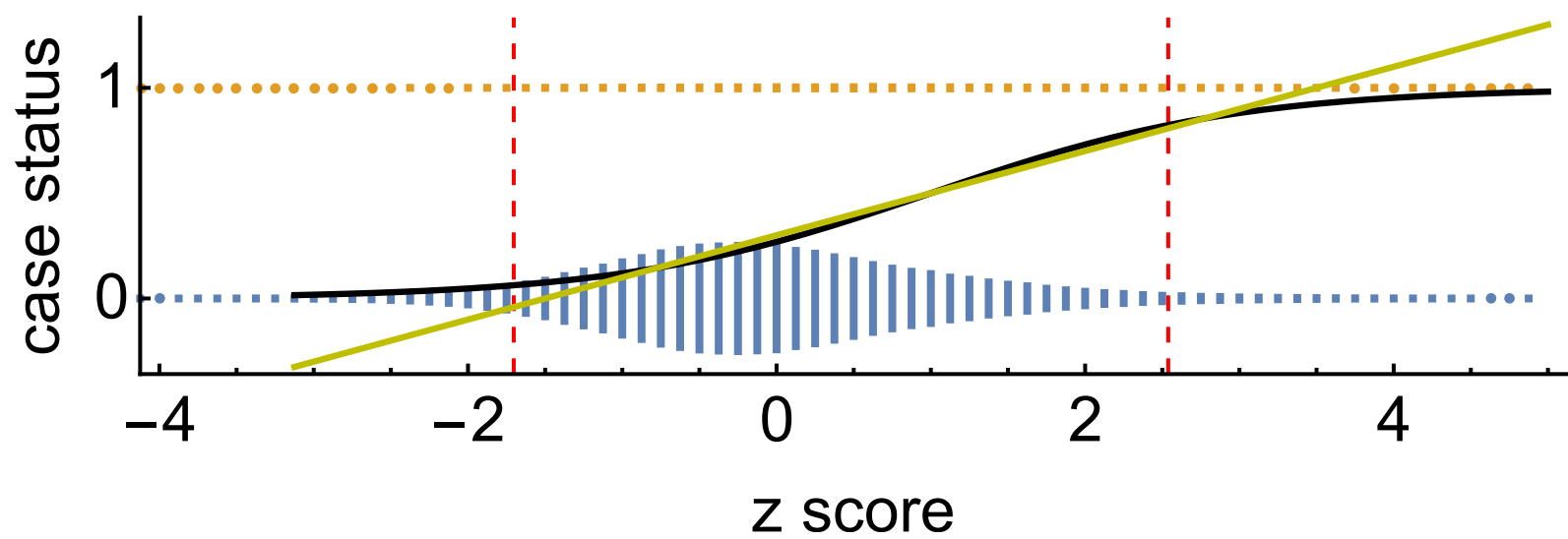

Supplement: Supplementary file 28 — LaTeX Supplementary File [file 41598_2019_51258_MOESM28_ESM.pdf]

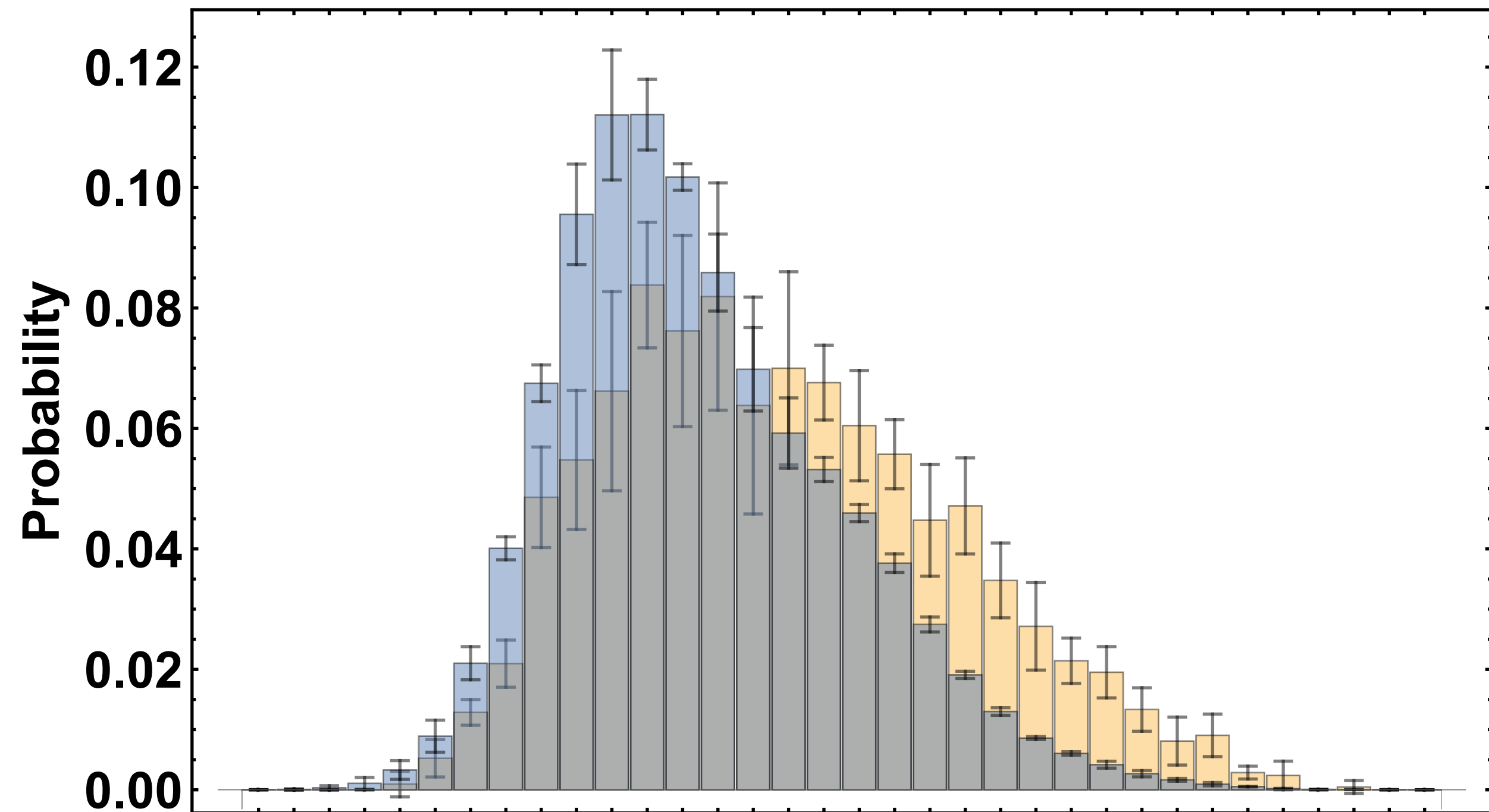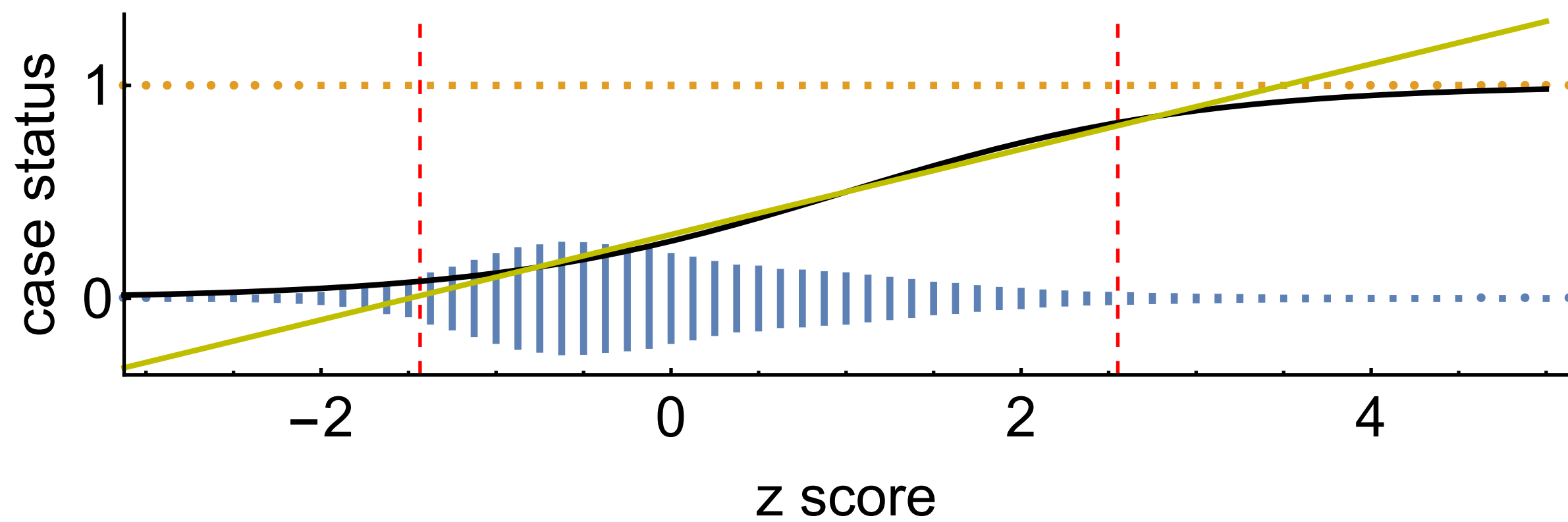

Supplement: Supplementary file 29 — LaTeX Supplementary File [file 41598_2019_51258_MOESM29_ESM.pdf]

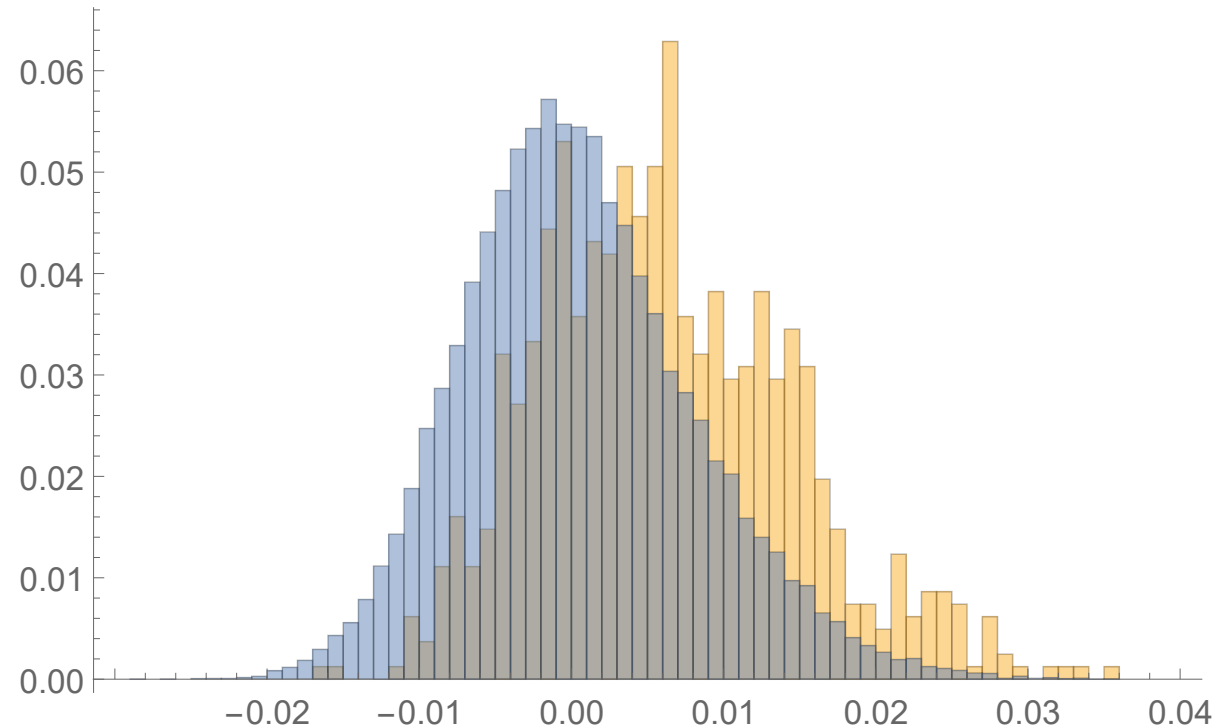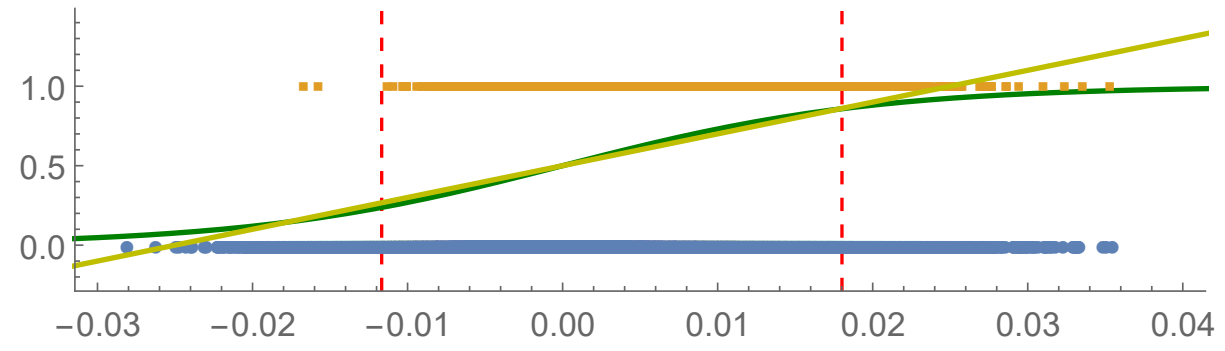

Supplement: Supplementary file 30 — LaTeX Supplementary File [file 41598_2019_51258_MOESM30_ESM.pdf]

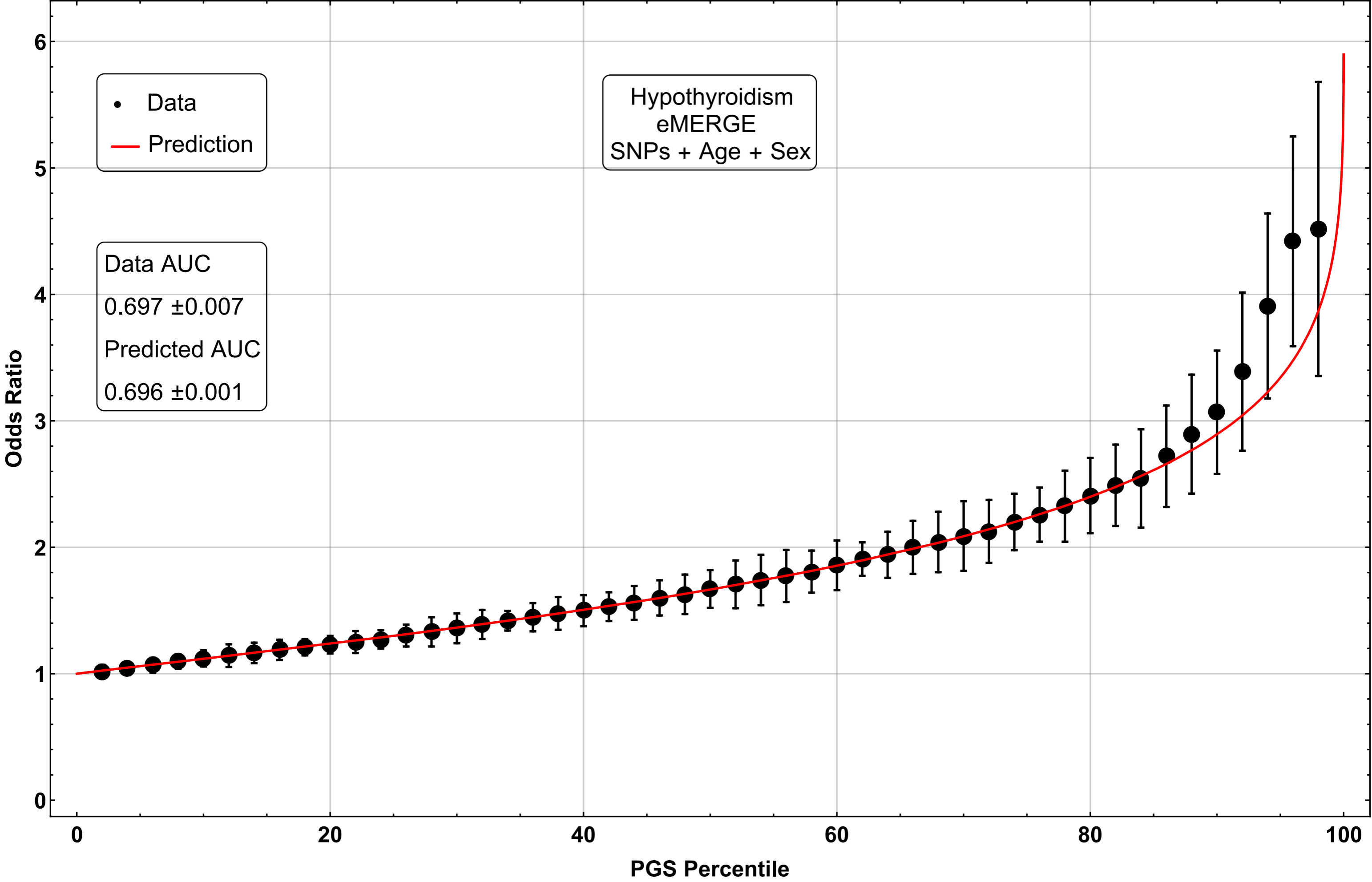

Supplement: Supplementary file 31 — LaTeX Supplementary File [file 41598_2019_51258_MOESM31_ESM.pdf]

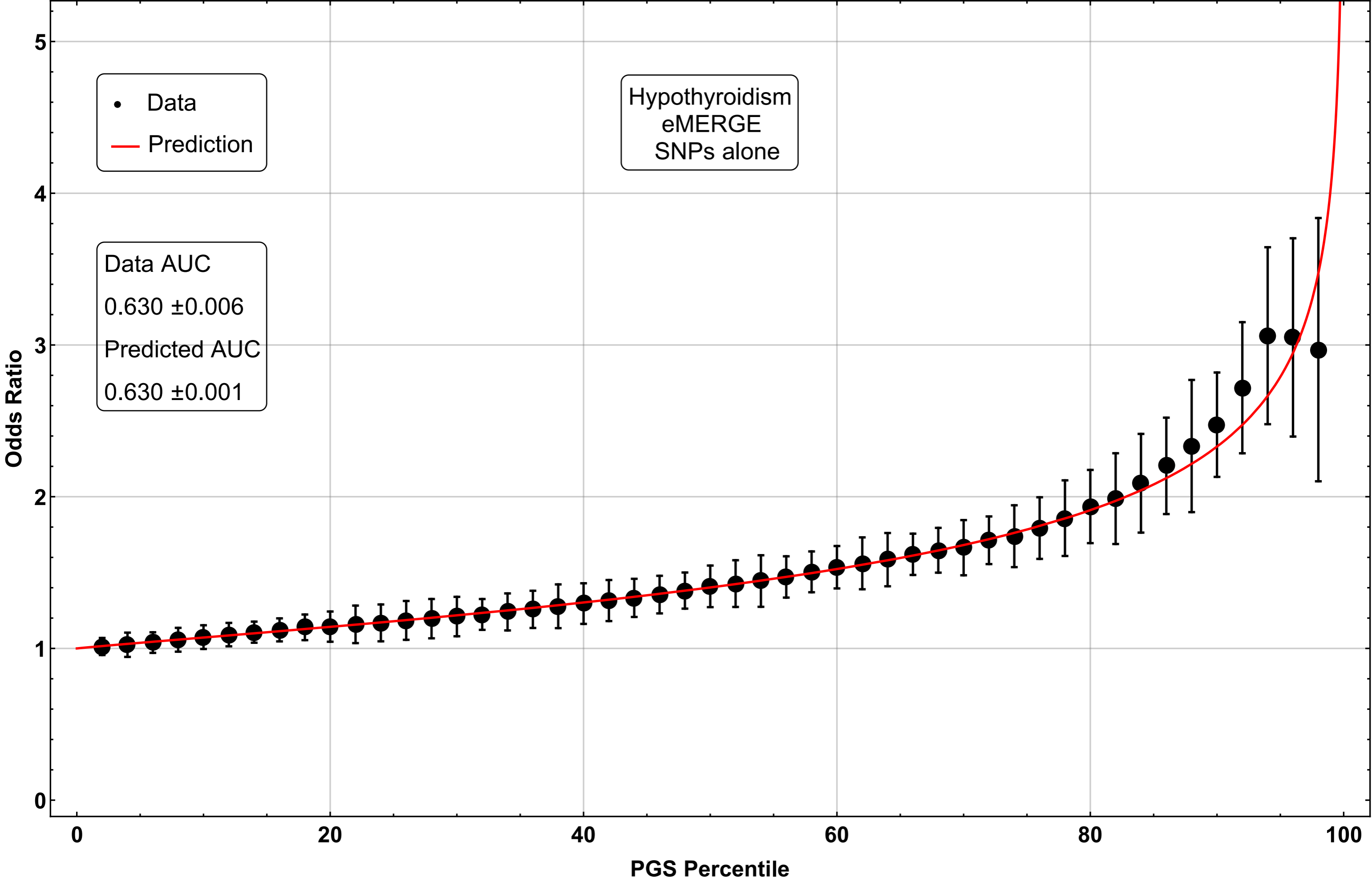

Supplement: Supplementary file 32 — LaTeX Supplementary File [file 41598_2019_51258_MOESM32_ESM.pdf]

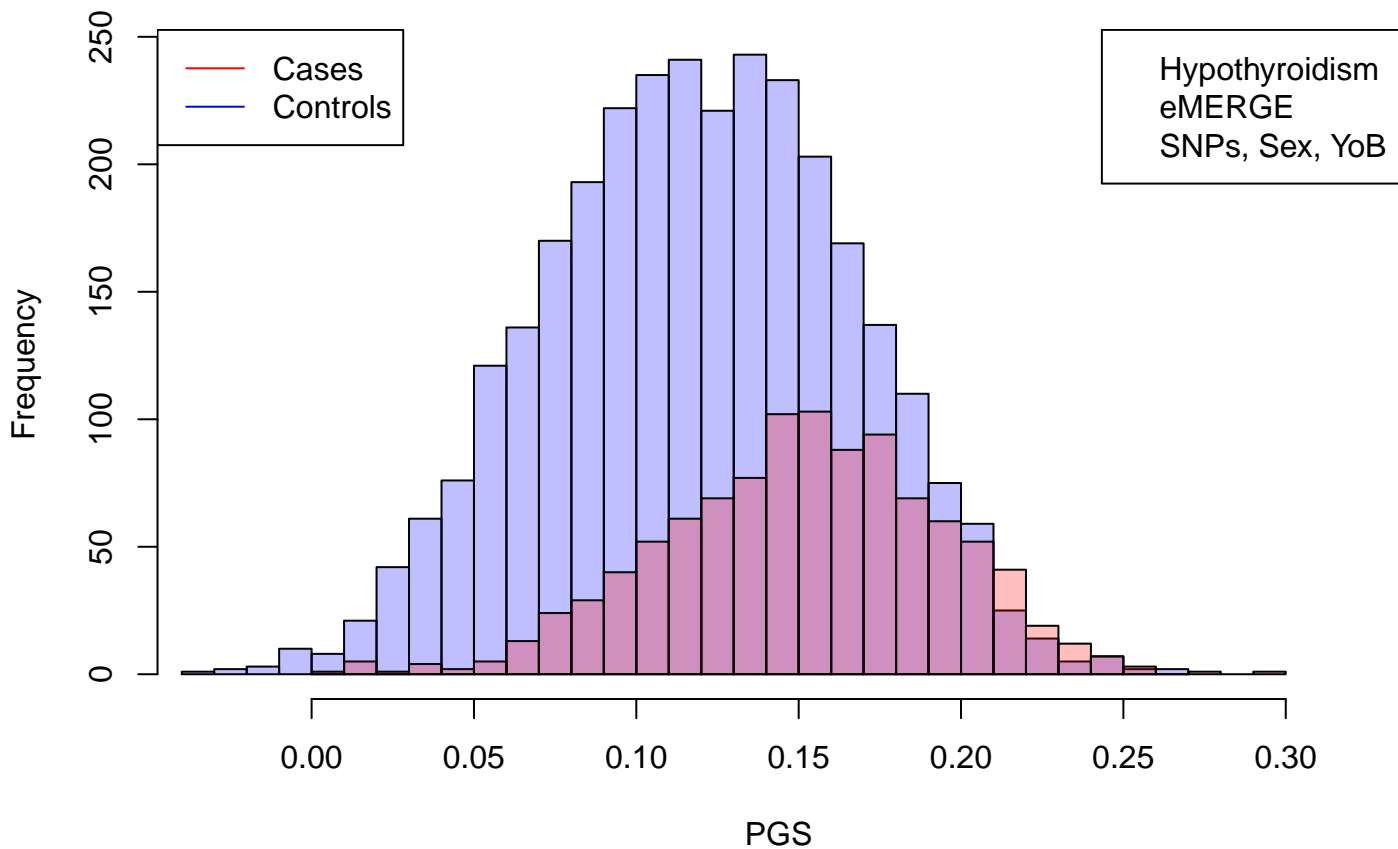

Supplement: Supplementary file 33 — LaTeX Supplementary File [file 41598_2019_51258_MOESM33_ESM.pdf]

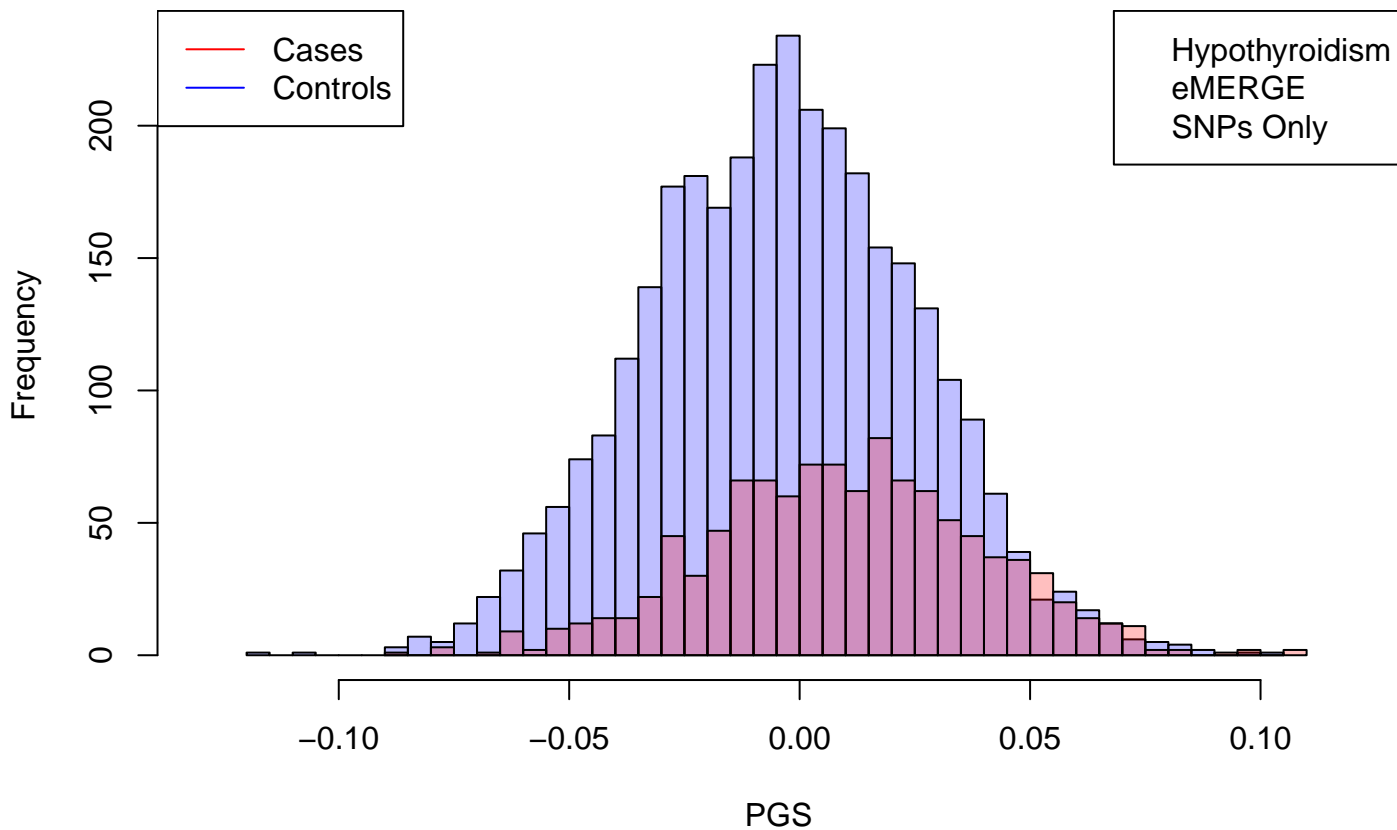

Supplement: Supplementary file 34 — LaTeX Supplementary File [file 41598_2019_51258_MOESM34_ESM.pdf]

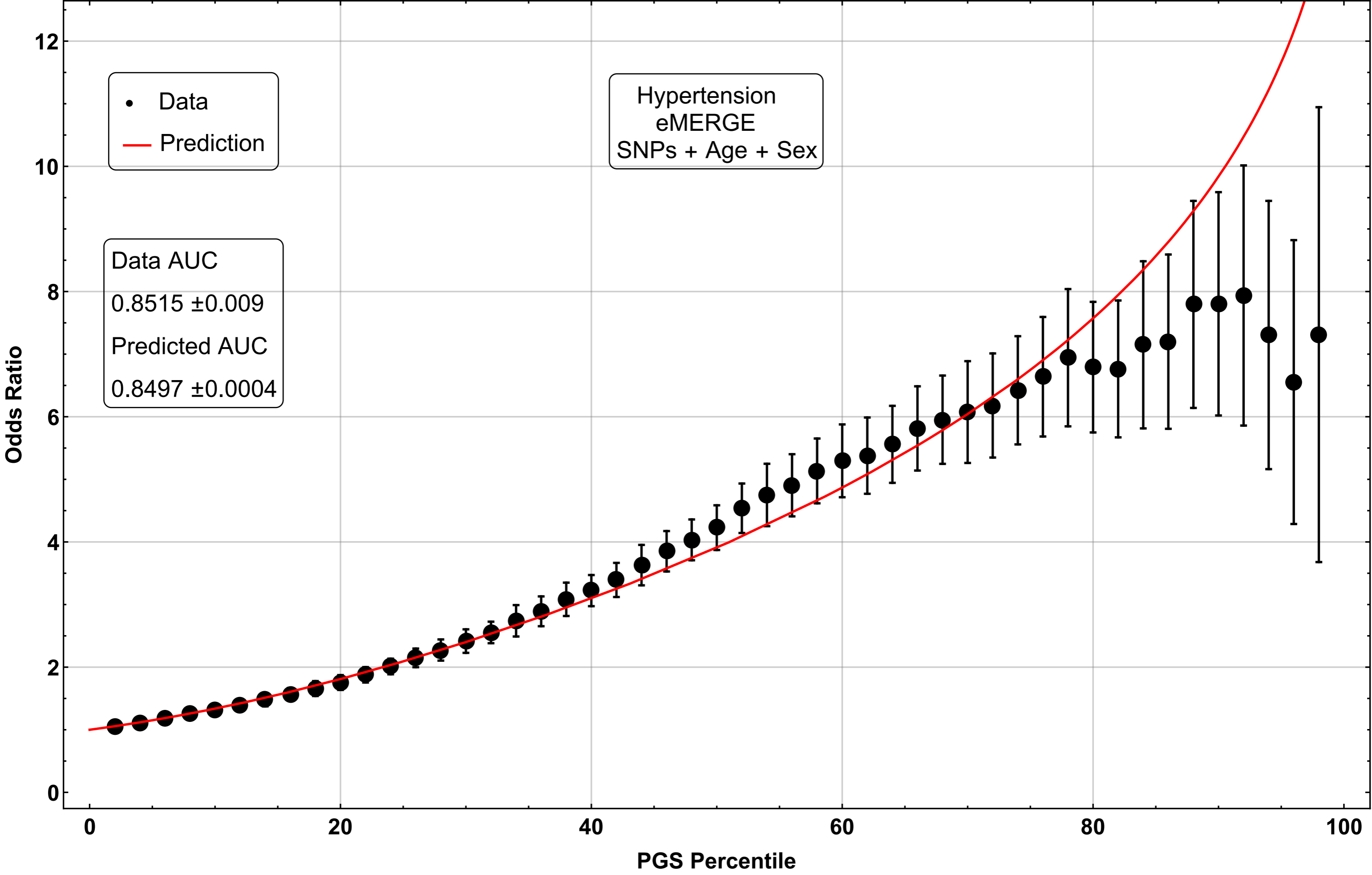

Supplement: Supplementary file 35 — LaTeX Supplementary File [file 41598_2019_51258_MOESM35_ESM.pdf]

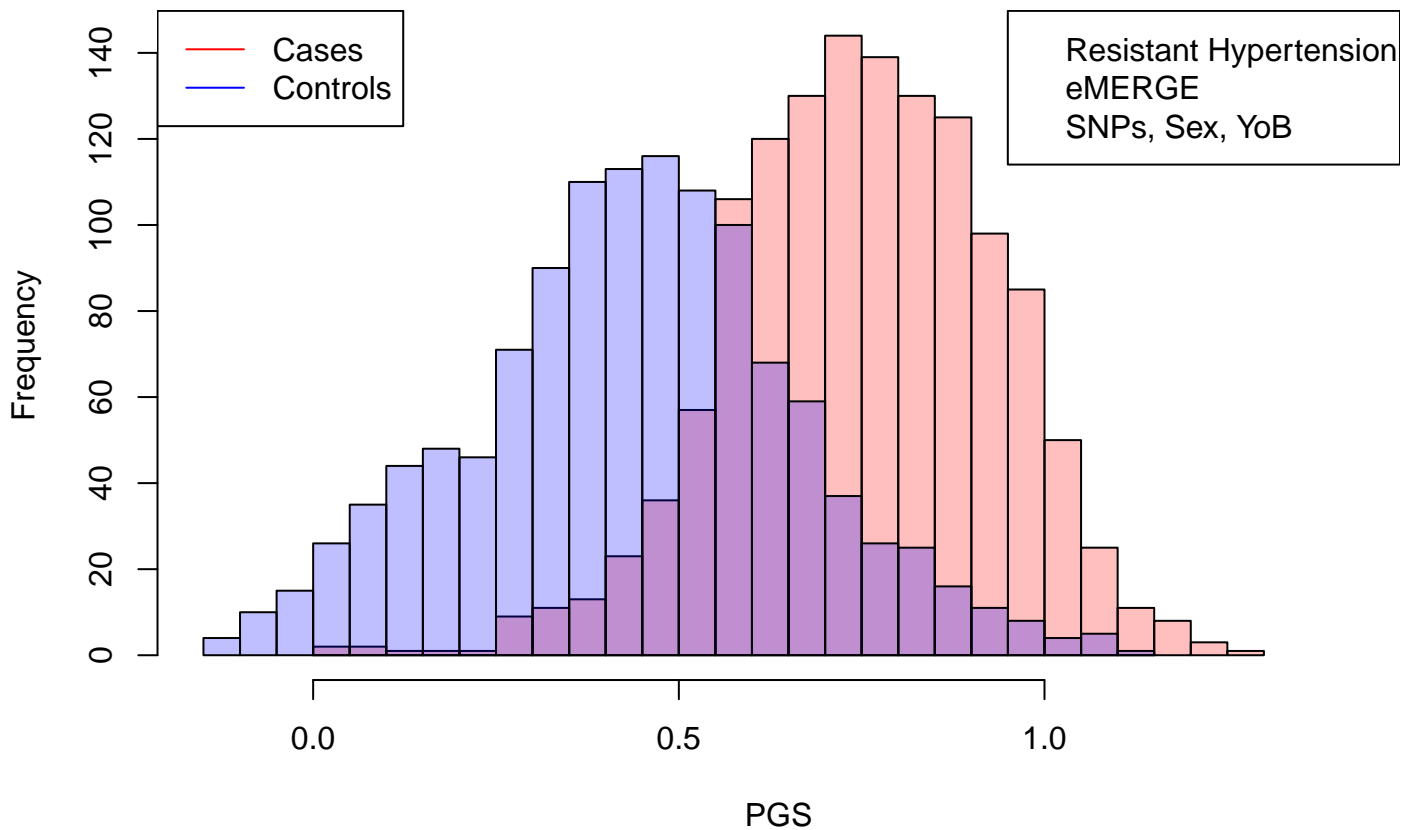

Supplement: Supplementary file 36 — LaTeX Supplementary File [file 41598_2019_51258_MOESM36_ESM.pdf]

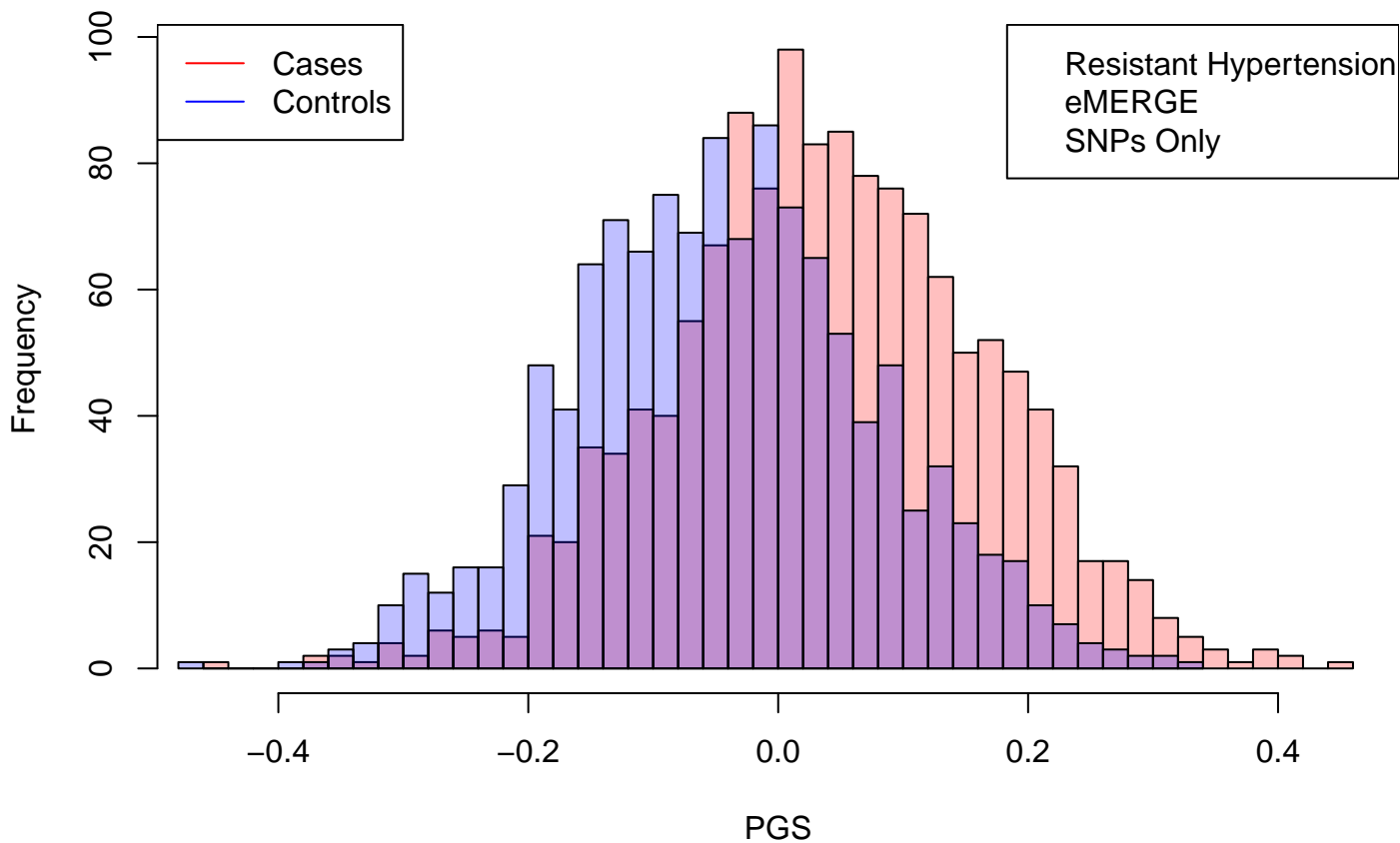

Supplement: Supplementary file 37 — LaTeX Supplementary File [file 41598_2019_51258_MOESM37_ESM.pdf]

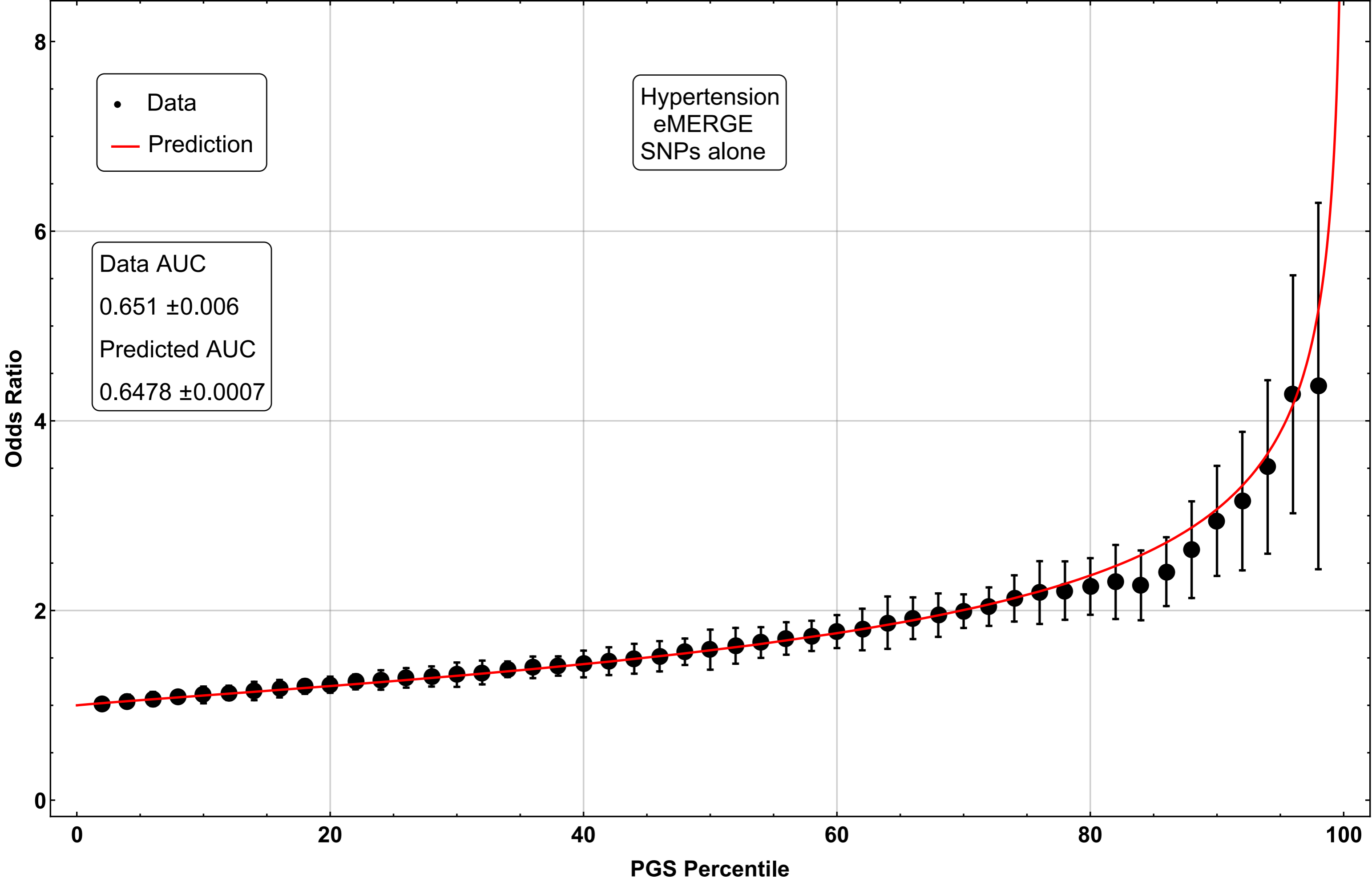

Supplement: Supplementary file 38 — LaTeX Supplementary File [file 41598_2019_51258_MOESM38_ESM.pdf]

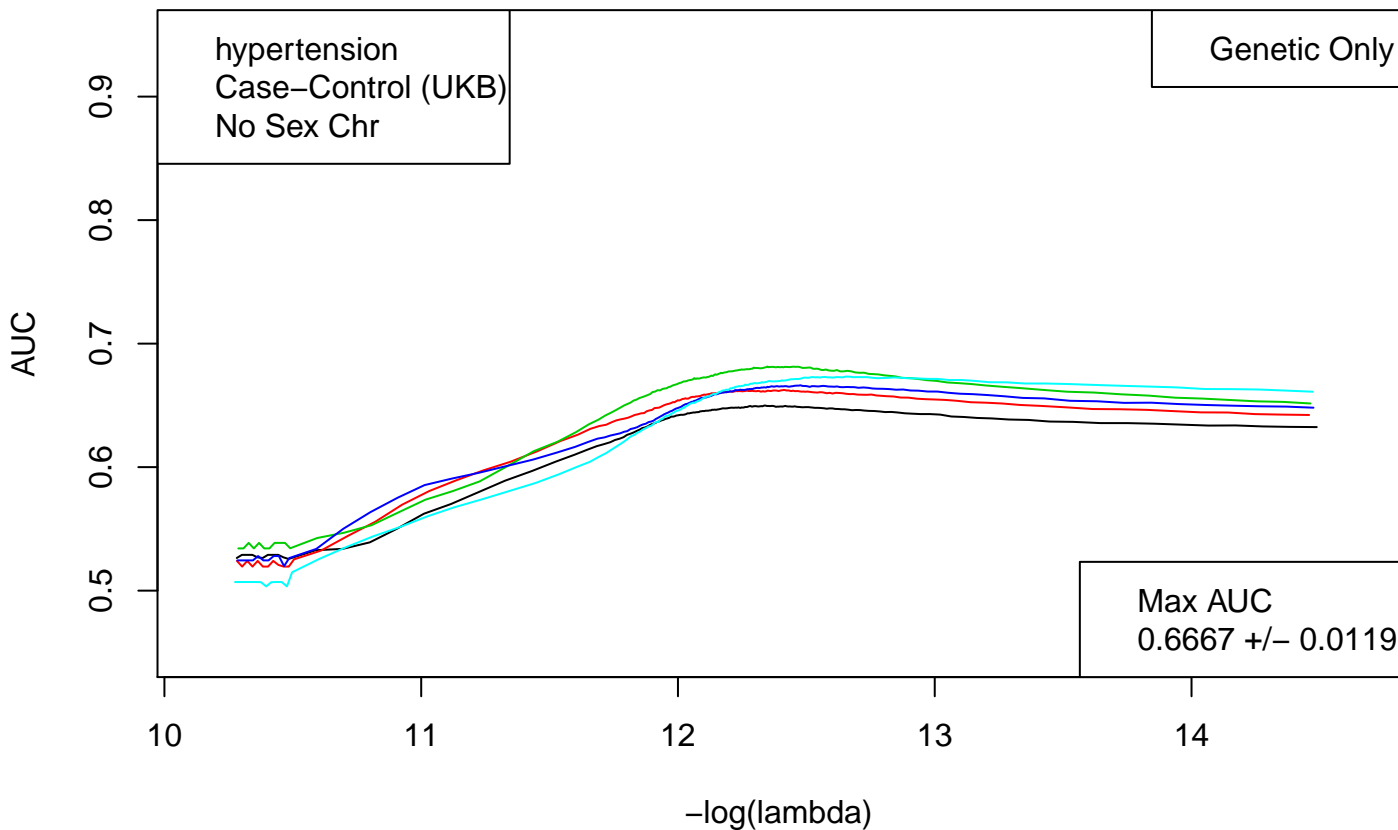

Supplement: Supplementary file 39 — LaTeX Supplementary File [file 41598_2019_51258_MOESM39_ESM.pdf]

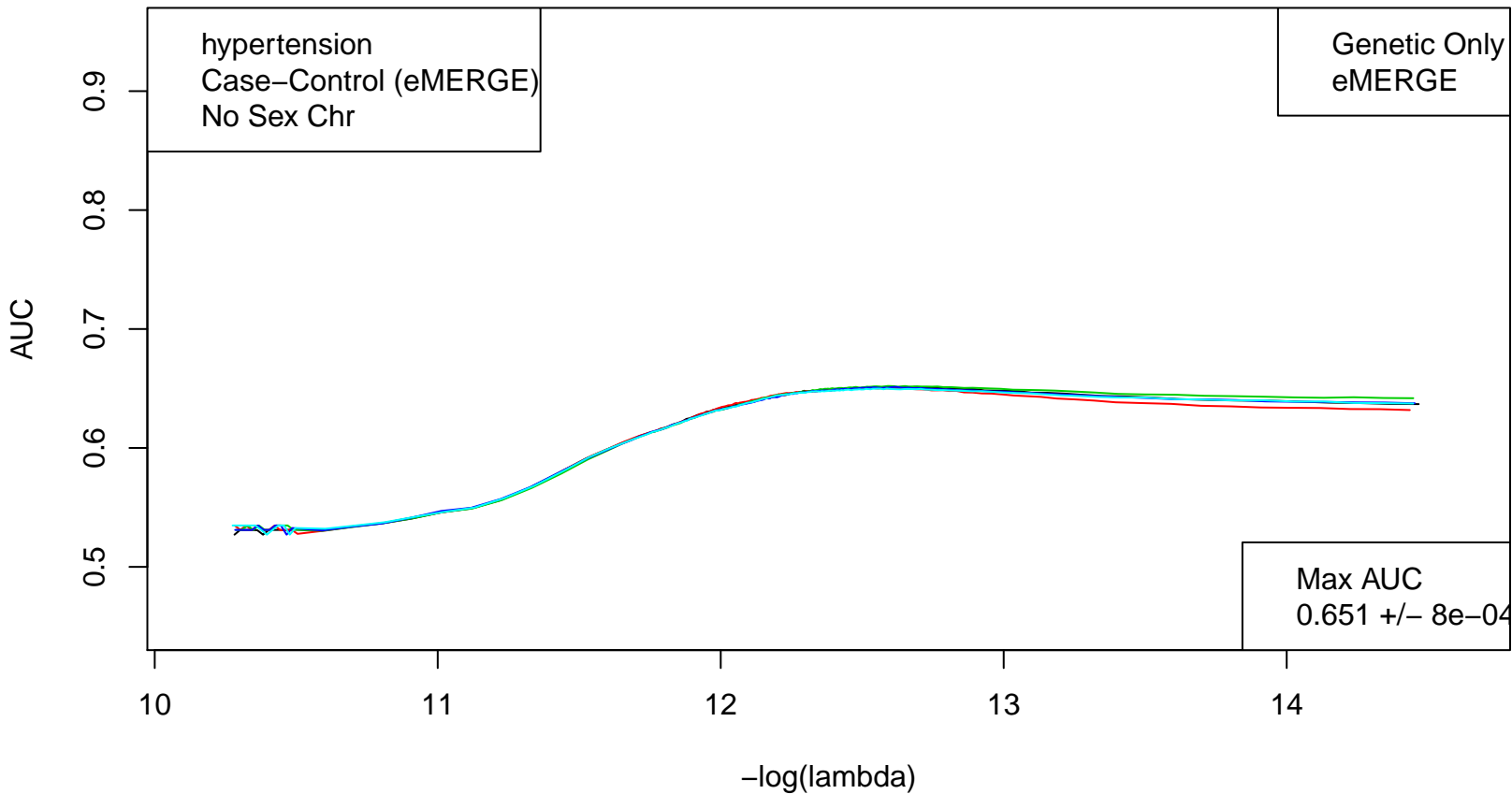

Supplement: Supplementary file 40 — LaTeX Supplementary File [file 41598_2019_51258_MOESM40_ESM.pdf]

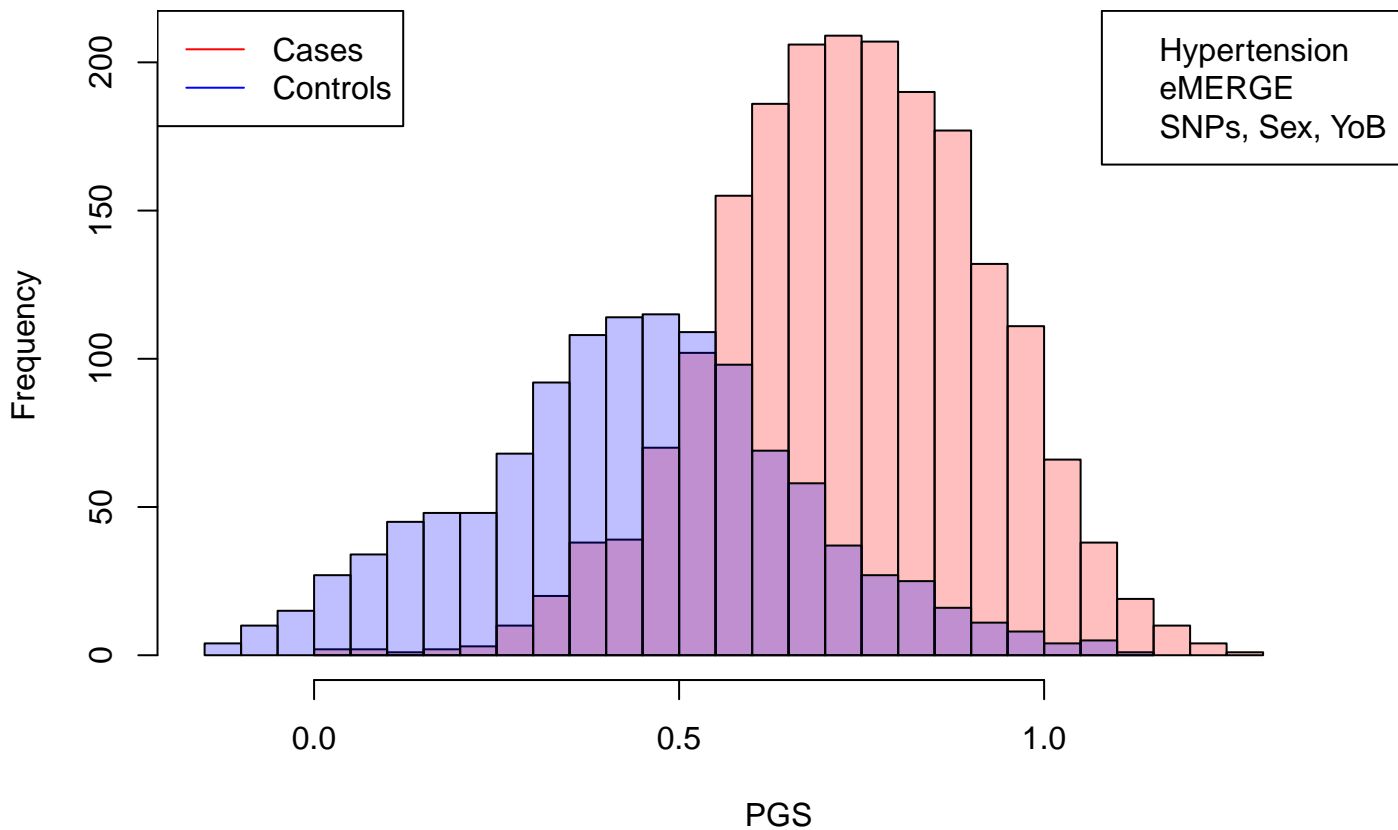

Supplement: Supplementary file 41 — LaTeX Supplementary File [file 41598_2019_51258_MOESM41_ESM.pdf]

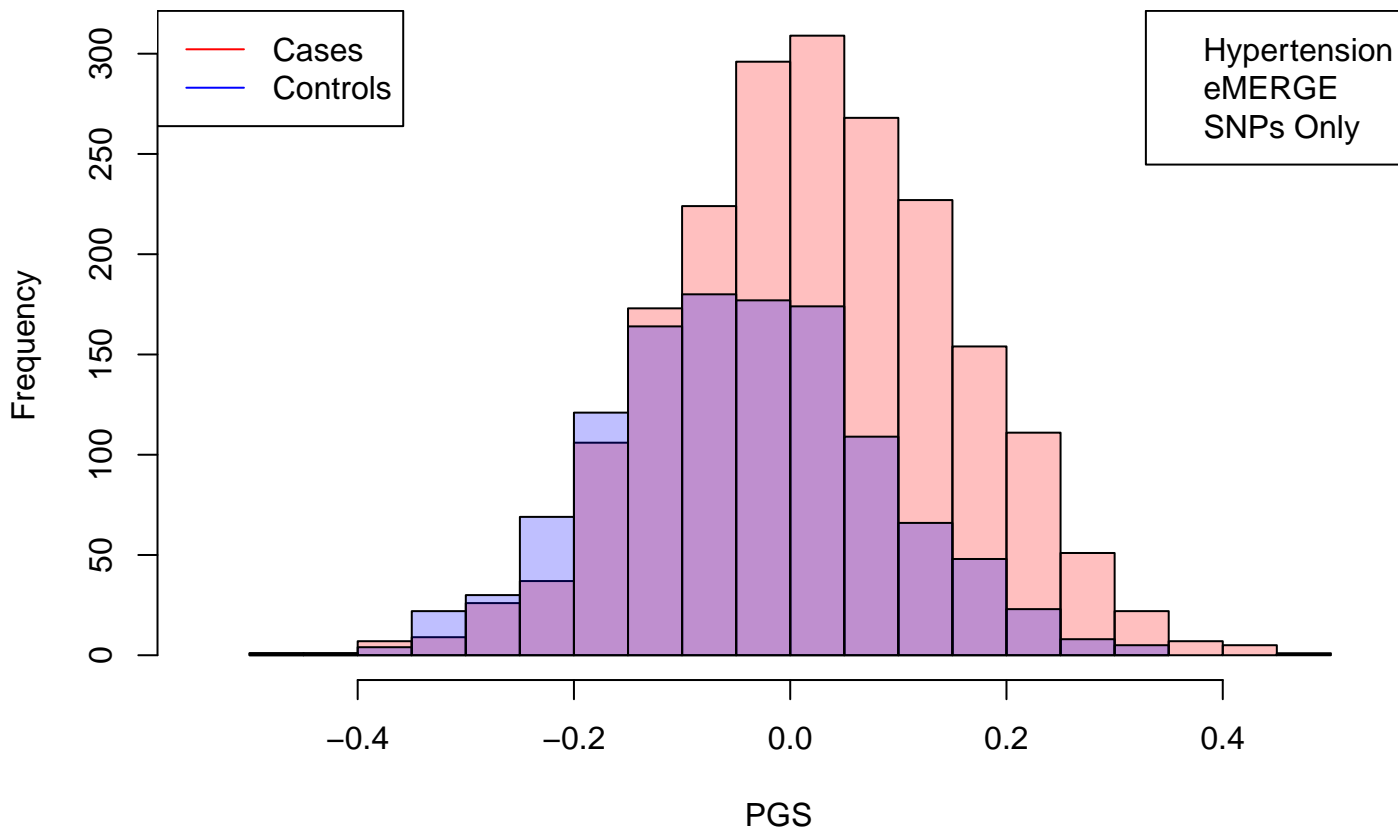

Supplement: Supplementary file 42 — LaTeX Supplementary File [file 41598_2019_51258_MOESM42_ESM.pdf]

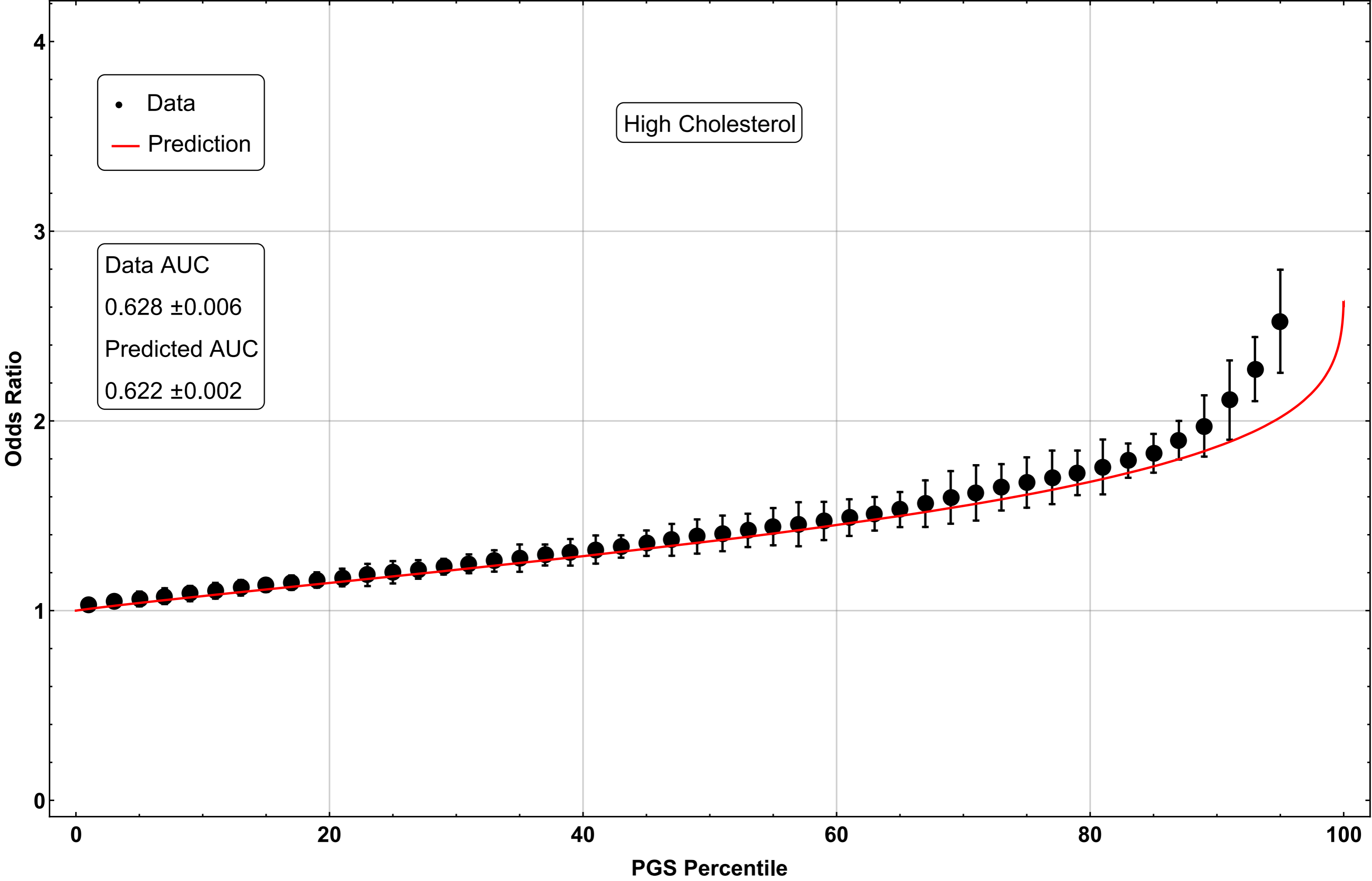

Supplement: Supplementary file 43 — LaTeX Supplementary File [file 41598_2019_51258_MOESM43_ESM.pdf]

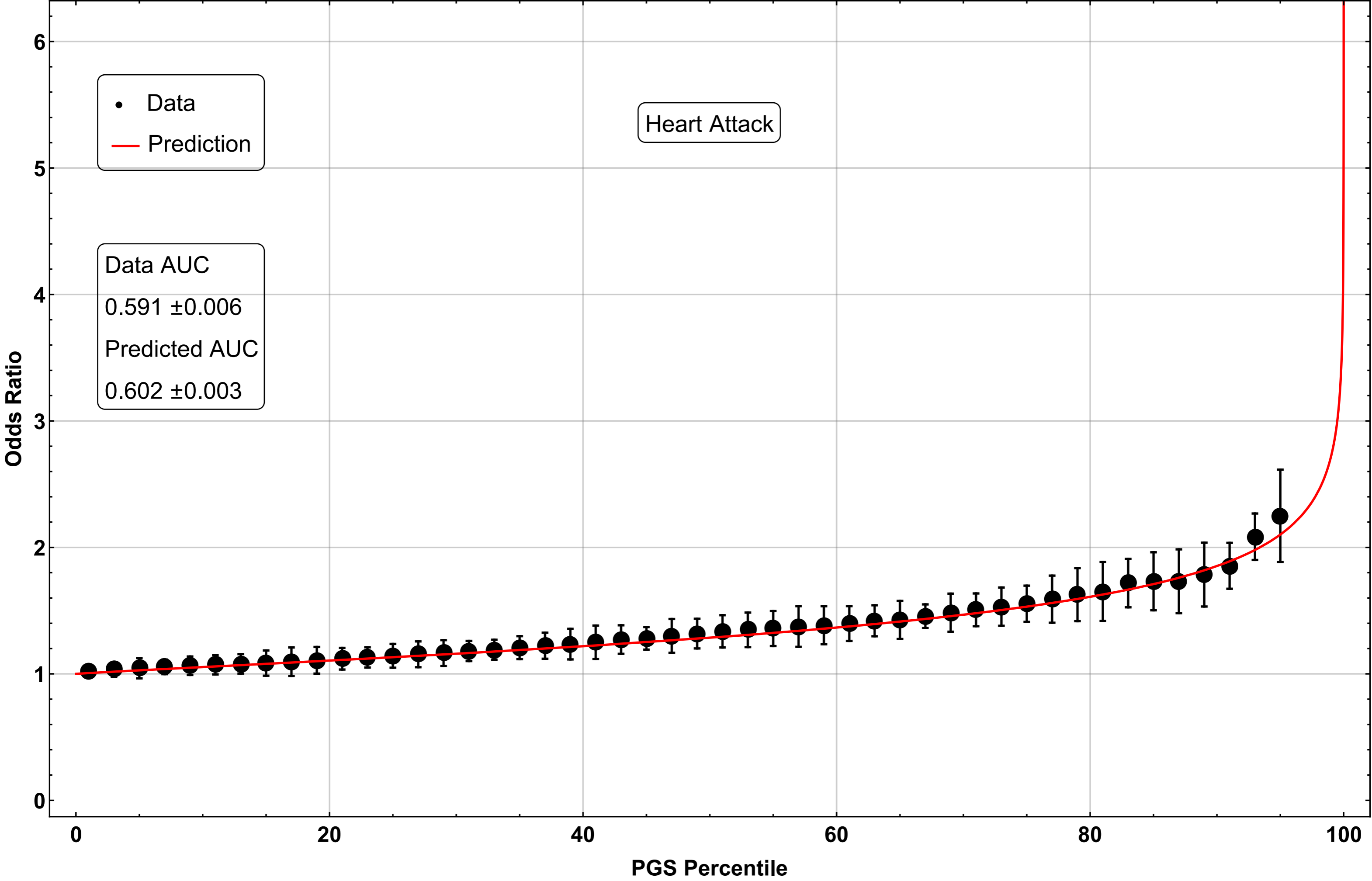

Supplement: Supplementary file 44 — LaTeX Supplementary File [file 41598_2019_51258_MOESM44_ESM.pdf]

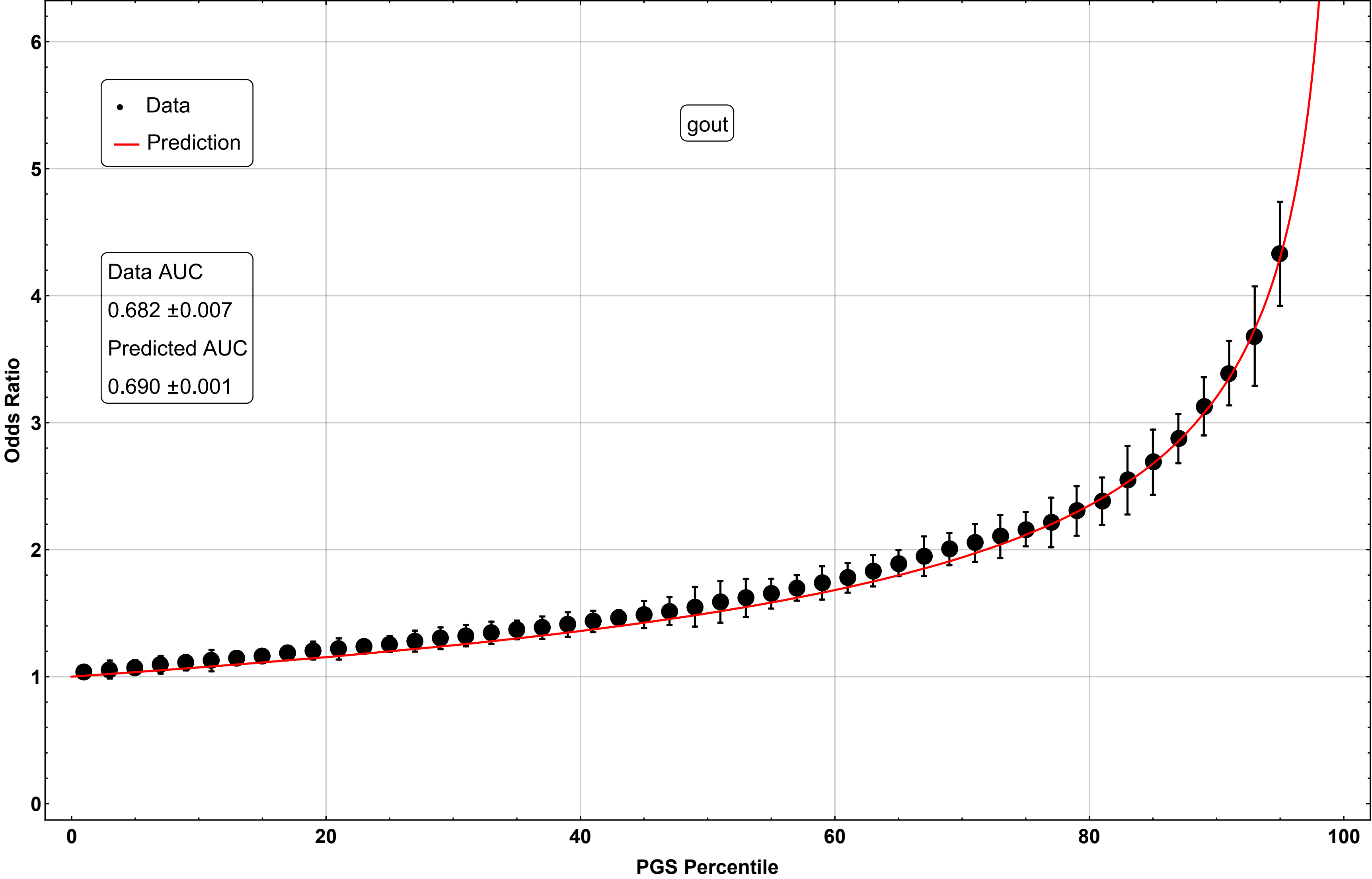

Supplement: Supplementary file 45 — LaTeX Supplementary File [file 41598_2019_51258_MOESM45_ESM.pdf]

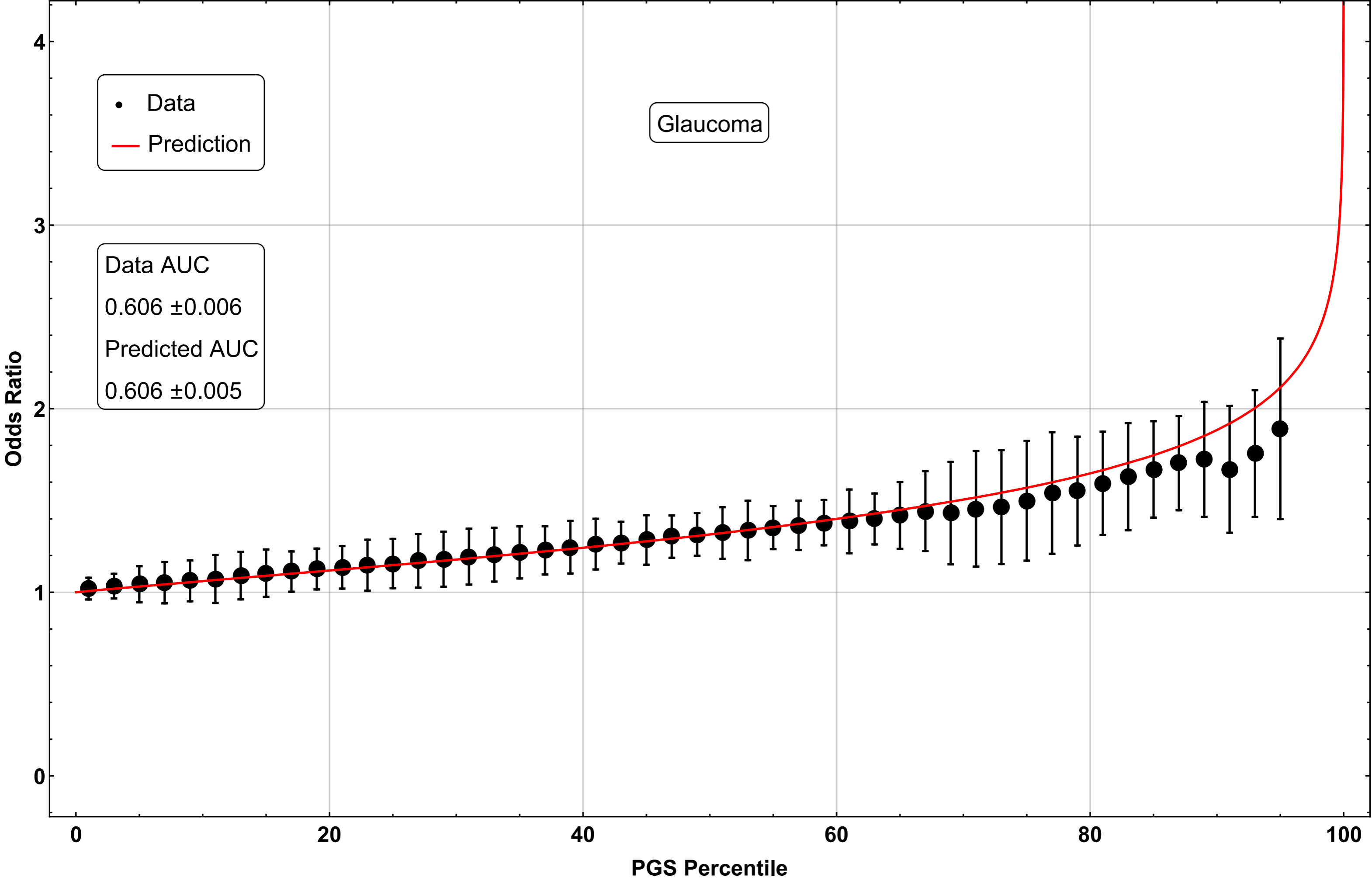

Supplement: Supplementary file 46 — LaTeX Supplementary File [file 41598_2019_51258_MOESM46_ESM.pdf]

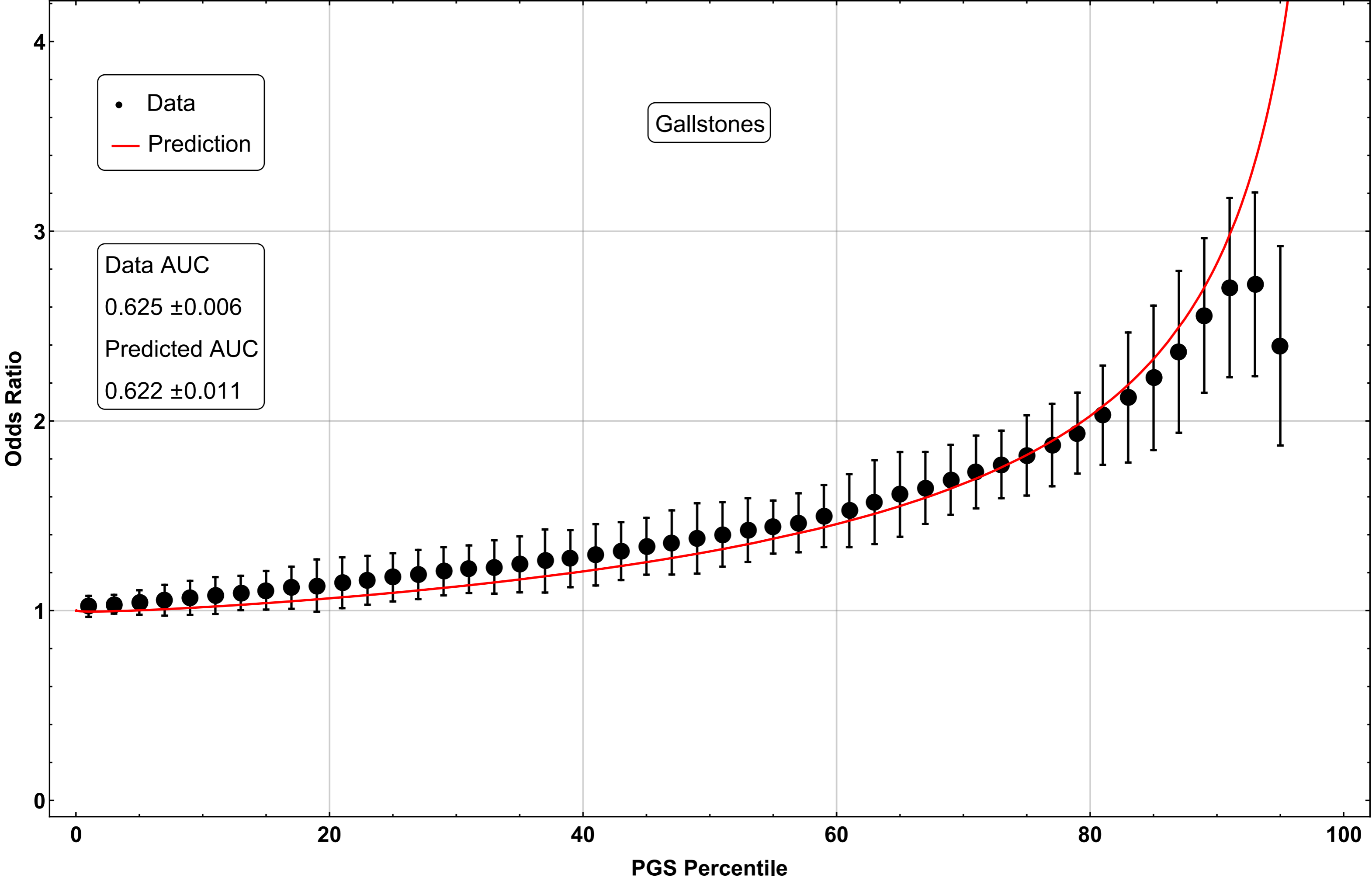

Supplement: Supplementary file 47 — LaTeX Supplementary File [file 41598_2019_51258_MOESM47_ESM.pdf]

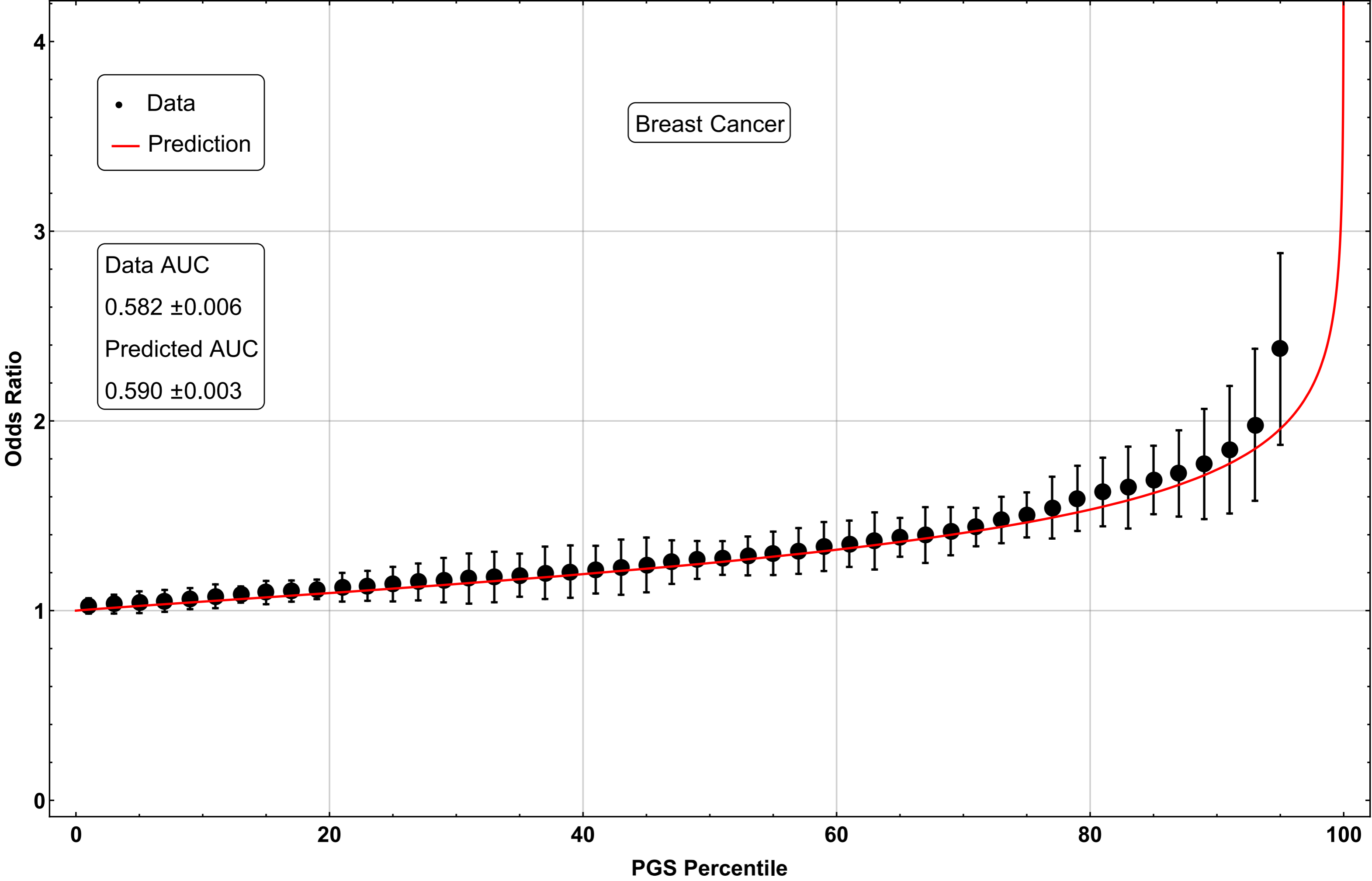

Supplement: Supplementary file 48 — LaTeX Supplementary File [file 41598_2019_51258_MOESM48_ESM.pdf]

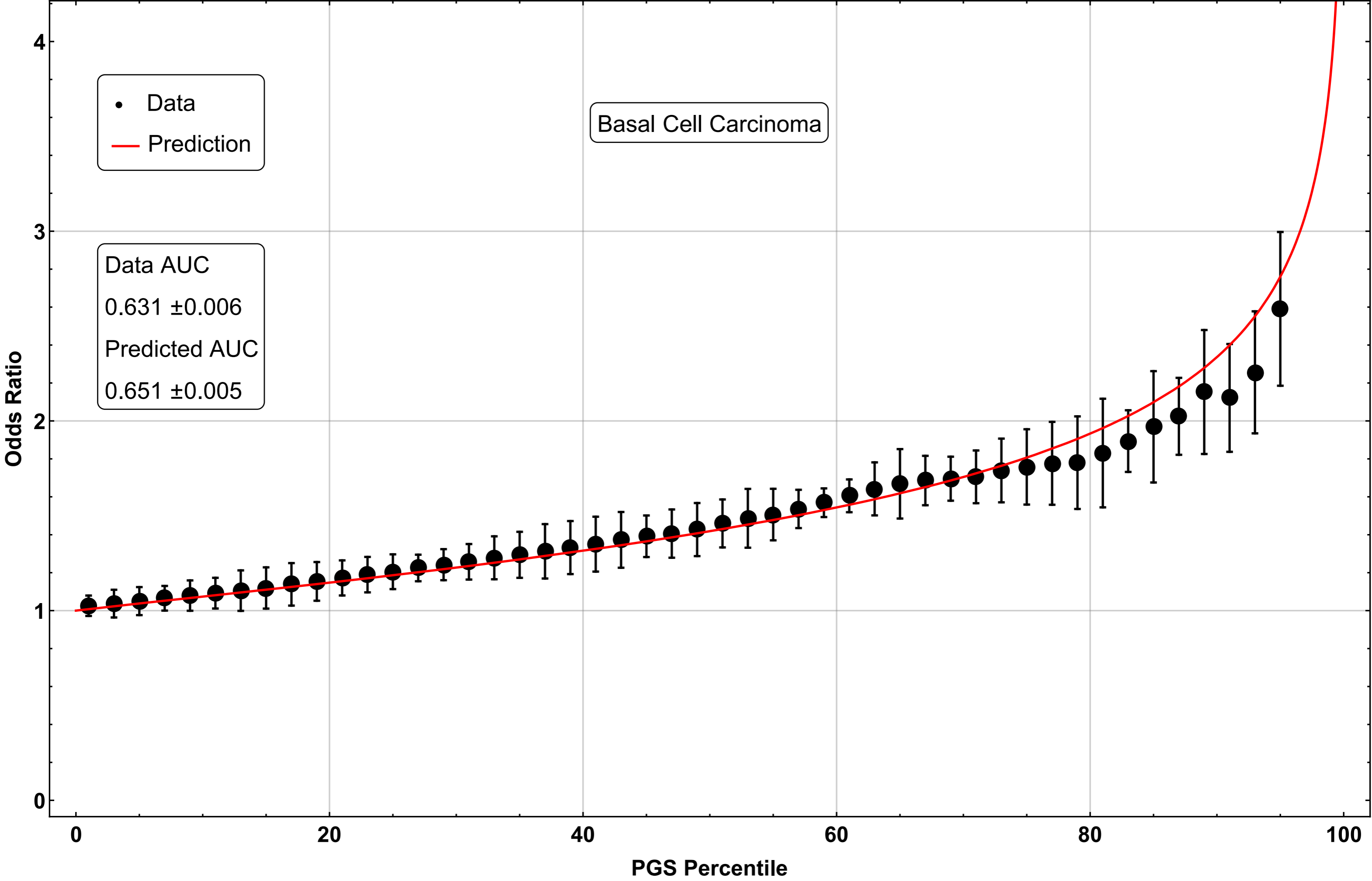

Supplement: Supplementary file 49 — LaTeX Supplementary File [file 41598_2019_51258_MOESM49_ESM.pdf]
