# Supplementary material for: Genomic Prediction of 16 Complex Disease Risks Including Heart Attack, Diabetes, Breast and Prostate Cancer
Source: Sci Rep. 2019 Oct 25;9:15286. doi: 10.1038/s41598-019-51258-x (PMC6814833; doi:10.1038/s41598-019-51258-x)
Supplement: Supplementary file 50 — Supplementary Info [file 41598_2019_51258_MOESM50_ESM.pdf]

Genomic Prediction Of 16 Complex Disease Risks Including Heart Attack, Diabetes, Breast and Prostate Cancer

Louis Lello *[1], Timothy G. Raben †[1], Soke Yuen Yong ‡[1], Laurent CAM Tellier §[2,3], and Stephen D.H. Hsu ¶[1,2,3]

[1]Department of Physics and Astronomy, Michigan State University
[2]Genomic Prediction, North Brunswick, NJ
[3]Cognitive Genomics Laboratory, Shenzhen Key Laboratory of Neurogenomics, China National GeneBank, BGI-Shenzhen, Shenzhen

September 19, 2019

Abstract

We construct risk predictors using polygenic scores (PGS) computed from common Single Nucleotide Polymorphisms (SNPs) for a number of complex disease conditions, using L1-penalized regression (also known as LASSO) on case-control data from UK Biobank. Among the disease conditions studied are Hypothyroidism, (Resistant) Hypertension, Type 1 and 2 Diabetes, Breast Cancer, Prostate Cancer, Testicular Cancer, Gallstones, Glaucoma, Gout, Atrial Fibrillation, High Cholesterol, Asthma, Basal Cell Carcinoma, Malignant Melanoma, and Heart Attack. We obtain values for the area under the receiver operating characteristic curves (AUC) in the range $\sim 0.58 - 0.71$ using SNP data alone. Substantially higher predictor AUCs are obtained when incorporating additional variables such as age and sex. Some SNP predictors alone are sufficient to identify outliers (e.g., in the 99th percentile of PGS) with $3 - 8$ times higher risk than typical individuals. We validate predictors out-of-sample using the eMERGE dataset, and also with different ancestry subgroups within the UK Biobank population. Our

*Corresponding Author; lellolou@msu.edu
†rabentim@msu.edu
‡yongsoke@msu.edu
§tellier@msu.edu
¶hsu@msu.edu

results indicate that substantial improvements in predictive power are attainable using training sets with larger case populations. We anticipate rapid improvement in genomic prediction as more case-control data become available for analysis.

Supplementary Information

A Genotype Quality Control

The main dataset used for training in this work is the 2018 release of the UK Biobank (the 2018 version corrected some issues with imputation, included sex chromosomes, etc). In all predictor training, we restricted our analysis to genetically British individuals (as defined using ancestry principal component analysis performed by UK Biobank) [1] . In 2018, the UK Biobank (UKBB) re-released the dataset representing approximately 500,000 individuals genotyped on two Affymetrix platforms - approximately 50,000 samples on the UKB BiLEVE Axiom array and the remainder on the UKB Biobank Axiom array. The genotype information was collected for 488,377 individuals for 805,426 SNPs which were then subsequently imputed to a much larger number of SNPs.

The imputed data set was generated using the set of 805,426 raw markers using the Haplotype Reference Consortium and UK10K haplotype resources. After imputation and initial QC, there were a total of 97,059,328 SNPs and 487,409 individuals. From this imputed data, further quality control was performed using Plink version 1.9. For out-of-sample testing of polygenic risk scores, imputed UK Biobank SNPs which survived the prior quality control measures, and are also present in a second dataset from the Electronic Medical Records and Genomics (eMERGE) study are kept. After keeping SNPs which are common to both the UK Biobank and eMERGE, 557,595 SNPs remained. Additionally SNPs and samples which had missing call rates exceeding 3% were excluded and SNPs with minor allele frequency below 0.1% were also removed so to avoid rare variants. This resulted in 468,514 SNPs and, upon restricting to genetically British, 408,954 people.

B Phenotype Quality Control

For model training which can be compared to true out-of-sample data, we focused on three case-control conditions which were present in both the UK Biobank and eMERGE datasets - Hypothyroidism, Type 2 Diabetes, and Hypertension. To select Type 2 Diabetes cases in UKBB, we identify individuals based on a doctor's diagnosis using the fields Diagnoses primary ICD10 or Diagnoses secondary ICD10. Specifically, any individual with ICD10 code E11.0-E11.9 (Non-insulin-dependent diabetes mellitus) in the Main Diagnosis or Secondary Diagnosis field. For training only, we wanted to exclude younger individuals who may still yet develop Type 2 Diabetes, so controls were selected using individuals in the remainder of the UKBB population not identified as cases and born on 1945 or earlier. This resulted in 18,194 cases and 108,726 controls among genetically British individuals.

For both Hypertension and Hypothyroidism, we used the field "Non-Cancer Illness Code

(self-reported)" to identify cases and controls. As in the case of type 2 diabetes, we exclude younger individuals as controls for Hypertension. This was not required for Hypothyroidism. Specifically, cases were identified by anyone with the code "1065" (Hypertension) in "Non cancer illness code (self-reported)" and the remainder of the UKBB population who was born before 1950 were selected as controls. This resulted in 109,662 cases and 140,689 controls for Hypertension. For Hypothyroidism, cases were identified by anyone with the code "1226" (Hypothyroidism/Myxoedema) in "Non cancer illness code (self-reported)" and the remainder of the UKBB population was used as a control. This resulted in 20,656 cases and 388,298 controls for Hypothyroidism.

For the following phenotypes we did not have true out of sample data, and so used the adjacent ancestry (AA) based testing procedure of Appendix D: Gout, Testicular Cancer, Gallstones, Breast Cancer, Atrial Fibrillation, Glaucoma, Type 1 Diabetes, High Cholesterol, Asthma, Basal Cell Carcinoma, Malignant Melanoma, Prostate Cancer, and Heart Attack. All conditions were identified using the fields "Non cancer illness code (self-reported)", "Cancer code (self-reported)" and "Diagnoses primary ICD10" or "Diagnoses secondary ICD10".

Cases and controls of the following non-cancer illnesses are identified using the field "Non-Cancer Illness Code (self-reported)": Gout, Gallstones, Atrial Fibrillation, Glaucoma, High Cholesterol, Asthma and Heart Attack. Cases for a specific non-cancer illness were identified as any individual with the following codes, and the remaining population are selected as a controls: Gout 1466, Gallstones 1162, Atrial Fibrillation 1471, Glaucoma 1277, High Cholesterol 1473, Asthma 1111, Heart Attack 1075. Cases and controls of the following cancer conditions were extracted from the field "Cancer Code (self-reported)": Testicular Cancer, Prostate Cancer, Breast Cancer, Basal Cell Carcinoma and Malignant Melanoma. Specifically, cases were identified as any individual with the following codes, and controls are the remainder of the population: Testicular Cancer 1045, Breast Cancer 1002, Basal Cell Carcinoma 1061, Malignant Melanoma 1059, Prostate Cancer 1044. To select Type 1 Diabetes cases in UKBB, we identify individuals based on a doctor's diagnosis using the fields "Diagnoses primary ICD10" or "Diagnoses secondary ICD10". Specifically, any individual with ICD10 code E10.0-E10.9 (Insulin-dependent diabetes mellitus) in the Main Diagnosis or Secondary Diagnosis field.

After identifying cases and controls in the whole UKBB population, we restricted our training set to "Genetically British" and our testing set to self-reported non-genetically-British whites. The number of cases and controls identified in this manner are listed in Table S1.

We include a supplementary table which outlines what fraction of cases and controls are male or female. We also include the mean year of birth for male/female cases/controls. This is shown in Table S2.

Condition	Cases (train)	Controls (train)	Cases (test)	Controls (test)
Gout	6,003	395,842	811	56,383
Gallstones	7,022	394,823	936	56,258
Atrial Fibrillation	3,502	398,343	420	56,774
Glaucoma	4,609	397,236	577	56,617
Type 1 Diabetes	2,734	399,111	388	56,806
High Cholesterol	52,398	349,447	6,937	50,257
Asthma	47,237	354,608	6,655	50,539
Basal Cell Carcinoma	4,132	397,713	577	56,617
Malignant Melanoma	3,301	398,544	444	56,750
Heart Attack	9,657	398,544	1,347	55,847
Prostate Cancer *	3,258	181,518	379	24,733
Breast Cancer *	9,177	207,892	1,344	30,738
Testicular Cancer *	716	184,060	91	25,021

Table S1: Table of number of cases and controls in training and testing sets for psuedo out-of-sample testing. Traits with (*) are trained and tested only on a single sex.

C Out-of-sample Quality Control

For out-of-sample testing, we use the 2015 release of the Electronic Medical Records and Genomics (eMERGE) study of approximately 15k individuals available on dbGaP [2]. The specific eMERGE data set used here refers to data downloaded from the dbGaP web site, under accession phs000360.v3.p1.(`https://www.ncbi.nlm.nih.gov/projects/gap/cgi-bin/study.cgi?study_id=phs000360.v3.p1`). The eMERGE dataset consists of 14,908 individuals with 561,490 SNPs which were genotyped on the Illumina Human 660W platform. The Plink 1.9 software is used for all further quality control. We first filter for SNPs which are common to the UK Biobank. SNPs and samples with missing call rates exceeding 3% are excluded and SNPs with minor allele frequency below 0.1% were also removed. This results in 557,595 SNPs and 14,906 individuals. Of these, the 468,514 SNPs which passed QC on the UK Biobank are used in training.

All eMERGE individuals in our dataset have self reported their ethnicity as white. Not all individuals in eMERGE are strictly cases or controls for any one particular condition. For Type 2 Diabetes, there are 1,921 identified cases and 4,369 identified controls. For Hypothyroidism there are 1084 identified cases and 3171 identified controls. For Hypertension, as the study focused on identifying individuals with Resistant Hypertension, there are two types of cases and two types of controls. Case group 1 consists of subjects with 4 or more medications simultaneous on at least 2 occasions greater than one month apart. Case group 2 has two outpatient (if possible) measurements of systolic blood pressure over 140 or diastolic blood pressure greater than 90 at least one month after meeting medication criteria while still on 3 simultaneous classes of medication AND has three simultaneous medications on at least two occasions greater than one month apart. Control group 2 consists of subjects with no evidence of Hypertension. Control group 1 consists of subjects with outpatient measure-

ments of SBP over 140 or DBP over 90 prior to beginning medication AND has only one medication AND has SBP $<$ 135 and DBP $<$ 90 one month after beginning medication. For model testing of Hypertension, we classified case group 1, case group 2 and control group 1 as cases, while control group 2 is used as controls. For Resistant Hypertension, we classified case group 1 and case group 2 as cases, while control group 2 is used as controls - control group 1 is excluded from this testing. The size of the self-reported white members of the groups are: case group 1 - 952, case group 2 - 406, control group 1 - 677, control group 2 - 1202.

The year of birth in eMERGE is given by decade, so the year of birth is taken to be the 5th year of the decade (i.e., if the decade of birth is 1940, then 1945 is used as year of birth). Some individuals did not have a year of birth listed - these individuals are included when testing models which did not feature age and sex as covariates, but are excluded when testing a model which included age. For obtaining age and sex effects, we used the entire UK Biobank for training as opposed to excluding younger participants as was done for the genetic models.

D Testing using Genetically Dissimilar Subgroups: Adjacent Ancestry Testing

For many case-control phenotypes, we do not have access to a second data set for proper out-of-sample testing. For these traits, we follow an adjacent ancestry (AA) testing procedure which was proposed and used in [3]. In this procedure, the predictor is trained on individuals of a homogeneous ethnic background: from UKBB we use genetically British individuals, defined using principal components analysis of population data. The predictor is then applied to individuals who are genetically dissimilar to the training set but not overly distant. For our testing set we use self-reported white (i.e., European) individuals (British/Irish/Any Other White) who are not in the cohort identified as genetically British. These individuals might be, for example, people of primarily Italian, Spanish, French, German, Russian, or mixed European ancestry who now live in the UK.

To identify the genetically British individuals, we follow the procedure in [1]. The top 20 principal components for the entire sampled population are provided directly from UK Biobank and the top 6 are used to identify genetically British individuals. We select individuals who self-report their ethnicity as "British" and use the outlier detection algorithm from the R-package "Aberrant" [4] to identify individuals using pairs of principal component vectors.

Aberrant uses a parameter which is the ratio of standard deviations of the outlying to normal individuals (λ) (Note λ here is a variable name used in Aberrant. It should not be confused with the lasso penalization parameter used in our optimization). This parameter is tuned to make a training set which is overly homogenous compared to those reported as genetically

British by the UKBB ($\lambda \sim 20$). Because Aberrant uses two inputs at a time, individuals to be excluded from training were identified using principal component pairs (first and second, third and fourth, fifth and sixth) and the union of these sets are the total group which is excluded in the final training set. There were a total of 402,937 individuals to be used in training after principal component filtering.

For this type of testing, the directly called gentoypes are used for training, cross-validation and testing (imputed SNPs are only used for true out-of-sample testing). First, only self-reported white individuals were selected (472,856) and then SNPs and samples with missing call rates exceeding 3% were removed, as were SNPs with minor allele frequency below 0.1% (all using Plink). This results in 658,543 SNPs and 459,039 total individuals which consists of 401,845 genetically British who are used for training and 57,194 non-British self-reported white individuals are used for final ancestry based out-of-sample testing.

E Odds Ratio Comparisons

Here we discuss here details about our odds ratio calculations and give further details about previous work done in the literature. The main points are recorded in Table **??**.

We collect odds ratio cumulant plots as a function of PGS percentile (i.e., a given value on the horizontal axis represents individuals with that PGS *or higher*) for the various phenotypes that were tested with the AA procedure described in Sec. 2 from the main document and reported in Table 2 from the main document. We also comment here on some of the more notable comparisons to previous methods used in the literature to analyze the genetic predictability of these phenotypes. It should be noted that some of these phenotypes - e.g., Asthma, Heart Attack, and High Cholesterol - have been heavily linked to other complex traits and future studies using multiple complex traits might greatly improve prediction.

Asthma, in Fig. S1, has long been known to have a significant genetic component. In this study odds ratios $\sim$ 3x are found for people with PGS scores in the 96th percentile and above. This compares favorably to the literature where 2.5x odds ratio increase at 95% confidence level was found for children with parents that have asthma [5]. GWAS studies [6] have shown that Asthma seems to be correlated with both hay fever and Eczema conditions. Although in performing this study, we did not find a strong predictor for Eczema, relevant data is available in UKBB and multi-phenotype studies could be performed in the future.

Atrial Fibrillation, seen in Fig. S1, is also known to have a genetic risk factor [7, 8]. Parental studies have shown a 1.4x odds ratio, but-although gene loci have been identified, genetic studies have not previously been successful in clinical settings [9]. In this work, PGS scores in the 96th percentile and above predict up to a 5x increase in odds.

Basal Cell Carcinoma, melanoma, and prostate cancer are some of many cancers that has

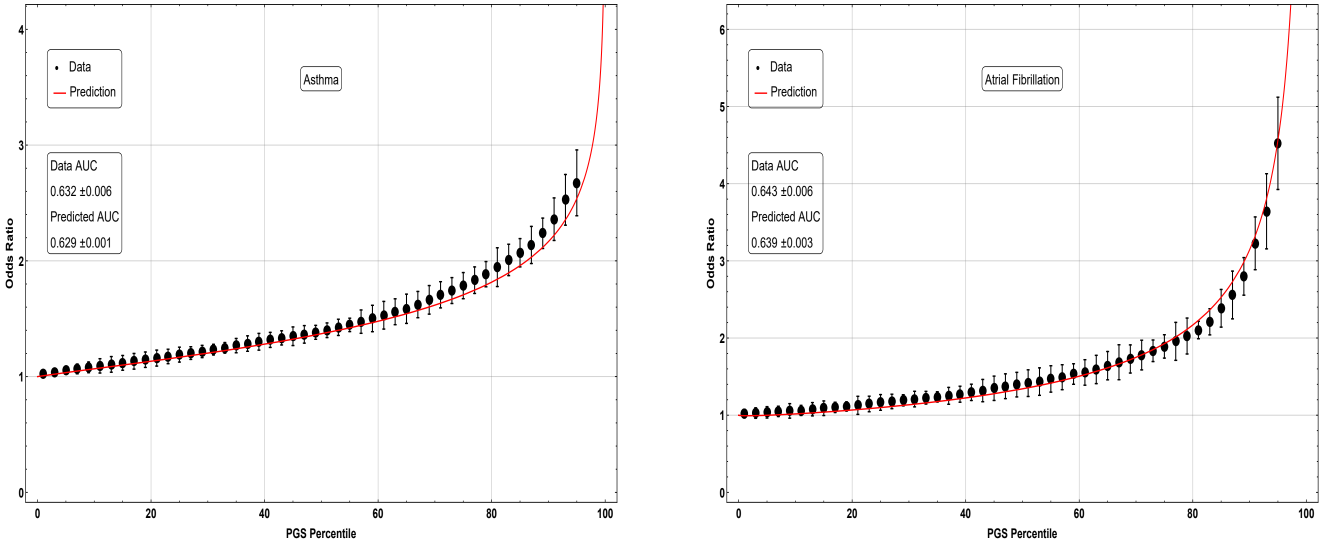

Figure S1: Odds ratio as a function of PGS percentile (i.e. scores at that point or above) for (left) Asthma and (right) Atrial Fibrillation.

been known to have a genetic component. Using genetic information, diagnosis status, and patient information–PGS type predictors have been built for a wide array of cancers [10]. The clinical utility of genomic prediction for melanoma has also been investigated [11].

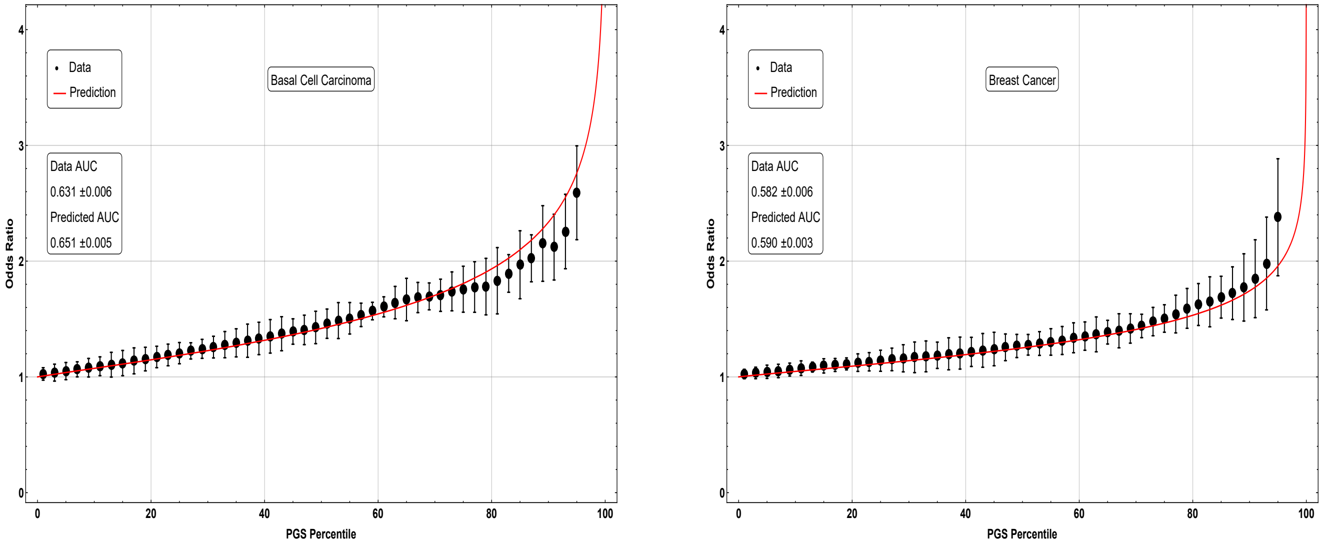

Figure S2: Odds ratio as a function of PGS percentile (i.e. scores at that point or above) for (left) Basal Cell Carcinoma and (right) Breast Cancer.

Breast Cancer, in Fig. S2, has long been evaluated with the understanding that there is a genetic risk component. Recent studies involving multi SNP prediction (77 SNPs) have been able to predict 3x odds increases for genetic outliers. This is consistent with our results for the highest genetic outliers although we used many more SNPS 480 ± 62.

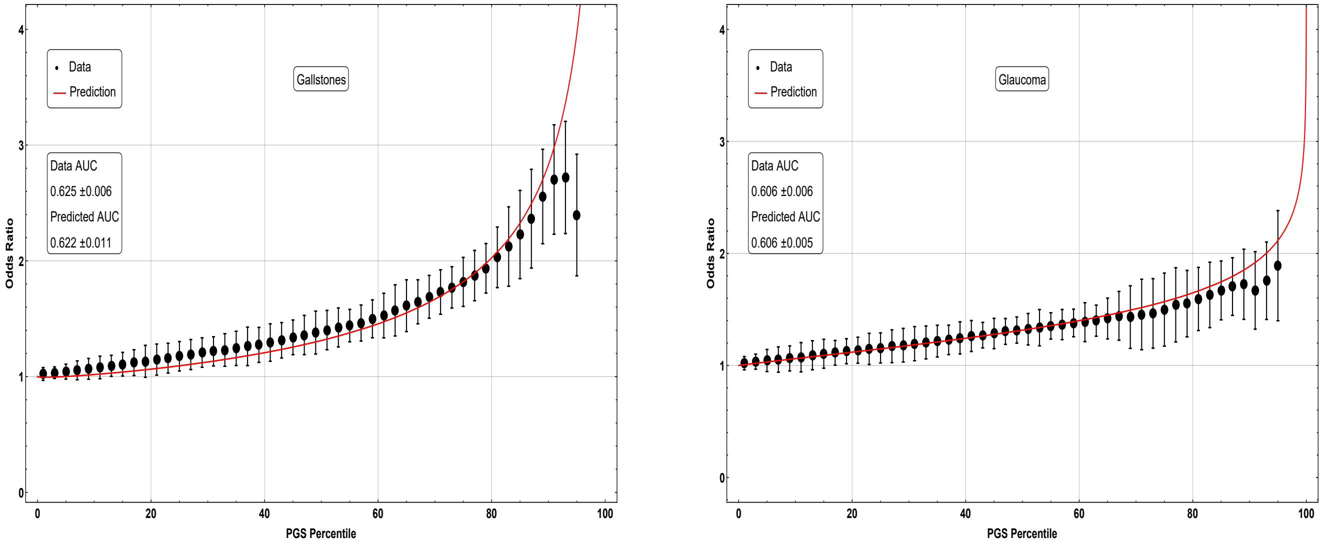

Figure S3: Odds ratio as a function of PGS percentile (i.e. scores at that point or above) for (left) Gallstones and (right) Glaucoma.

Recent reviews suggest that much of the risk leading to a higher probability of having Gallstones is associated with non-genetic factors. However, in Fig. S3, we find that 90th percentile and above PGS is be associated with a 3x odds increase. Recent GWAS studies for gallstones [12].

While there are a variety of relevant environmental factors, recent reviews of the genetics of Glaucoma [13] highlight that GWAS studies have found 25 genic regions with odds ratios above 1x. The highest being 2.80x [14]. In Fig. S3 we see similar odds ratios for extreme PGS. GWAS glaucoma studies for specific ethnic groups have found even larger odds ratios[14–22]. (Much larger odd ratios, 5-15x, can be found if you restrict to GWAS of just older populations[23].)

Gout, seen in Fig. S4, reaches an extremely high 4.5x odds ratio for PGS in the 96th percentile and above. Reviews of Gout [24] have noted both a strong familial heritability and known GWAS loci, but we are not aware of previously-computed odds ratios this large solely due to genetics.

Much work has been done on the genetics of Hypertension. Researchers have built predictors with odds ratio 2x for the 90th percentile [25], comparable to the results found here. Hypertension reaches a high odds ratio of 3.5x for the 96th percentile while *resistant* Hypertension reaches 5x for the 96th percentile. The odds ratio obtained in the eMERGE data set is shown in Fig. 10 from the main documentand for resistant hypertesion in Fig. 11 from the main document.

For Hypothyroidism, researchers have used summary statistics and thyrotropin levels were used to predict Hypothyroidism [26], but the authors are unaware of a SNP based predictor in the literature. Hypothyroidism reaches an odds ratio of 3x. The odds ratios obtained in

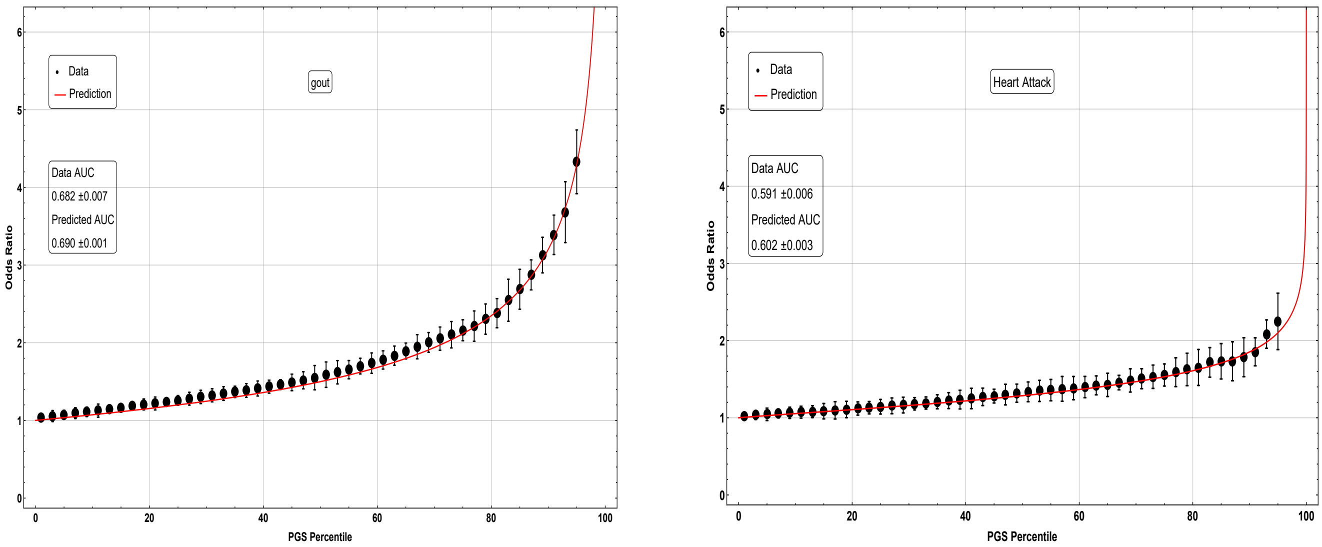

Figure S4: Odds ratio as a function of PGS percentile (i.e. scores at that point or above) for (left) Gout and (right) Heart Attack.

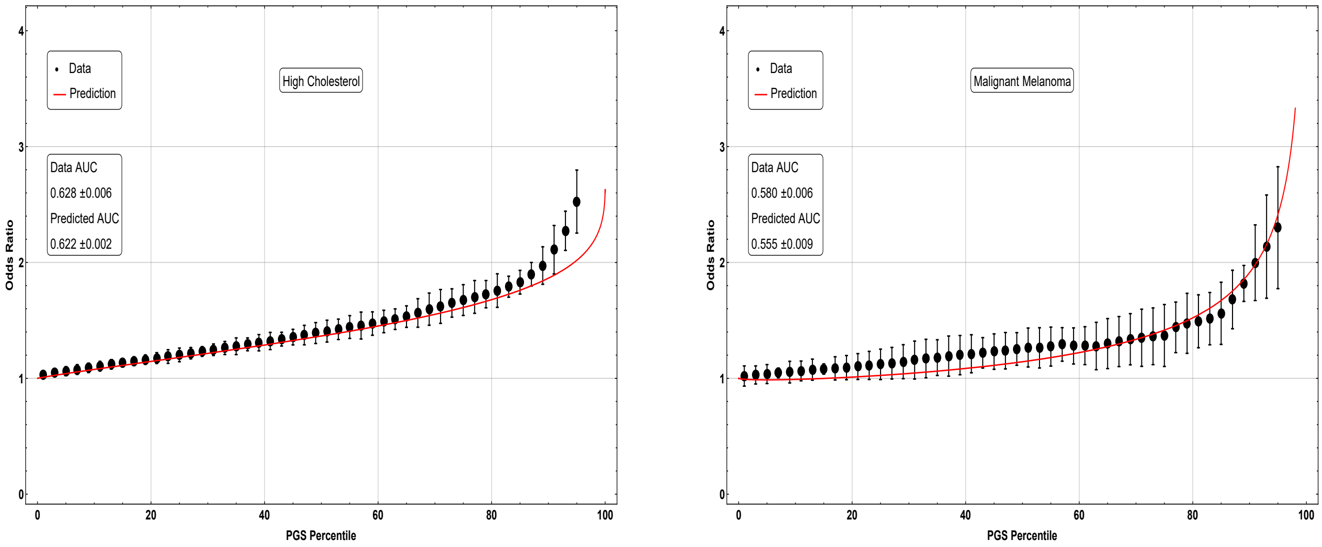

Figure S5: Odds ratio as a function of PGS percentile (i.e. scores at that point or above) for (left) High Cholesterol and (right) Malignant Melanoma.

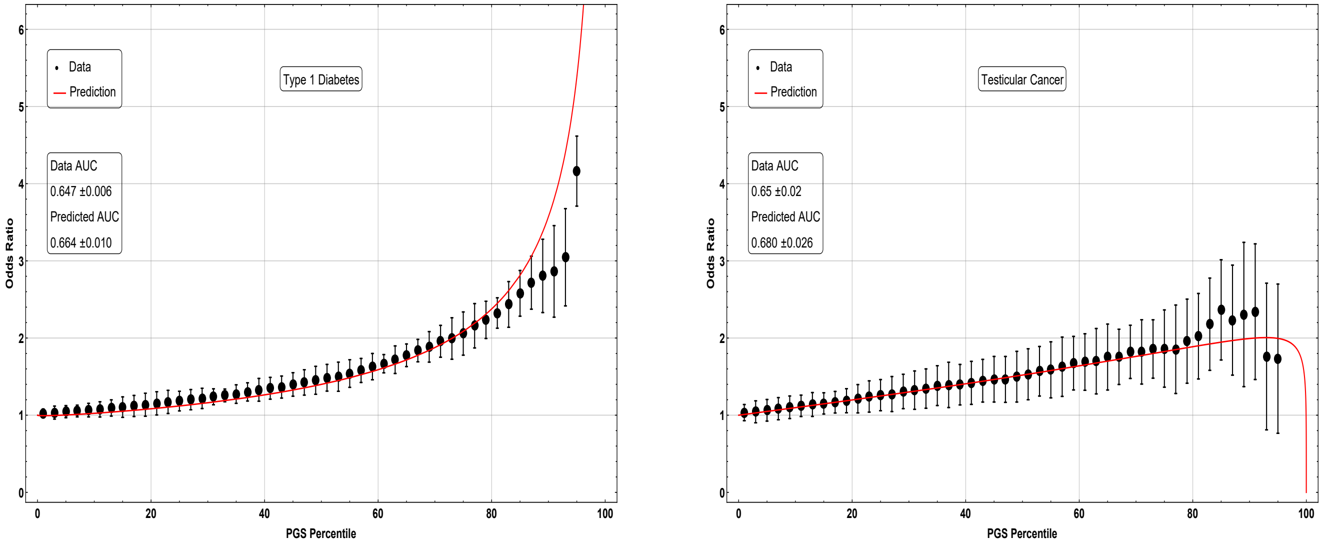

Figure S6: Odds ratio as a function of PGS percentile (i.e. scores at that point or above) for (left) Type 1 Diabetes and (right) Testicular Cancer. Note that the dip at extreme PGS values in the predicted Testicular Cancer curve may be related to a small number of available cases; the cases and controls are not well fit by two separate Gaussian distributions.

the eMERGE data set is shown in Fig. 9 from the main document.

There is a wide ranging literature covering the genetics and heritability of Type 1 Diabetes. In Fig. S6 we see a large 4.5x odds ratio for extreme PGS. Notably, the literature has identified genetic prediction to be extremely useful in differentiating between Type 1 and Type 2 Diabetes [27–29] and in identifying β cell autoimmunity [30], which is highly correlated with diabetes.

Much work has been done on the genetics and risk factors of type 2 diabetes [31–33]. Recently, Khera et al. reached an odds ratio of 2.5x [34] for the 90th percentile in their predictor. To compare, the type 2 diabetes produced here reaches an odds ratio of just under 2.5x at the 90th percentile, similar to the literature, and reaches an odds ratio of 3.5x for the 96th percentile. The odds ratio obtained in the eMERGE data set is shown in Fig. 12 from the main document.

Prostate Cancer is the most common gender specific cancer in men. The odds ratio for AA testing can be seen in Fig. S7. It has long been known that age is a significant risk factor for prostate cancer, but GWAS studies have shown that there is a significant genetic component [35]. Additionally, it has been shown, using genome wide complex trait analysis (GCTA), that variants with minor allele frequency $0.1 - 1\%$ make up an important contribution to "missing heritablity" for men of African ancestry [36]. This study includes some SNP variants with minor allele frequency as low as 0.1%, so our model might include some of this contribution. Additionally genetic screening for prostate cancer has also been considered[37].

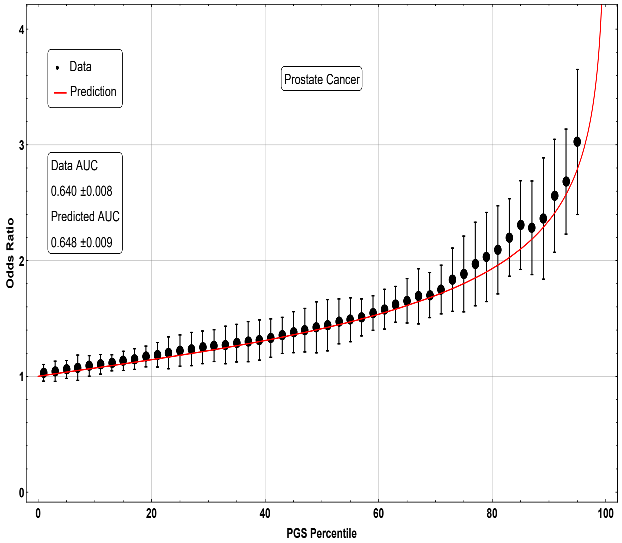

Figure S7: Odds ratio as a function of PGS percentile (i.e. scores at that point or above) for Prostate Cancer.

F Model Training Algorithm

In all calculations, we use a custom implementation of LASSO regression (Least Absolute Shrinkage and Selection Operator) written in the Julia language. This is the same implementation used in [38]. Given a set of samples $i = 1, 2, ..., n$ with a set of p SNPs, the phenotype y_i and state of the jth SNP, X_{ij}, are observed. X_{ij} is an n x p matrix which contains the number of copies of the minor allele and any missing values are replaced with the SNP average. The L_1 penalized regression, LASSO, seeks to minimize the objective function

$$\mathcal{O}_\lambda(\vec{\beta}) = \frac{1}{2}||\vec{y} - X\vec{\beta}||^2 + n\lambda||\vec{\beta}||_1 \tag{S1}$$

where $|\vec{v}|_1 = \sum_i^n |v_i|$ is the L_1 norm, $||\vec{v}|| = \sum_i^n v_i^2$ is the L_2 norm and λ is a tuneable hyperparameter. The solution is given in terms of the soft-thresholding function as

$$\begin{aligned} S(z,\gamma) &= \text{sgn}(z)\text{max}(|z| - \gamma, 0) \\ \beta_j^* &= \frac{1}{\sum_{i=1}^n X_{ij}^2} S\left(\sum_{i=1}^n \left[X_{ij}y_i - \sum_{k\neq j} X_{ij}X_{ik}\beta_k\right], n\lambda\right) \end{aligned} \tag{S2}$$

The penalty term affects which elements of $\vec{\beta}$ have non-zero entries. The value of λ is first chosen to be the maximum value such that all β_i are zero, and it is then decreased, allowing more nonzero components in the predictor. For each value of λ, $\vec{\beta}^*(\lambda_n)$ is obtained using the

previous values of $\vec{\beta}^*(\lambda_{n-1})$ (warm start) and coordinate descent. The Donoho-Tanner phase transition [39] describes how much data is required to recover the true nonzero components of the linear model and suggests that we expect to recover the true signal with s SNPs when the number of samples is $n \sim 30s - 100s$ (see [40, 41]). For a more complete description of the algorithm, see [38].

For all three conditions which are available in eMERGE, we withhold a subset of 1000 cases and 1000 controls from the training set to be set aside for cross-validation. We repeated this 5 times with non-overlapping cross-validation sets. With training and cross-validation sets constructed, we first perform a GWAS on the training set and select the rank ordered top 50,000 p-value SNPs. We then use these SNPs as input to the LASSO algorithm and finally apply the predictor to the corresponding cross-validation set in order to select the value of λ. For conditions which AA testing is used, we use cross-validation sets of 500 cases and 500 controls to tune our model.

Because individual SNPs are uncorrelated to year of birth and sex, we are able to regress on SNPs independently of age and sex. To train combined models, which include SNPs, age and sex, we perform LASSO on SNPs alone and least squares regression on age and sex only, then add the two predictor scores together. We tested for whether an improvement in AUC is achieved through a simultaneous regression using polygenic score (PGS), age, and sex as covariates, but found this to give similar AUC as doing the regressions independently and adding the results (to within a few % accuracy).

G Analytic AUC and Risk

While much of this section is well understood, we include a summary to define terminology and for reference. By assuming that cases and controls have PGS distribution which is Gaussian, we can analytically calculate quantities for genetic prediction. For example, we can calculate an AUC and see how it corresponds to to an odds ratio for various distributional parameters. For additional discussion, we refer the interested reader to [42].

Assume a case-control phenotype and that the cases and controls have Gaussian distributed PGS. Letting $i = \{0, 1\}$ represent controls and cases respectively, the distribution of scores can be written

$$\begin{aligned} f(x) &= \frac{1}{n_0 + n_1} \sum_{i=0,1} n_i f_i(x) \\ f(x, \mu_i, \sigma_i) &\equiv f_i(x) = \frac{1}{\sqrt{2\pi}} \mathrm{Exp}\left(\frac{-(x-\mu_i)^2}{2\sigma_i^2} \right) , \end{aligned} \tag{S1}$$

and n_i represents the total number of cases/controls. For completeness, we recall the defintion

of the error function here

$$\mathrm{Erf}(x) \equiv \frac{2}{\sqrt{\pi}} \int_0^x e^{-t^2}\ dt \quad .$$

AUC

First we need to generate an ROC curve of *false positive rate* (FPR) vs *true positive rate* (TPR).

$$\begin{aligned}
FPR(z,\mu_0,\sigma_0) &\equiv \frac{\text{false positives}}{\text{false positives} + \text{true negatives}} = \frac{\int_z^\infty n_0 f_0(x)dx}{\int_z^\infty n_0 f_0(x)dx + \int_{-\infty}^z n_0 f_0(x)dx} && \text{(S2)}\\
&= \int_z^\infty \frac{1}{\sqrt{2\pi}} \mathrm{Exp}\left(\frac{-(x-\mu_0)^2}{2\sigma_0}\right) dx = \frac{1}{2}\left(1 - \mathrm{Erf}\left(\frac{z-\mu_0}{\sqrt{2}\sigma_0}\right)\right) && \text{(S3)}\\
&= 1 - \Phi\left(\frac{z-\mu_0}{\sigma_0}\right) && \text{(S4)}\\
TPR(z,\mu_1,\sigma_1) &\equiv \frac{\text{true positives}}{\text{true positives} + \text{false negatives}} = \frac{1}{2}\left(1 - \mathrm{Erf}\left(\frac{z-\mu_1}{\sqrt{2}\sigma_1}\right)\right) && \text{(S5)}\\
&= 1 - \Phi\left(\frac{z-\mu_1}{\sigma_1}\right) . && \text{(S6)}
\end{aligned}$$

The AUC is then defined as the area under the ROC curve,

$$\begin{aligned}
AUC(\mu_0,\sigma_0,\mu_1,\sigma_1) &= \int_{-\infty}^\infty TPR(FPR(z,\mu_0,\sigma_0),\mu_1,\sigma_1)dz \\
&= \int_{-\infty}^\infty TPR(z,\mu_1,\sigma_1)\partial_z FPR(z,\mu_0,\sigma_0)dz && \text{(S7)}\\
&= \int_{-\infty}^\infty \frac{1}{2}\left(1 - \mathrm{Erf}\left(\frac{z-\mu_1}{\sqrt{2}\sigma_1}\right)\right)\left(\frac{\mathrm{Exp}\left(\frac{-(z-\mu_0)^2}{2\sigma_0^2}\right)}{\sqrt{2\pi}\sigma_0}\right) dz && \text{(S8)}\\
&= \frac{1}{2} - \frac{\sigma_1}{2\sqrt{\pi}\sigma_0}\int_{-\infty}^\infty \mathrm{Erf}(y)\mathrm{Exp}\left(-\left(\frac{\sigma_1}{\sigma_0}y + \frac{\mu_1-\mu_0}{\sqrt{2}\sigma_0}\right)^2\right) dy && \text{(S9)}\\
&= \frac{1}{2}\left(1 + \mathrm{Erf}\left(\frac{\mu_1-\mu_0}{\sqrt{2(\sigma_1^2+\sigma_0^2)}}\right)\right) = \Phi\left(\frac{\mu_1-\mu_0}{\sqrt{(\sigma_1^2+\sigma_0^2)}}\right) , && \text{(S10)}
\end{aligned}$$

in agreement with Eq.(4.1 from the main document). Note that the AUC is independent of the number of cases and controls.

Risk and Odds

There are two standard ways in the literature to classify the increased likelihood of a disease at a higher z-score.

Risk Ratio represents the ratio between (a) the number of cases at a particular z-score and above over the total number of people at z-score and above to (b) the total number of cases over the total number of cases and controls.

$$RR(\mu_0, \sigma_0, \mu_1, \sigma_1, n_0, n_1) = \frac{\left(\int_z^\infty n_1 f_1(x)dx\right) / \left(\int_z^\infty (n_1 f_1(x) + n_0 f_0(x))dx\right)}{n_1/(n_0 + n_1)} \tag{S11}$$

$$RR(\mu_0, \sigma_0, \mu_1, \sigma_1, r) = \left(\frac{1}{r} + 1\right)\left(1 + \frac{1 - \text{Erf}\left(\frac{z-\mu_0}{\sqrt{2}\sigma_0}\right)}{1 - \text{Erf}\left(\frac{z-\mu_1}{\sqrt{2}\sigma_1}\right)}\right)^{-1} \tag{S12}$$

$$= \left(\frac{1}{r} + 1\right)\left(1 + \frac{1 - \Phi\left(\frac{z-\mu_0}{\sigma_0}\right)}{1 - \Phi\left(\frac{z-\mu_1}{\sigma_1}\right)}\right)^{-1}, \tag{S13}$$

where we can note that the Risk Ratio only depends on the ratio $r \equiv n_1/n_0$.

Odds Ratio represents the ratio between (a) the number of cases at a particular z-score and above over the number of controls at a particular z-score and above to (b) the total number of cases over the total number of controls

$$OR(\mu_0, \sigma_0, \mu_1, \sigma_1, n_0, n_1) = \frac{\left(\int_z^\infty n_1 f_1(x)dx\right) / \left(\int_z^\infty n_0 f_0(x)dx\right)}{n_1/n_0} \tag{S14}$$

$$OR(\mu_0, \sigma_0, \mu_1, \sigma_1) = \frac{1 - \text{Erf}\left(\frac{z-\mu_1}{\sqrt{2}\sigma_1}\right)}{1 - \text{Erf}\left(\frac{z-\mu_0}{\sqrt{2}\sigma_0}\right)} = \frac{1 - \Phi\left(\frac{z-\mu_1}{\sigma_1}\right)}{1 - \Phi\left(\frac{z-\mu_0}{\sigma_0}\right)}, \tag{S15}$$

which is *independent* of n_1 and n_0. This is the result Eq.(4.2 from the main document).Note that in the *rare disease limit* (RDL)

$$n_1 << n_0 \qquad \text{and} \qquad n_1\left(1 - \text{Erf}\left(\frac{z-\mu_1}{\sqrt{2}\sigma_1}\right)\right) << n_0\left(1 - \text{Erf}\left(\frac{z-\mu_0}{\sqrt{2}\sigma_0}\right)\right), \tag{S16}$$

the risk ratio and odds ratio agree

$$RR(\mu_0, \sigma_0, \mu_1, \sigma_1, r) \xrightarrow{\text{RDL}} OR(\mu_0, \sigma_0, \mu_1, \sigma_1)\,. \tag{S17}$$

PGS percentile: In either case, we would like to know the risk or odds ratio in terms of the percentage of people with a particular z-score and above. We can define this percentile function as

$$\begin{aligned} P(z, &\mu_0, \sigma_0, n_0, \mu_1, \sigma_1, n_1) = \frac{1}{n_0 + n_1} \int_{\infty}^{z} (n_0 f_0(x) + n_1 f_1(x)) dx \\ &= \frac{1}{2(1+r)} \left(1 + \text{Erf}\left(\frac{z - \mu_0}{\sqrt{2}\sigma_0}\right) + r\left(1 + \text{Erf}\left(\frac{z - \mu_1}{\sqrt{2}\sigma_1}\right)\right)\right) \\ &= \frac{1}{1+r} \left(\Phi\left(\frac{z - \mu_0}{\sigma_0}\right) + r\Phi\left(\frac{z - \mu_1}{\sigma_1}\right)\right) = P(z, \mu_0, \sigma_0, \mu_1, \sigma_1, r)\,. \end{aligned} \tag{S18}$$

Combining Eq.(4.1 from the main document), Eq.(4.2 from the main document), and Eq.(S18) we can plot the odds ratio in terms of the distributional parameters as seen in Fig. S8.

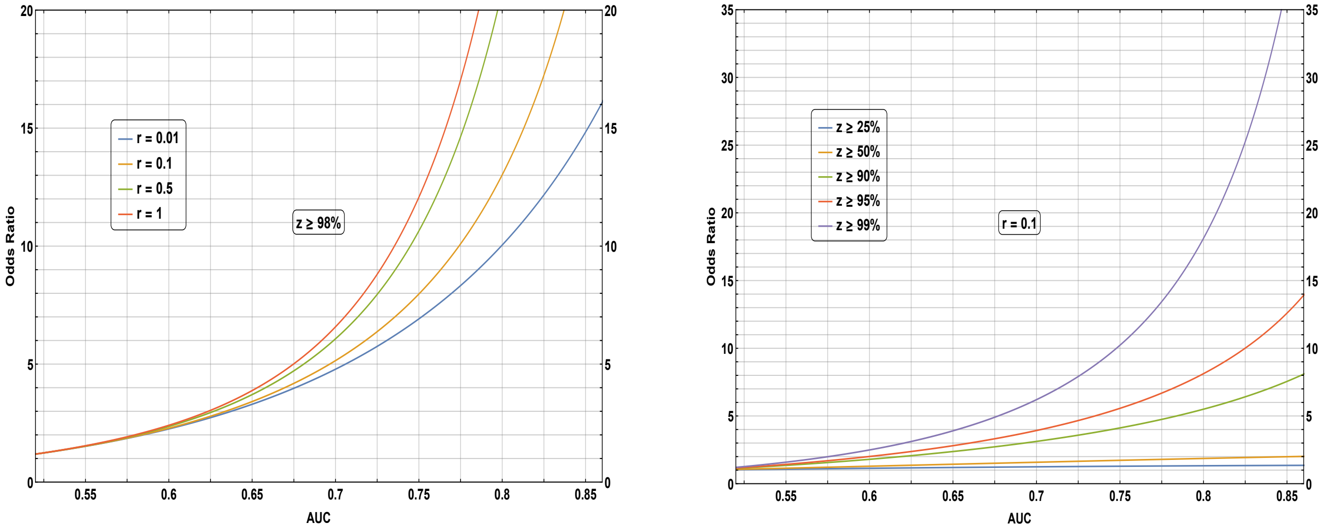

Figure S8: Odds ratio (assuming two displaced Gaussian distributions) as a function of AUC. Left: for z-scores above the 98th percentile at various values of the ratio of cases to controls r. Right: for case to control ratio $r = 0.1$ at various z-score percentiles. Assuming a population-representative sample, r is the prevalence of the disease in the general population.

Condition	% Female		Mean Birth Year (Female)	
	Cases	Controls	Cases	Controls
Gout	7.35	54.98	1946.4	1951.5
Gallstones	77.59	53.87	1949.0	1951.6
Atrial Fibrillation	31.06	54.48	1945.8	1951.5
Glaucoma	46.91	54.36	1946.5	1951.5
Type 1 Diabetes	41.45	54.36	1950.4	1951.5
High Cholesterol	42.98	55.95	1946.7	1952.0
Asthma	57.48	53.85	1952.0	1951.4
Basal Cell Carcinoma	58.40	54.23	1948.5	1951.5
Malignant Melanoma	58.88	54.24	1949.6	1951.5
Heart Attack	19.68	55.11	1945.9	1951.5
Prostate Cancer *	0.0	0.0	NA	NA
Breast Cancer *	100.0	100.0	1946.0	1951.6
Testicular Cancer *	0.0	0.0	NA	NA
Condition	% Male		Mean Birth Year (Male)	
	Cases	Controls	Cases	Controls
Gout	92.65	45.02	1948.5	1951.2
Gallstones	22.41	46.13	1947.5	1951.1
Atrial Fibrillation	68.94	45.52	1946.4	1951.1
Glaucoma	53.09	45.64	1946.5	1951.1
Type 1 Diabetes	58.55	45.64	1949.0	1951.1
High Cholesterol	57.02	44.05	1947.3	1951.8
Asthma	42.52	46.15	1952.0	1951.0
Basal Cell Carcinoma	41.60	45.77	1947.4	1948.5
Malignant Melanoma	41.12	45.76	1948.2	1951.1
Heart Attack	80.32	44.89	1946.2	1945.9
Prostate Cancer *	100.0	100.0	1944.3	1951.2
Breast Cancer *	0.0	0.0	NA	NA
Testicular Cancer *	100.0	100.0	1953.2	1951.5

Table S2: Table of fraction of cases and controls and mean year of birth by sex for psuedo out-of-sample testing. Traits with (*) are trained and tested only on a single sex.

References

1. Bycroft, C. *et al.* Genome-wide genetic data on 500,000 UK Biobank participants. *bioRxiv.* doi:10.1101/166298. eprint: https://www.biorxiv.org/content/early/2017/07/20/166298.full.pdf. https://www.biorxiv.org/content/early/2017/07/20/166298 (2017) (cit. on pp. 3, 6).
2. McCarty, C. A. *et al.* The eMERGE Network: a consortium of biorepositories linked to electronic medical records data for conducting genomic studies. *BMC medical genomics* **4,** 13 (2011) (cit. on p. 5).
3. Marquez-Luna, C. *et al.* Modeling functional enrichment improves polygenic prediction accuracy in UK Biobank and 23andMe data sets. *bioRxiv.* doi:10.1101/375337. eprint: https://www.biorxiv.org/content/early/2018/07/24/375337.full.pdf. https://www.biorxiv.org/content/early/2018/07/24/375337 (2018) (cit. on p. 6).
4. Bellenguez, C. *et al.* A robust clustering algorithm for identifying problematic samples in genome-wide association studies. *Bioinformatics* **28,** 134–135 (2011) (cit. on p. 6).
5. Dold, S., Wjst, M, Von Mutius, E, Reitmeir, P & Stiepel, E. Genetic risk for asthma, allergic rhinitis, and atopic dermatitis. *Archives of disease in childhood* **67,** 1018–1022 (1992) (cit. on p. 7).
6. Ferreira, M. A. *et al.* Shared genetic origin of asthma, hay fever and eczema elucidates allergic disease biology. *Nature genetics* **49,** 1752 (2017) (cit. on p. 7).
7. Gudbjartsson, D. F. *et al.* Variants conferring risk of atrial fibrillation on chromosome 4q25. *Nature* **448,** 353 (2007) (cit. on p. 7).
8. Benjamin, E. J. *et al.* Variants in ZFHX3 are associated with atrial fibrillation in individuals of European ancestry. *Nature genetics* **41,** 879 (2009) (cit. on p. 7).
9. January, C. T. *et al.* 2014 AHA/ACC/HRS Guideline for the Management of Patients With Atrial Fibrillation. *Journal of the American College of Cardiology* **64,** e1–e76. ISSN: 0735-1097 (2014) (cit. on p. 7).
10. Fritsche, L. G. *et al.* Association of Polygenic Risk Scores for Multiple Cancers in a Phenome-wide Study: Results from The Michigan Genomics Initiative. *The American Journal of Human Genetics* **102,** 1048 –1061. ISSN: 0002-9297 (2018) (cit. on p. 8).
11. Roberts, M., Asgari, M. & Toland, A. Genome-wide association studies and polygenic risk scores for skin cancer: clinically useful yet? *British Journal of Dermatology* (2019) (cit. on p. 8).
12. Gellert-Kristensen, H. *et al.* Identification and Replication of Six Loci Associated With Gallstone Disease. *Hepatology* (2018) (cit. on p. 9).
13. Liu, Y. & Allingham, R. R. Major review: Molecular genetics of primary open-angle glaucoma. *Experimental Eye Research* **160,** 62 –84. ISSN: 0014-4835 (2017) (cit. on p. 9).

14. Meguro, A., Inoko, H., Ota, M., Mizuki, N. & Bahram, S. Genome-wide association study of normal tension glaucoma: common variants in SRBD1 and ELOVL5 contribute to disease susceptibility. *Ophthalmology* **117,** 1331–8 (2010) (cit. on p. 9).

15. Genome-wide Association Study of Normal Tension Glaucoma: Common Variants in SRBD1 and ELOVL5 Contribute to Disease Susceptibility. *Ophthalmology* **117,** 1331 –1338.e5. ISSN: 0161-6420 (2010) (cit. on p. 9).

16. Alward, W. L. M. *et al.* Myocilin Mutations in Patients With Normal-Tension GlaucomaMyocilin Mutations in Patients With Normal-Tension GlaucomaMyocilin Mutations in Patients With Normal-Tension Glaucoma. *JAMA Ophthalmology* **137,** 559–563. ISSN: 2168-6165 (May 2019) (cit. on p. 9).

17. Kondkar, A. A., Azad, T. A., Almobarak, F. A., Abu-Amero, K. K. & Al-Obeidan, S. A. Polymorphism rs7961953 in TMTC2 gene is not associated with primary open-angle glaucoma in a Saudi cohort. *Ophthalmic Genetics* **40.** PMID: 30729851, 74–76 (2019) (cit. on p. 9).

18. Chaiwiang, N. & Poyomtip, T. The Association of Toll-Like Receptor 4 Gene Polymorphisms with Primary Open Angle Glaucoma Susceptibility: A meta-analysis. *Bioscience Reports.* ISSN: 0144-8463. doi:`10.1042/BSR20190029`. eprint: `http://www.bioscirep.org/content/early/2019/03/15/BSR20190029.full.pdf`. `http://www.bioscirep.org/content/early/2019/03/15/BSR20190029` (2019) (cit. on p. 9).

19. Tham, Y.-C. *et al.* Aggregate Effects of Intraocular Pressure and Cup-to-Disc Ratio Genetic Variants on Glaucoma in a Multiethnic Asian Population. *Ophthalmology* **122,** 1149 –1157. ISSN: 0161-6420 (2015) (cit. on p. 9).

20. O'Brien, J. M. *et al.* Family History in the Primary Open-Angle African American Glaucoma Genetics Study Cohort. *American Journal of Ophthalmology* **192,** 239 –247. ISSN: 0002-9394 (2018) (cit. on p. 9).

21. Nannini, D. R., Kim, H., Fan, F. & Gao, X. Genetic Risk Score Is Associated with Vertical Cup-to-Disc Ratio and Improves Prediction of Primary Open-Angle Glaucoma in Latinos. *Ophthalmology* **125,** 815 –821. ISSN: 0161-6420 (2018) (cit. on p. 9).

22. Malachkova, N. & Veretelnyk, S. Correlation of rs35934224 polymorphism of TXNRD2 gene with primary open-angle glaucoma. *Archive of Ukrainian ophthalmology* **6,** 19–23 (2018) (cit. on p. 9).

23. Han, X. *et al.* Myocilin Gene Gln368Ter Variant Penetrance and Association With Glaucoma in Population-Based and Registry-Based StudiesMyocilin Gene Gln368Ter Variant Penetrance and Association With Glaucoma in Population-Based and Registry-Based StudiesMyocilin Gene Gln368Ter Variant Penetrance and Association With Glaucoma in Population-Based and Registry-Based Studies. *JAMA Ophthalmology* **137,** 28–35. ISSN: 2168-6165 (Jan. 2019) (cit. on p. 9).

24. Kuo, C.-F., Grainge, M. J., Zhang, W. & Doherty, M. Global epidemiology of gout: prevalence, incidence and risk factors. *Nature reviews rheumatology* **11,** 649 (2015) (cit. on p. 9).

25. For Blood Pressure Genome-Wide Association Studies, T. I. C. Genetic variants in novel pathways influence blood pressure and cardiovascular disease risk. *Nature* **478,** 103–109 (2011) (cit. on p. 9).

26. Salem, J.-E. *et al.* Association of Thyroid Function Genetic Predictors With Atrial Fibrillation: A Phenome-Wide Association Study and Inverse-Variance Weighted Average Meta-analysisAssociation Thyroid Function Genetic Predictors and Atrial FibrillationAssociation Thyroid Function Genetic Predictors and Atrial Fibrillation. *JAMA Cardiology* **4,** 136–143. ISSN: 2380-6583 (Feb. 2019) (cit. on p. 9).

27. Oram, R. A. *et al.* A Type 1 Diabetes Genetic Risk Score Can Aid Discrimination Between Type 1 and Type 2 Diabetes in Young Adults. *Diabetes Care* **39,** 337–344. ISSN: 0149-5992 (2016) (cit. on p. 11).

28. Sharp, S. A., Weedon, M. N., Hagopian, W. A. & Oram, R. A. Clinical and research uses of genetic risk scores in type 1 diabetes. *Current Opinion in Genetics & Development* **50.** Molecular and genetic basis of metabolic disease, 96 –102. ISSN: 0959-437X (2018) (cit. on p. 11).

29. Sharp, S. A. *et al.* Development and Standardization of an Improved Type 1 Diabetes Genetic Risk Score for Use in Newborn Screening and Incident Diagnosis. *Diabetes Care* **42,** 200–207. ISSN: 0149-5992 (2019) (cit. on p. 11).

30. Pociot, F. & Åke Lernmark. Genetic risk factors for type 1 diabetes. *The Lancet* **387,** 2331 –2339. ISSN: 0140-6736 (2016) (cit. on p. 11).

31. Mihaescu R Meigs J, S. E.J. A. Genetic risk profiling for prediction of type 2 diabetes. *PLoS Curr* **3** (2011) (cit. on p. 11).

32. Valeriya Lyssenko, M. L. Genetic Screening for the Risk of Type 2 Diabetes Worthless or valuable? *Diabetes Care* **36,** S120–S126 (2013) (cit. on p. 11).

33. Talmud, P. J. *et al.* Sixty-Five Common Genetic Variants and Prediction of Type 2 Diabetes. *Diabetes* **64,** 1830–1840. ISSN: 0012-1797 (2015) (cit. on p. 11).

34. Khera, A. V. *et al.* Genome-wide polygenic scores for common diseases identify individuals with risk equivalent to monogenic mutations. *Nature genetics* **50,** 1219 (2018) (cit. on p. 11).

35. Wu, X. & Gu, J. Heritability of prostate cancer: a tale of rare variants and common single nucleotide polymorphisms. *Annals of translational medicine* **4** (2016) (cit. on p. 11).

36. Mancuso, N. *et al.* The contribution of rare variation to prostate cancer heritability. *Nature genetics* **48,** 30 (2016) (cit. on p. 11).

37. Pashayan, N. *et al.* Polygenic susceptibility to prostate and breast cancer: Implications for personalised screening. *British journal of cancer* **104,** 1656–63 (Apr. 2011) (cit. on p. 11).

38. Lello, L. *et al.* Accurate genomic prediction of human height. *Genetics* **210,** 477–497 (2018) (cit. on pp. 12, 13).

39. Donoho, D. & Tanner, J. Observed universality of phase transitions in high-dimensional geometry, with implications for modern data analysis and signal processing. *Philosophical Transactions of the Royal Society A: Mathematical, Physical and Engineering Sciences* **367,** 4273–4293 (2009) (cit. on p. 13).

40. Vattikuti, S., Lee, J. J., Chang, C. C., Hsu, S. D. H. & Chow, C. C. Applying compressed sensing to genome-wide association studies. *GigaScience* **3,** 10. ISSN: 2047-217X (2014) (cit. on p. 13).

41. Ho, C. M. & Hsu, S. D. Determination of nonlinear genetic architecture using compressed sensing. *GigaScience* **4.** doi:10.1186/s13742-015-0081-6. https://doi.org/10.1186/s13742-015-0081-6 (Sept. 2015) (cit. on p. 13).

42. Marzban, C. The ROC Curve and the Area under It as Performance Measures. *Weather and Forecasting* **19,** 1106–1114 (2004) (cit. on p. 13).
